# On *Prophoca* and *Leptophoca* (Pinnipedia, Phocidae) from the Miocene of the North Atlantic realm: redescription, phylogenetic affinities and paleobiogeographic implications

**DOI:** 10.7717/peerj.3024

**Published:** 2017-02-21

**Authors:** Leonard Dewaele, Olivier Lambert, Stephen Louwye

**Affiliations:** 1 Department of Geology, Ghent University, Ghent, Belgium; 2 O.D. Earth and History of Life, Royal Belgian Institute of Natural Sciences, Brussels, Belgium

**Keywords:** Phocidae, *Prophoca*, *Leptophoca*, Miocene, North Atlantic, Redescription, Biostratigraphy, Phylogeny, Biogeography

## Abstract

**Background:**

*Prophoca* and *Leptophoca* represent the oldest known genera of phocine seals, dating from the latest early to middle Miocene. Originally, *Prophoca rousseaui* and *Prophoca proxima* were described based on fragmentary remains from the Miocene of Belgium. However, several researchers contested the union of *Prophoca rousseaui* and *Prophoca proxima* into one genus, without providing evidence. The stratigraphic context of *Prophoca* remained poorly constrained due to the lack of precise data associated with the original specimens collected in the area of Antwerp (north of Belgium).

**Methods:**

*Prophoca* and *Leptophoca* are redescribed and their phylogenetic position among Phocidae is reassessed using PAUP. Dinoflagellate biostratigraphy has been carried out on sediment samples associated with specimens from *Prophoca* and *Leptophoca* to elucidate their approximate ages.

**Results:**

Whereas the species *Prophoca rousseaui* is redescribed, *Prophoca proxima* is considered synonymous to *Leptophoca lenis*, with the proposal of a new combination *Leptophoca proxima* (Van Beneden, 1877). Sediment samples from specimens of both taxa have been dated to the late Langhian–early Serravallian (middle Miocene). Following a reinvestigation of *Leptophoca amphiatlantica*, characters from the original diagnosis are questioned and the specimens of *Leptophoca amphiatlantica* are considered *Leptophoca* cf. *L. proxima*. In a phylogenetic analysis, *Prophoca rousseaui* and *Leptophoca proxima* constitute early branching stem-phocines.

**Discussion:**

*Leptophoca proxima* from the North Sea Basin is younger than the oldest known find of *Leptophoca proxima* from North America, which does not contradict the hypothesis that Phocinae originated along the east coast of North America during the late early Miocene, followed by dispersal to Europe shortly after. Morphological features of the appendicular skeleton indicate that *Prophoca rousseaui* and *Leptophoca proxima* have archaic locomotory modes, retaining a more prominent use of the fore flipper for aquatic propulsion than extant Phocidae.

## Introduction

Compared to many other marine mammal taxa, extinct true seal remains (Carnivora, Phocidae) are not uncommon. However, at present, only a few areas yielded a significant number of Neogene specimens: (1) the Miocene of the Paratethys region (Koretsky, 2001), (2) the Miocene and Pliocene of the North American East Coast (True, 1906; Ray, 1976a; Koretsky & Ray, 2008), (3) the Miocene and (presumably) Pliocene of the southern North Sea Basin, including both the Belgian Antwerp area and the Netherlands (Van Beneden, 1859, 1871, 1876, 1877; Koretsky & Peters, 2008; Koretsky, Ray & Peters, 2012; Koretsky, Peters & Rahmat, 2015), and (4) the Miocene/Pliocene Pisco Formation of Peru (de Muizon, 1981; Amson & de Muizon, 2014; Valenzuela-Toro et al., 2015).

Nevertheless, specimens have also been found outside of these areas, such as in Argentina (*Properiptychus argentinus* Ameghino, 1893; de Muizon & Bond, 1982; and *Kawas benegasorum* Cozzuol, 2001), Chile (see, Walsh & Naish, 2002; Valenzuela-Toro et al., 2013; Pyenson et al., 2014; *Australophoca changorum* Valenzuela-Toro et al., 2015), Tuscany, Italy (*Pliophoca etrusca* Tavani, 1941; Berta et al., 2015; and *Monotherium gaudini* Guiscardi, 1871), Libya (*Afrophoca libyca* Koretsky & Domning, 2014), South Africa (*Homiphoca capensis* Hendey & Repenning, 1972; de Muizon & Hendey, 1980; Avery & Klein, 2011; Govender, Chinsamy & Ackermann, 2012), Alaska (Repenning, 1983), California (Barnes & Mitchell, 1975), and Australia and New Zealand (e.g., King, 1973; Fordyce & Flannery, 1983; Boessenecker & Churchill, 2016), although each of these localities only yielded either a limited amount of material (Argentina, Australia, Chile, Italy, Libya, and New Zealand) or a low diversity (South Africa).

Despite the rich fossil record of fossil Phocidae Gray, 1821, many species—most notably many Phocinae Gray, 1821—are very poorly known, based on isolated postcranial remains (e.g., Van Beneden, 1871, 1877; Koretsky, 2001; Koretsky & Peters, 2008; Koretsky & Ray, 2008; Koretsky & Rahmat, 2013; Koretsky, Peters & Rahmat, 2015).

The fossil phocid material from the Belgian Antwerp area had only been studied in-depth by P.-J. Van Beneden during the second part of the 19th century (Van Beneden, 1859, 1871, 1876, 1877). In the original description, the extinct genus *Prophoca* Van Beneden, 1877 comprised two species based on postcranial remains: *Prophoca rousseaui* Van Beneden, 1877 and *Prophoca proxima* Van Beneden, 1877. Originally dated to the Miocene ‘Anversien,’ the *Prophoca* genus is considered among the oldest known fossil seal taxa (Deméré, Berta & Adams, 2003). However, the term ‘Anversien’ was ill defined and is nowadays considered obsolete (Laga & Louwye, 2006). More recently, Louwye et al. (2010) described an innominate and a femoral capitulum from the Berchem Formation at Posthofbrug, Antwerp, Belgium. At the time, they identified both specimens as Phocidae aff. *Prophoca*. These attributions are revised here.

True (1906) was the first to question the attribution of the two species to the same genus. He observed similarities between the original illustrations of the humerus of *Prophoca proxima* (IRSNB 1146-M279; Van Beneden, 1877: Plate XVIII Figs. 12–14) and the holotype of *Leptophoca lenis* True, 1906, particularly “the peculiar feature of a thin-edged deltoid ridge” (True, 1906: 838). The latter species had been erected based on an isolated humerus from the Langhian (middle Miocene) (de Verteuil & Norris, 1996) zone 10 of the Calvert Formation from Chesapeake Bay, Calvert County, MD, USA, making it the oldest known fossil of a phocine seal (e.g., Ray, 1976a; Deméré, Berta & Adams, 2003). However, True (1906: 838) remained “uncertain as to whether it (*Prophoca proxima*) should be referred to that genus (*Leptophoca lenis*)” and he also described a right radius, a right tibia and a lumbar vertebra which he only very tentatively assigned to *Leptophoca lenis*, based on their geographical provenance and size comparison with *Leptophoca lenis*. Ray (1976a) recombined *Prophoca proxima* to *Leptophoca proxima* without providing supporting evidence, retaining *Leptophoca lenis* as a separate species. Ray (1976a) also stated both species to be primitive and considered *phoca vindobonensis* (i.e., *Praepusa vindobonensis* Koretsky, 2001 from the early Sarmatian (middle Serravallian) of the Paratethys) and *Prophoca rousseaui* “possibly referable to *Leptophoca*” (Ray, 1976a: 395). Deméré, Berta & Adams (2003) adopted Ray’s (1976a) concept of *Leptophoca proxima*. Koretsky (2001) presented a short description of *Prophoca proxima*, with the right humerus IRSNB 1146-M279 as the lectotype for the species. She did not treat *Prophoca rousseaui* in detail and considered *Prophoca rousseaui* a monachine seal, as assumed earlier (Simpson, 1945; Ray, 1976a; Koretsky, 2001).

Although the phocid subfamily Monachinae Gray, 1869 is currently largely restricted to the sub-tropical, Mediterranean, sub-Antarctic, and Antarctic environments, middle and late Miocene, Pliocene, and Quaternary fossils have been found in the North Atlantic (e.g., Koretsky & Ray, 2008) and the Paratethys (Koretsky, 2001). Members of this subfamily are characterized by a premaxilla–maxilla suture that is not visible along the entire margin of the naris in lateral view, a reduced number of upper incisors, the shortening of the third metatarsal, and reduced claws on the pes (e.g., Wyss, 1988). Currently restricted to the Arctic, sub-Arctic, and northern temperate zones, the Phocinae are grouped on the basis of the visibility of the mastoid process in dorsal view, the deltopectoral crest being short and ending abruptly above diaphysis, presence of an entepicondylar foramen on humerus, a well-developed supinator ridge on humerus, the presence of a plantar process on the cuneiform, metacarpals I and II being nearly equally large, an unreduced fifth intermediate phalanx on the manus, phalanges being round in cross-section and with trochleated heads, large claws on manus and pes, and a deep post-tibial fossa (Wyss, 1988). Given the large quantity of *Leptophoca lenis* specimens available from the east coast of North America, Koretsky (2001) presented a more thorough redescription of *Leptophoca lenis*, adding many previously unknown parts of the skeleton to the few specimens published before. Koretsky (2001) established five phocid ecomorphotypes, allowing to link isolated specimens to the same ecomorphotype and possibly also to the same taxon depending on their stratigraphic provenance. Consequently, Koretsky (2001) associated isolated specimens to *Leptophoca lenis*. However, the fossil evidence for this ecomorphotype hypothesis has not been fully provided, and its application to *Leptophoca lenis* remains should be considered with care. Given the current lack of published evidence in support of the separation of *Prophoca proxima* and *Prophoca rousseaui*, and the relationship of *Leptophoca lenis* and *Prophoca proxima*, a formal redescription and re-assessment of *Prophoca rousseaui* and *Prophoca proxima* is presented in this study.

Koretsky’s (2001) phylogenetic analysis considers both *Leptophoca lenis* and *Prophoca proxima* to be relatively late branching members of the Phocini (Gray, 1821) tribe, with *Leptophoca lenis* being a sister taxon to the clade including *Cryptophoca maeotica* (Nordmann, 1860), *Monachopsis pontica* (Eichwald, 1850), *Prophoca proxima*, the genus *Praepusa* Kretzoi, 1941, and *Sarmatonectes sintsovi* Koretsky, 2001, and with *Prophoca proxima* a sister taxon to the clade including *M. pontica*, *Praepusa*, and *S. sintsovi*. In the present study, we reassess the phylogenetic position of *Prophoca proxima* (as *Leptophoca proxima*) and present the first published phylogenetic analysis of *Prophoca rousseaui* in relation to other middle Miocene Phocidae from the Atlantic, including the Argentinean taxa *K. benegasorum* and *P. argentinus*.

The Neogene phocid specimens studied by Van Beneden were stratigraphically poorly constrained (Van Beneden, 1859, 1871, 1876, 1877). Originally, Van Beneden assigned ‘Anversien,’ ‘Diestien,’ and ‘Scaldisien’ ages to the fossil seal specimens of the Antwerp Basin. Historically, an ‘Anversien’ age was assigned to *Prophoca* (including *Prophoca proxima* and *Prophoca rousseaui*) (Van Beneden, 1876, 1877). Nowadays, it is considered that the ‘Anversien’ corresponds to the current Berchem Formation, subdivided into the Edegem Sands, Kiel Sands, and Antwerpen Sands members in the Antwerp area (de Meuter & Laga, 1976; Laga, Louwye & Geets, 2001). Louwye, Head & De Schepper (2004) attributed a (latest Aquitanian?) Burdigalian to middle Serravallian age to the Berchem Formation based on dinoflagellate cyst biostratigraphy. Sediment samples retrieved from some specimens attributed to *Prophoca proxima* (here *Leptophoca proxima* n. comb.) and *Prophoca rousseaui* were analyzed palynologically in order to assess the biostratigraphic position of the sediment based on dinoflagellate cyst biostratigraphy.

## Materials and Methods

### Specimens

Van Beneden (1877) assigned 13 partly preserved specimens to the species, including an associated pelvis and an associated femur and tibia (Table 1). However, apart from being broken and partly preserved, specimens do not show other signs of abrasion. Most notably the poor state of preservation of the lectotype humerus (IRSNB 1149-M274), with its epiphyses strongly damaged, negatively impacts its value for the diagnosis of the species. Nevertheless, the overall state of preservation of each specimen still renders them diagnostic. Two of these specimens, one lumbar vertebra and the fibula, are now missing in the IRSNB collection; and neither specimen has been illustrated (see Van Beneden, 1877). *Prophoca proxima* as defined by Van Beneden (1877) only yielded a collection of five specimens at the IRSNB (Van Beneden, 1877) (Table 2). However, the current study synonymizes *Prophoca proxima* with *Leptophoca lenis*, which is questionably the most completely described fossil phocine seal (Koretsky, 2001). However, as noted above, the attribution of different isolated bones to *Leptophoca lenis* remains doubtful.

**Table 1:** Specimen list of *Prophoca*. Specimens from the IRSNB collection attributed to *Prophoca*, with original publication of each specimen.

Collection number	Original description	Current description	Specimen	Locality	Original Stratigraphy	Publication
IRSNB 1147-M275	*Prophoca rousseaui*	*Prophoca rousseaui*	Radius (L)	Borsbeek, Fort no. 3	Miocene, ‘Anversien’	Van Beneden (1877)
IRSNB 1149-M274	*Prophoca rousseaui*	*Prophoca rousseaui*	Humerus (R) (Lectotype)	Borsbeek, third section	Miocene, ‘Anversien’	Van Beneden (1877)
IRSNB 1150-M277	*Prophoca rousseaui*	*Prophoca rousseaui*	Femur (R, 277a), Tibia (R, 277b)	Antwerp, second section	Miocene, ‘Anversien’	Van Beneden (1877)
IRSNB 1192-M276	*Prophoca rousseaui*	*Prophoca rousseaui*	3 Lumbar Vertebrae (276a), 2 innominates (L, 276b; R, 276d), sacrum (276c)	Antwerp, third section	Miocene, ‘Anversien’	Van Beneden (1877)
IRSNB-VERT-3250-15	*Prophoca rousseaui*	*Prophoca rousseaui*	Tibia (L)	Berchem	Miocene, ‘Sable à héterocètes’	Van Beneden (1877)
N/A	Phocidae aff. *Prophoca*	Phocidae indet.	Femur (capitulum, indet. side)	Posthofbrug, Berchem, Antwerp	Antwerpen Sands, Berchem Formation	Louwye et al. (2010)
IRSNB M2234	Phocidae aff. *Prophoca*	*Prophoca rousseaui*	Innominate (L)	Posthofbrug, Berchem, Antwerp	Antwerpen Sands, Berchem Formation	Louwye et al. (2010)
N/A	*Prophoca rousseaui*	Lost	Lumbar vertebra	N/A	N/A	Van Beneden (1877)
N/A	*Prophoca rousseaui*	Lost	Fibula	N/A	N/A	Van Beneden (1877)

**Note:**

L, left; R, right.

**Table 2:** Specimen list of *Leptophoca*. Specimens of *Leptophoca proxima* n. comb and Phocinae aff. *Leptophoca proxima* in the IRSNB collection, with original publication of each specimen.

Collection number	Original identification	Current identification	Specimen	Locality	Stratigraphy	Publication
IRSNB 1146-M279	*Prophoca proxima*	*Leptophoca proxima*	Humerus (L)	Borgerhout, third section	Miocene, ‘Anversien’	Van Beneden (1877)
IRSNB M2233	*Prophoca proxima*	Phocinae aff. *Leptophoca proxima*	Humerus (R)	Unknown	Unknown	Van Beneden (1877)
IRSNB 1145-M 280	*Prophoca proxima*	*Leptophoca proxima*	Ulna (L, 280a), radius (L, 280b)	Borsbeek, third Fort	Miocene, ‘Anversien’	Van Beneden (1877)
IRSNB M2232	*Prophoca proxima*	Phocinae aff. *Leptophoca proxima*	Fibula (R)	Antwerp	Miocene, ‘Anversien’	Van Beneden (1877)

**Note:**

L, left; R, right.

Not considering the specimen list from Supplemental Information 2, specimens observed are: *Prophoca rousseaui* (lectotype IRSNB 1147-M275, IRSNB 1149-M274, 1150-M277a-b, IRSNB 1192-M276a-d, IRSNB M2234, and IRSNB-VERT-3250-15), Phocidae aff. *Leptophoca proxima* (IRSNB M2232, IRSNB M2233), *Leptophoca proxima* (lectotype IRSNB 1146-M279, IRSNB 1145-M280a-b, USNM 5359, USNM 5361, USNM 23224, USNM 23243, USNM 23450, USNM 175578, USNM 186990, USNM 205499, USNM 263648, USNM 284721, USNM 305247 cast, USNM 321934, USNM 411889, USNM 412115, USNM 454770; as *Leptophoca amphiatlantica* Koretsky, Ray & Peters, 2012: USNM 23227, MAB 2129, USNM 214897, USNM 321926), *Acrophoca longirostris* de Muizon, 1981 (all specimens from de Muizon, 1981), *C. maeotica* (USNM 489174 cast, USNM 489179 cast), *Cystophora cristata* (Erxleben, 1777) (USNM 118962, USNM 550411), *Erignathus barbatus* Erxleben, 1777 (USNM 500250, USNM 500251), *Halichoerus grypus* (Fabricius, 1791) (IRSNB 12550, IRSNB 34548), *Histriophoca fasciata* (Zimmermann, 1783) (USNM 504959, USNM 504960, USNM 571367), *Hydrurga leptonyx* (de Blainville, 1820) (IRSNB 15388), *Leptonychotes weddellii* (Lesson, 1826) (IRSNB 15390) *Lobodon carcinophaga* (Hombron & Jacquinot, 1842) (IRSNB 13307), *M. pontica* (USNM 1802 cast, USNM 214967 cast), *Monachus monachus* Hermann, 1779 (IRSNB 1153), *Ommatophoca rossii* (Gray, 1844) (IRSNB 1164), *Pagophilus groenlandicus* (Erxleben, 1777) (IRSNB 1555D) *Phoca vitulina* Linnaeus, 1758 (IRSNB 39043), *Phoca vitulinoides* Van Beneden, 1871 (IRSNB, unpublished material), *Piscophoca pacifica* de Muizon, 1981 (all specimens from de Muizon, 1981), *Praepusa vindobonensis* (Toula, 1897) (USNM cast of humerus without number, USNM 214964 cast, USNM 214993 cast), *Pusa caspica* (Gmelin, 1788) (USNM 341615), *Pusa sibirica* (Gmelin, 1788) (IRSNB, 15264, IRSNB 21171), and *S. sintsovi* (USNM 1713/146 cast, USNM cast of femur without number).

Observations of outgroup OTUs included in the phylogenetic analysis is based on observations and descriptions in the relevant literature.

### Measurements

Measurements were taken to the nearest 0.1 mm, using analog calipers. For reasons of consistency, these measurements were taken following the same scheme as Koretsky (2001), which has also been applied to *P. etrusca* more recently (Berta et al., 2015). Measurements are presented in Supplemental Information 1.

### Terminology

In order to be consistent with other recent publications on fossil Phocidae, we adopted the nomenclature and terminology used by Amson & de Muizon (2014) and Berta et al. (2015). Whenever it was not possible to refer to Amson & de Muizon (2014) or to Berta et al. (2015), we adopted the nomenclature and terminology for the osteological description of the domestic dog by Evans & De Lahunta (2013).

### Phylogenetic analysis

The phylogenetic analysis was performed using PAUP version 4.0b10 for Macintosh (Swofford, 2002) with a heuristic search option (each with 10 random additional sequences), optimized by ACCTRAN using the tree-bisection-reconnection algorithm. Bootstrap values were obtained after 10,000 replications.

Formerly, different character matrices resulting in different phylogenetic trees have been used to elucidate the phylogenetic relationships among Phocidae (see, e.g., Bininda-Emonds & Russell, 1996; Koretsky, 2001; Koretsky & Rahmat, 2013; Amson & de Muizon, 2014; Berta et al., 2015). Koretsky (2001) and Koretsky & Rahmat (2013) provided the only phylogenetic analyses on morphological characters focusing on Phocinae. In this study, we use 89 morphological characters: 88 are adopted or adapted from Amson & de Muizon (2014), Berta & Wyss (1994) and Berta et al. (2015), and references therein; and one new character (54) is introduced (Supplemental Information 4). Characters from Berta et al. (2015) and the additional character were selected because they could be scored for *Prophoca rousseaui.* All 89 characters have equal weight and one is parsimony-uninformative (36). Three characters (5, 23, and 83) are ordered. A significant number of the phylogenetic characters scored by Koretsky (2001) and Koretsky & Rahmat (2013) are prone to subjective scoring (e.g., character states ‘deep’ versus ‘shallow’). Therefore, only a limited number of those characters used by Koretsky (2001) and Koretsky & Rahmat (2013) have been adopted or adapted from these works for the current analysis (Supplemental Information 4); all of the selected characters from these two studies were also used in Amson & de Muizon (2014) and Berta et al. (2015), but have not necessarily been scored identically.

The analysis includes 24 taxa. Outgroups include the early Miocene pinnipedimorph *Enaliarctos mealsi* Mitchell & Tedford, 1973, the extant walrus *Odobenus rosmarus* Linnaeus, 1758, the extinct otariid *Thalassoleon mexicanus* Repenning & Tedford, 1977, the extant otariids *Otaria byronia* Blainville, 1820 and *Arctocephalus pusillus* (Schreber, 1775), and the desmathophocidae *Allodesmus kernensis* Kellogg, 1922. Ingroup taxa include the extinct monachines *A. longirostris*, *Callophoca obscura* Van Beneden, 1877, *Hadrokirus martini* Amson & de Muizon, 2014, *H. capensis*, and *P. pacifica*; the extant monachines *M. monachus*, *Mirounga leonina* (Linnaeus, 1758) (southern elephant seal), *H. leptonyx*, *L. carcinophaga*, *O. rossii*, and *L. weddellii*; the extinct Atlantic middle Miocene phocines *Leptophoca proxima*, *Prophoca rousseaui*, and *K. benegasorum*; and the extant phocines *E. barbatus*, the gray seal *H. grypus*, and *Phoca vitulina*. Unlike Amson & de Muizon (2014), we did not score the skull of *Callophoca* (USNM 475486) because it represents an isolated specimen that is only very tentatively assigned to the species (see Koretsky & Ray, 2008). We follow Barnes (1972) in considering *Allodesmus kelloggi* a junior synonym to *Allodesmus kernensis*.

*Prophoca rousseaui* and postcranial specimens of *Leptophoca proxima* were scored after on-hand observations. In the phylogenetic analysis, we did neither score mandibular nor cranial characters of *Leptophoca proxima*. Indeed, mandibular and cranial specimens from the CMM and USNM assigned to *Leptophoca proxima* have all been found as isolated specimens and are assigned to the species on the basis of their stratigraphical provenance. Similarly, all other postcranial specimens have been found isolated, but the relative abundance of different postcranial specimens from the same strata renders it slightly more probable that they belong to the same species, i.e., *Leptophoca proxima*. Hence, relatively abundant postcranial specimens of *Leptophoca proxima*, such as the humerus and the femur, are included in the analysis. Scoring of *Allodesmus kernensis*, *E. mealsi*, and *T. mexicanus* is based on descriptions and illustrations in existing literature (Mitchell, 1966; Barnes, 1972; Mitchell & Tedford, 1973; Repenning & Tedford, 1977; Berta & Ray, 1990; Deméré & Berta, 2005).

### Nomenclatural acts

The electronic version of this article in portable document format will represent a published work according to the International Commission on Zoological Nomenclature (ICZN), and hence the new names contained in the electronic version are effectively published under that Code from the electronic edition alone. This published work and the nomenclatural acts it contains have been registered in ZooBank, the online registration system for the ICZN. The ZooBank LSIDs (Life Science Identifiers) can be resolved and the associated information viewed through any standard web browser by appending the LSID to the prefix http://zoobank.org/. The LSID for this publication is: urn:lsid:zoobank.org:pub:23248E40-CFED-4E41-912B-2702480A67B6. The online version of this work is archived and available from the following digital repositories: PeerJ, PubMed Central, and CLOCKSS.

## Results

### Geological and stratigraphical setting

**Figure 1:**
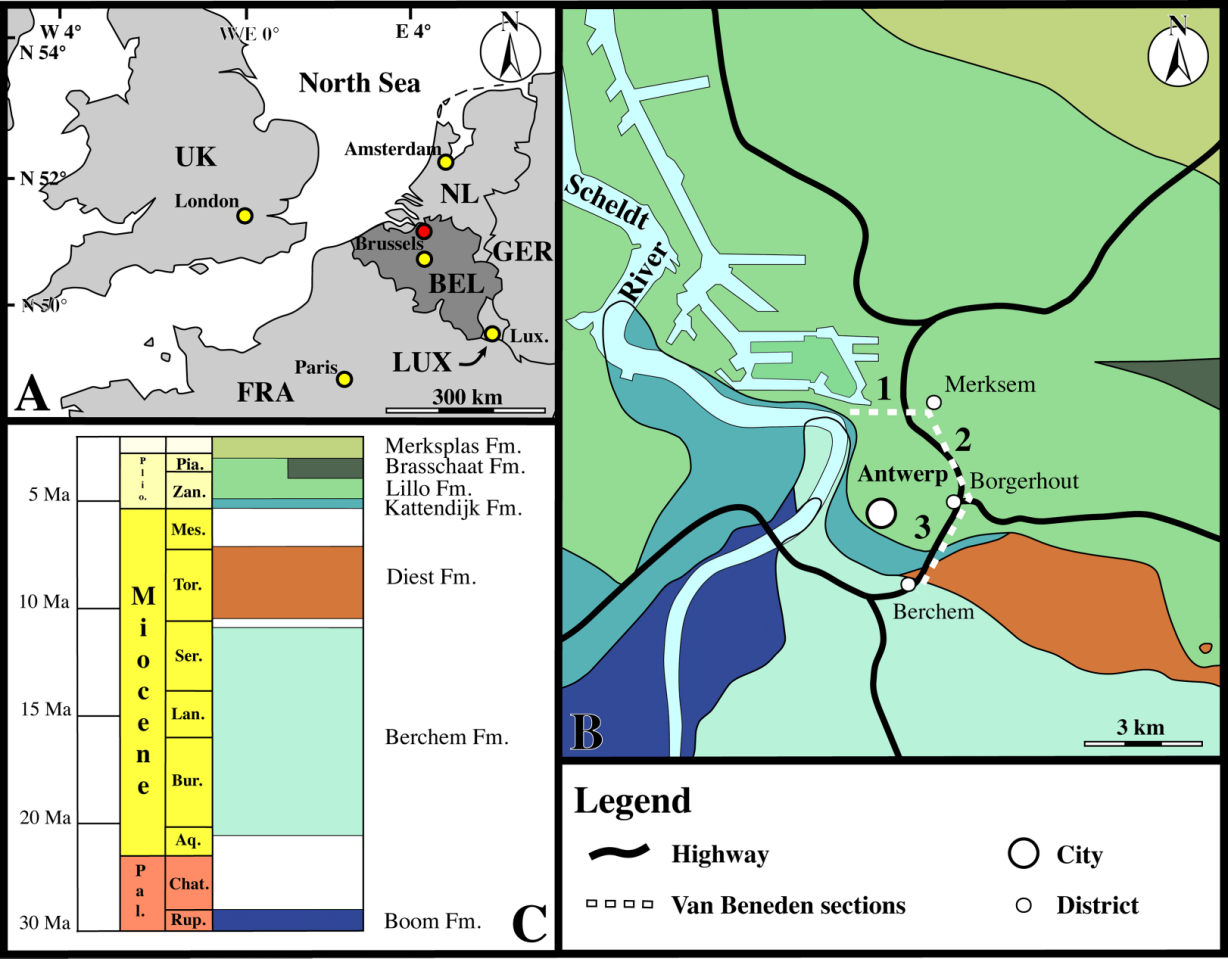
Locality. (A) Regional map showing the southern part of the North Sea basin with bordering countries and labeled capital cities (yellow) and the Antwerp area (red). (B) Close-up of the Antwerp area with color-coding for the outcropping Paleogene and Neogene strata underneath the Quaternary top layer. The sections of the fortification walls around Antwerps used by Van Beneden (1877) as localities for the Neogene marine mammals (including seals) from the Antwerp area are indicated by dashed lines and numbered as in Van Beneden (1877), using Vanden Broeck (1878). The ‘third section’ of Van Beneden (1877) is the type locality for both *Prophoca rousseaui* and *Leptophoca proxima*. (C) Stratigraphic legend for the Paleogene and Neogene strata from the Antwerp Area. Abbreviations: NL, Netherlands; GER, Germany; LUX, Luxembourg; FRA, France; UK, United Kingdom; BEL, Belgium; Lux., Luxembourg City; Plio., Pliocene; Pal., Paleocene; Pia., Piacenzian; Zan., Zanclean; Mes., Messinian; Tor., Tortonian; Ser., Serravallian; Lan., Langhian; Bur., Burdigalian; Aq., Aquitanian; Chat., Chattian; Rup., Rupelian; Fm., Formation. Image based on data from Dienst Ondergrond Vlaanderen (DOV; dog.vlaanderen.be).

The original Van Beneden collection of fossil Phocidae was collected during fortification works around Antwerp, during the 1850s (Van Beneden, 1871, 1876, 1877). Many specimens had been collected from old forts or trenches roughly coinciding with the current R10 highway around Antwerp. The geographic and stratigraphic context of many specimens is relatively poorly constrained, given the profound lateral stratigraphic variation in the Neogene of the Antwerp region. Van Beneden usually referred to specific ‘sections’ as the localities of his specimens. Vanden Broeck (1878) stated that each ‘section’ mentioned by Van Beneden corresponds to a specific section of the defensive trench around Antwerp. The second and Third sections, the areas of origin of the Belgian specimens of *Prophoca rousseaui* and *Leptophoca proxima*, roughly coincide with the north and northeast sections of the current R10 highway, respectively. The third section, the area of origin for the lectotypes of *Prophoca rousseaui* and *Leptophoca proxima* is indicated in Fig. 1.

Three sediment samples recovered from bone cavities (from *Prophoca rousseaui* tibia IRSNB 1150-M277b and lumbar vertebrae IRSNB 1192-M276 and *Leptophoca proxima* humerus IRSNB 1146-M279) were palynologically analyzed in order to propose a relative dating of these sediments based on dinoflagellate cyst (dinocyst) biostratigraphy. As such, the relative dating of the sediments provides a minimum age for the fossils.

The biostratigraphic assessment and relative dating is based on calibrated stratigraphic ranges of dinocysts key species in the North Sea Basin and the North Atlantic realm (de Verteuil & Norris, 1996; Dybkjær & Piasecki, 2010; Quaijtaal et al., 2014; Schreck, Matthiesen & Head, 2012). Full details regarding the assemblage composition, biostratigraphic interpretation, and relative dating are given in Supplemental Information 2.

The relative dating of the sample taken from *Prophoca rousseaui* IRSNB 1150-M277b relies on the joint occurrences of *Habibacysta tectata* (lowest occurrence dated at 14.2 Ma) and *Hystrichosphaeropsis obscura* (highest occurrence dated at 7.6 Ma). A late Langhian to late Tortonian age can thus be inferred for this sample. The sample taken from *Prophoca rousseaui* IRSNB 1192-M276 holds both dinocyst species *H. tectata* and *Unipontedinium aquaeductus.* The latter species has a well-delimited occurrence in the middle Miocene from 14.8 to 13.2 Ma. An age between 14.2 and 13.2 Ma can thus be proposed (late Langhian–early Serravallian). Dinocyst diversity in the sample from *Leptophoca proxima* IRSNB 1146-M279 is low and the preservation is moderate to poor. Few age diagnostic species are present apart from *U. aquaeductus.* A middle Langhian to early Serravallian age (14.8–13.2 Ma) can be deduced.

### Systematic paleontology

Family PHOCIDAE Gray, 1825
Subfamily PHOCINAE Gray, 1821
*Prophoca* Van Beneden, 1877

**Type and only included species—***Prophoca rousseaui* Van Beneden, 1877 by subsequent designation by Kellogg (1922: 116) following Article 69 of the ICZN.

**Diagnosis—**Same as for the only included species.

*Prophoca rousseaui* Van Beneden, 1877

**Figure 2:**
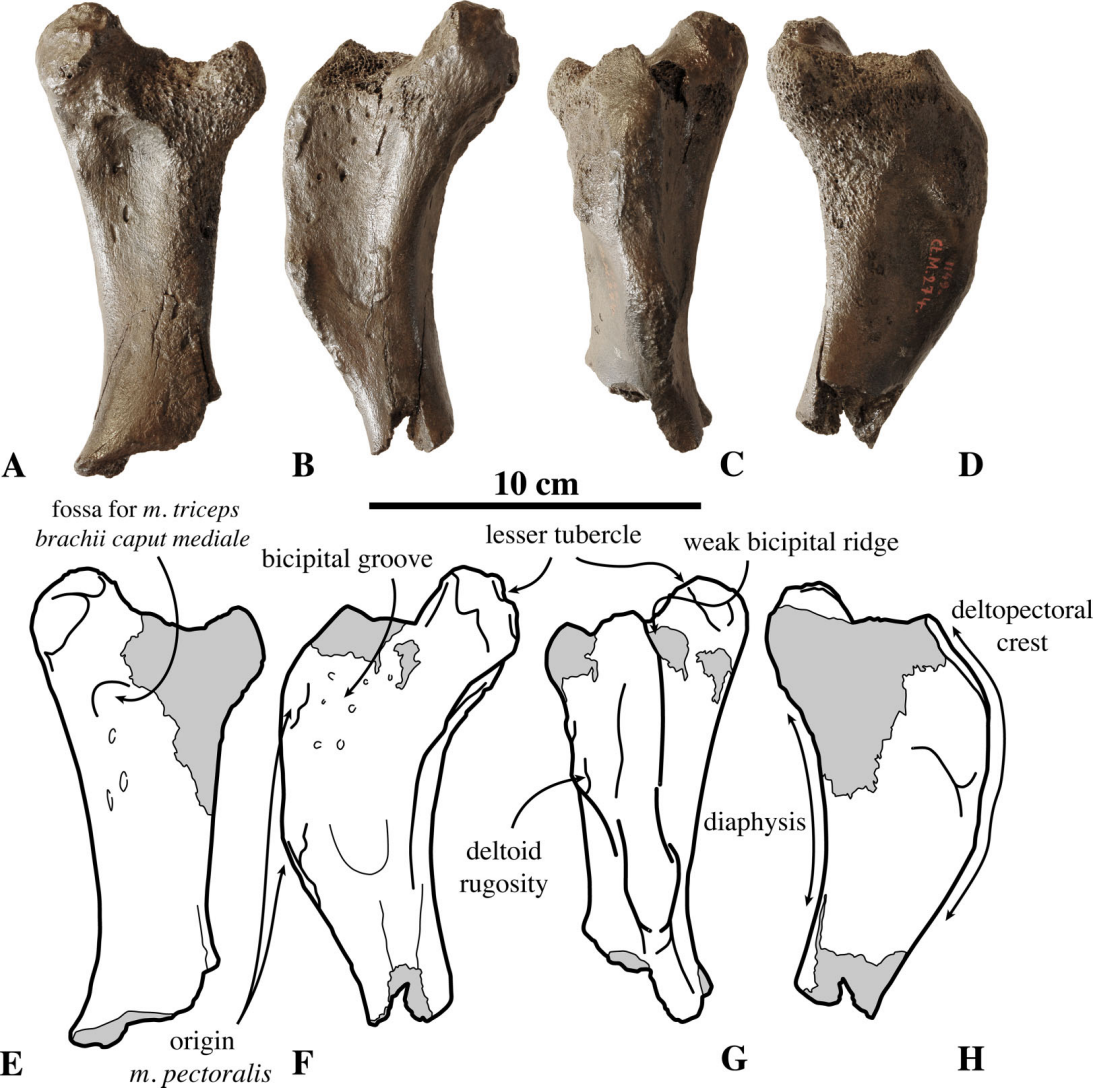
Humerus of *Prophoca rousseaui*. IRSNB 1149-M274 lectotype right humerus of *Prophoca rousseaui* (A–D) and corresponding drawings (E–H) in posterior (A, E), medial (B, F), anterior (C, G) and lateral (D, H) view.

**Lectotype:** Right humerus IRSNB 1149-M274. Illustrated and described by Van Beneden (1877, Plate XVIII, Figs. 1 and 2).

**Type locality:** ‘Borsbeek, third Section.’ This section of the former fortification ring around the city of Antwerp runs from the district of Deurne to the former ‘porte de Borsbeek.’ The entire third section roughly coincides with the current section of the R10 highway east of Antwerp, running from Deurne to the Borsbeekbrug (formerly ‘porte de Borsbeek’), near the Antwerpen–Berchem railroad station (Fig. 1) (Vanden Broeck, 1878). The Borsbeekbrug neighborhood (51°12′02″N, 4°26′13″E Web Mercator) encompasses the southern portion of this section.

**Type horizon and age:** ‘Anversien’ (Van Beneden, 1876, 1877). The precise location and stratigraphical position is unknown. Deméré, Berta & Adams (2003) reassigned the specimen to the Berchem Formation without providing any evidence.

Biostratigraphical analysis with dinoflagellate cysts of a sediment sample from the lumbar vertebrae referred to *Prophoca rousseaui* IRSNB 1192-M276a provided a late Langhian–early Serravallian age (14.2–13.2 Ma). Similarly, a sediment sample from a femur referred to *Prophoca rousseaui* IRSNB 1150-M277b gives a late Langhian–late Tortonian age (14.2–7.2 Ma). The tibia IRSNB-VERT-3250-15 had been assigned to the so-called ‘Sables à Héterocètes’ from the upper Miocene, presumed to postdate the ‘Anversien’ (see Louwye et al., 2010). The best-constrained sample provides thus a late Langhian–early Serravallian (middle Miocene) age for *Prophoca rousseaui*. Given the highly diachronous nature of the Neogene strata from Belgium, it is difficult to tie the lectotype specimen of *Prophoca rousseaui* to a specific lithostratigraphic unit through biostratigraphic age determination alone. The sediment samples from which the dinoflagellate cysts were recovered were small, but they consist of gray sand rich in white mollusk shell fragments, similar to the lithology of the Antwerpen Sands Member of the Berchem Formation (Laga, Louwye & Geets, 2001; Louwye et al., 2010). A tentative assignment of the lectotype of *Prophoca rousseaui* to the Berchem Formation would correspond to the conclusions of Deméré, Berta & Adams (2003).

**Comments:** Although first mentioned by Van Beneden in 1876, *Prophoca rousseaui* remained undescribed and, hence, a nomen nudum until its formal description one year later (Van Beneden, 1877). Neither Van Beneden (1877) nor later researchers did assign any type specimen to *Prophoca rousseaui*. Therefore, we propose the right humerus of the original description (IRSNB 1149-M274) as the lectotype, because isolated humeri and femora are the most useful postcranial bones for identifying extinct phocid species when associated cranial bones are lacking (Koretsky, 2001). Mitchell (1961) similarly discussed the taxonomic utility of otarioid humeri. Other specimens, such as the partially articulated pelvis (IRSNB 1192-M276a-d), leave little possibility for comparison with many other fossil species lacking any known pelvic bone. Also, Van Beneden (1877) did not present either *Prophoca rousseaui* or *Prophoca proxima* as the type species for the genus *Prophoca. Prophoca rousseaui* was only later named the type species for the genus (Kellogg, 1922).

Ray (1976a) also identified one specimen from the Calvert Formation as belonging to *Prophoca rousseaui*. However, we could not retrace it at the USNM. Hence, we will not consider it in our description and comparison.

**Emended diagnosis:** Large phocine seal, similar in size to the extant (monachine) Weddell seal, *L. weddellii. Prophoca rousseaui* differs from all other phocines in the following combination of characters: humeral capitulum higher than lesser tubercle (also present in *Leptophoca proxima*, *Praepusa vindobonensis* and *S. sintsovi*) with an intertubercular groove wide and shallow (also present in hooded seal *C. cristata*, bearded seal *E. barbatus* and *Leptophoca proxima*); deltopectoral crest reaching distal to half-length of diaphysis, not strongly projecting anteriorly, and distal termination rather smooth (also present in *C. maeotica*, *Leptophoca proxima*, *Phoca vitulinoides*, *Prae. vindobonensis* and *S. sintsovi*); ventral margin of wings of the sacrum straight; ilium slightly everted laterally and gluteal fossa not developed (also present in *C. cristata* and *E. barbatus*); ilium strongly elongate; ischiac tuberosity highly raised and anteroposteriorly elongate; greater trochanter rounded; wide and shallow intercondylar groove (also present in *E. barbatus*, *Prae. vindobonensis* and *S. sintsovi*); epicondylar crests of femur transversely thick but little raised.

**Referred specimens:** IRSNB 1149-M274, lectotype, right humerus, from section no. 3 at Borsbeek, Antwerp, Belgium, Miocene, ‘Anversien;’ IRSNB 1147-M275, left radius, from fort no. 3 at Borsbeek, Miocene, ‘Anversien;’ IRSNB 1192-M276a-d, three lumbar vertebrae and sacrum and left and right innominate, from section no. 3 at Antwerp, Antwerp, Miocene, ‘Anversien;’ IRSNB M2234, left innominate, from Posthofbrug, Antwerp, Antwerpen Sands Member, Berchem Formation; IRSNB 1150-M277a-b, right femur and right tibia, from section no. 2 at Antwerp, Miocene, ‘Anversien;’ IRSNB-VERT-3250-15, left tibia, from Berchem, Antwerp, Miocene, ‘Sables à Héterocètes.’

### Description

#### Appendicular skeleton

**Humerus (Fig. 2)**: The lectotype IRSNB 1149-M274 is the only humerus currently known for *Prophoca rousseaui.* This isolated humerus is poorly preserved, missing the distal extremity, and with a capitulum which is nearly completely abraded.

The humerus is relatively slender, with a size comparable to that of *L. weddellii*. Although the proximal part of the deltopectoral crest is abraded, it appears that the partially preserved bicipital groove is wide and deep. In other Phocinae, the bicipital groove tends to be much narrower than in *Prophoca rousseaui*. In Monachinae, the bicipital groove is generally very wide and shallow. To some extent, there is a low-raised bicipital ridge connecting the lesser tubercle and the deltopectoral crest. A bicipital ridge has been previously described in the monachine crabeater seal *L. carcinophaga* and, to a lesser extent, in the monachines *Monachus* Fleming, 1822, *Monotherium* Van Beneden, 1877, and *P. pacifica* (de Muizon, 1981).

The lesser tubercle is moderately well developed, proportionally slightly smaller than the lesser tubercle in the extinct monachine *P. etrusca* (see Berta et al., 2015). It is definitely not as large as in most extant Phocidae, except *Monachus* spp., but comparable in dimension to a number of Neogene phocids, including the monachines *A. longirostris* and *C. obscura* (Leonard Dewaele, 2016, personal observation; see, e.g., Van Beneden, 1877; de Muizon, 1981; Koretsky & Ray, 2008). The lesser tubercle is roughly cylindrical, with a circular scarred surface for the attachment of *m. subscapularis* facing posteriorly.

**Figure 3:**
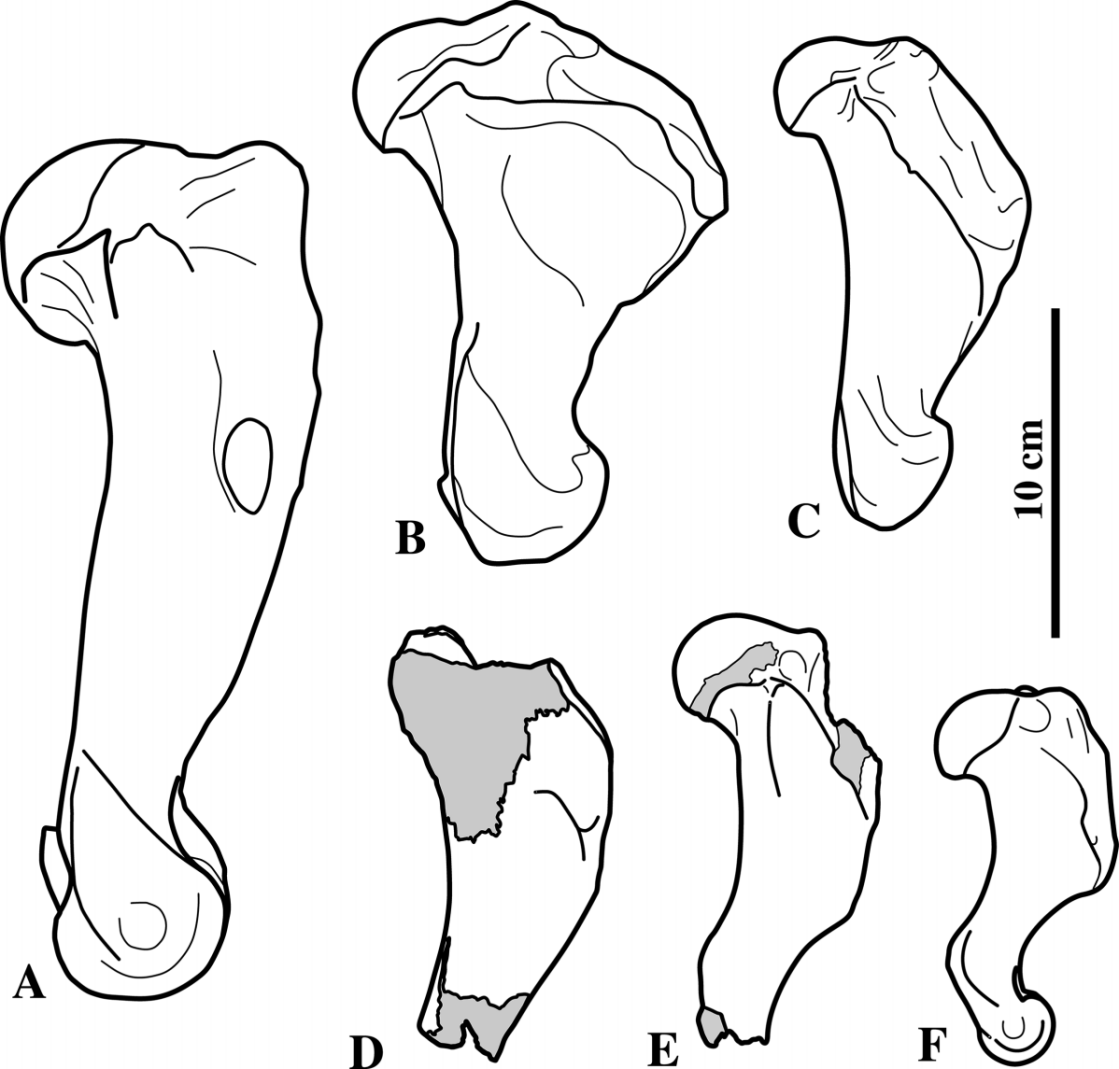
Deltopectoral crest variation in pinnipeds. Comparison of the humerus of (A) the walrus *Odobenus rosmarus*, (B) the extant monachine *Leptonychotes weddellii*, (C) the extinct monachine *Acrophoca longirostris*, the extinct phocines (D) *Prophoca rousseaui* and (E) *Leptophoca proxima*, and the extant phocine (F) the gray seal *Halichoerus grypus. Prophoca rousseaui* is humerus IRSNB-M274 and *Leptophoca proxima* is humerus IRSNB 1146-M279 described in this study. *Leptophoca proxima* mirrored left humerus. All other humeri in right lateral view. Images of *L. weddellii* and *A. longirostris* adapted from Valenzuela-Toro et al. (2015; Fig. 4).

Compared to extant Phocidae, the deltopectoral crest is typically monachine in *Prophoca rousseaui* in that it distally gradually curves toward the medial epicondyle (see Hendey & Repenning, 1972). In all extant Phocinae, the deltopectoral crest has an abrupt distal termination. However, many extinct Phocinae, such as *C. maeotica*, *K. benegasorum*, *Phoca vitulinoides*, and *S. sintsovi* (Cozzuol, 2001; Koretsky, 2001; Leonard Dewaele, 2016, personal observation) show varying conditions that are intermediate between extant non-phocid pinnipeds (*Odobenus* Brisson, 1762 and Otariidae Gray, 1825), monachines and extant phocines (Fig. 3). From what is present of the distal portion of the humerus, the deltopectoral crest of *Prophoca rousseaui* (and *Leptophoca proxima*) appears to terminate near the distal end of the diaphysis, as in *O. rosmarus*, otariids, and monachines (except *L. weddellii* and *L. carcinophaga*) and a number of extinct phocines (e.g., *C. maeotica*, *K. benegasorum*, *Phoca vitulinoides*, and *S. sintsovi*). On the other hand, the distal end of the deltoid surface is clearly demarcated by a weak but abrupt angle in the deltopectoral crest, as in Phocinae, instead of a smooth and gradual distal termination. An abrupt distal termination of the deltoid surface is typical for extant phocines (Hendey & Repenning, 1972), where it is even more pronounced than in *Prophoca rousseaui*. Also, in *Prophoca rousseaui* the deltopectoral crest is only moderately raised over the diaphysis, contrasting with the very large and prominent deltopectoral crest in most extant Phocidae. The deltopectoral crest is less developed in non-phocid pinnipeds, the extant monachine *Monachus* spp., and the extinct monachines *A. longirostris*, *Monotherium* spp., *P. pacifica* and *P. argentinus* (de Muizon, 1981; de Muizon & Bond, 1982). de Muizon (1981) also mentions *P. etrusca*, but this disagrees with a subsequent description (Berta et al., 2015). The humeri of many other extinct Phocidae display an intermediate state between these extinct and extant Phocidae. The state of preservation does not allow measuring the deltopectoral angle (*sensu* de Muizon, 1981). On the lateral side of the deltopectoral crest, the robust deltoid rugosity corresponds to the raised insertion area for the *m. brachioradialis*. The deltopectoral crest does not overhang the diaphysis laterally, differing from most extant Phocinae (except *C. cristata* and to a lesser extent also *E. barbatus*). Another prominence on the medial side of the deltopectoral crest and distal to the brachioradialis prominence serves for the attachment of the *m. pectoralis*.

On the posterior surface of the diaphysis, just distal to the damaged capitulum and the lesser tubercle, there is a shallow fossa for the origin of the *m. triceps brachii caput mediale*; a similar fossa is described in *Monotherium* spp. and *P. etrusca* (de Muizon, 1981).

**Figure 4:**
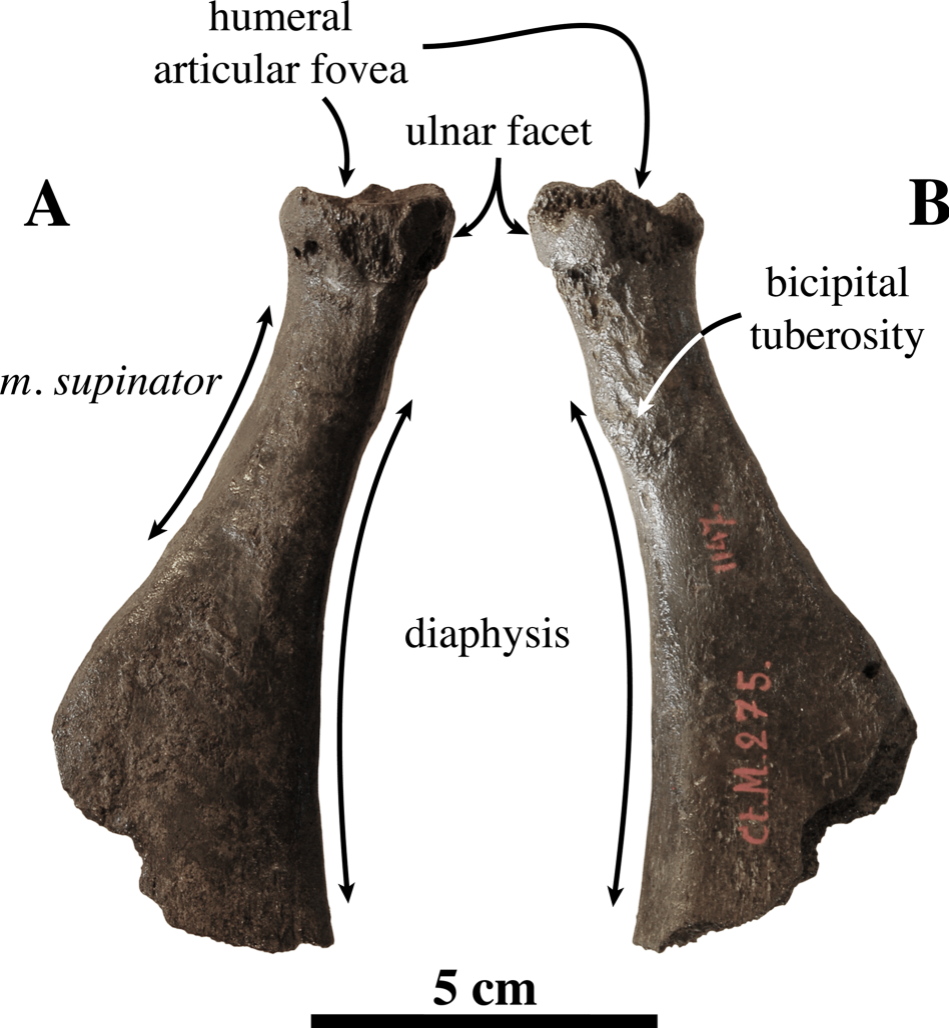
Radius of *Prophoca rousseaui*. IRSNB 1147-M275 left radius of *Prophoca rousseaui* in lateral (A) and medial (B) view.

**Radius (Fig. 4)**: The proximal part of an isolated radius with the circumference of the humeral articular fovea noticeably damaged (IRSNB 1147-M275) has been tentatively assigned to *Prophoca rousseaui*, based on its markedly different shape compared to the radius of the contemporary *Leptophoca proxima* IRSNB 1145-M280a: IRSNB 1147-M275 smoothly widens anteroposteriorly in its distal region while the widening is much less pronounced in IRSNB 1145-M280a from *Leptophoca proxima* and the diaphysis of the radius of the latter forms an angle at the level of the bicipital tuberosity. Hence, with currently no phocid species other than *Leptophoca proxima* and *Prophoca rousseaui* known from the Berchem Formation, radius IRSNB 1147-M275 can tentatively be assigned to *Prophoca rousseaui*.

The preserved portion is robust, as in Monachinae, and the diaphysis gradually widens anteroposteriorly. Although incompletely preserved, the ulnar facet on the medial margin of the humeral articular fovea is prominent. The bicipital tuberosity is smooth and oval in outline, relatively little developed, and located medial to posteromedial on the proximal portion of the diaphysis. In other Phocinae, the location of the bicipital tuberosity is variable, while this tuberosity is located medially in Otariidae Gray, 1825, and the monachines *P. argentinus*, and *P. pacifica* and located posteromedially in other Monachinae. This tuberosity is not located as high proximally as in *P. etrusca* (Berta et al., 2015), but a little separated from the proximal epiphysis, as in the extant Phocinae, the extant monachine Mediterranean monk seal *M. monachus*, and the extinct monachines *A. longirostris* and *P. pacifica* (de Muizon, 1981; Amson & de Muizon, 2014). The diaphysis is straight along the bicipital tuberosity, similar to Monachinae. In Phocinae, the diaphysis exhibits a pronounced obtuse angle at the level of the bicipital tuberosity, delineating the location of the oblong rugosity.

The origin of *m. supinator* is clearly outlined on the anterior margin of the diaphysis, indicating a rather strong development of this muscle. This corresponds with observations in extant and extinct Phocidae, except Lobodontini Gray, 1869, and Otariidae (de Muizon, 1981).

**Figure 5:**
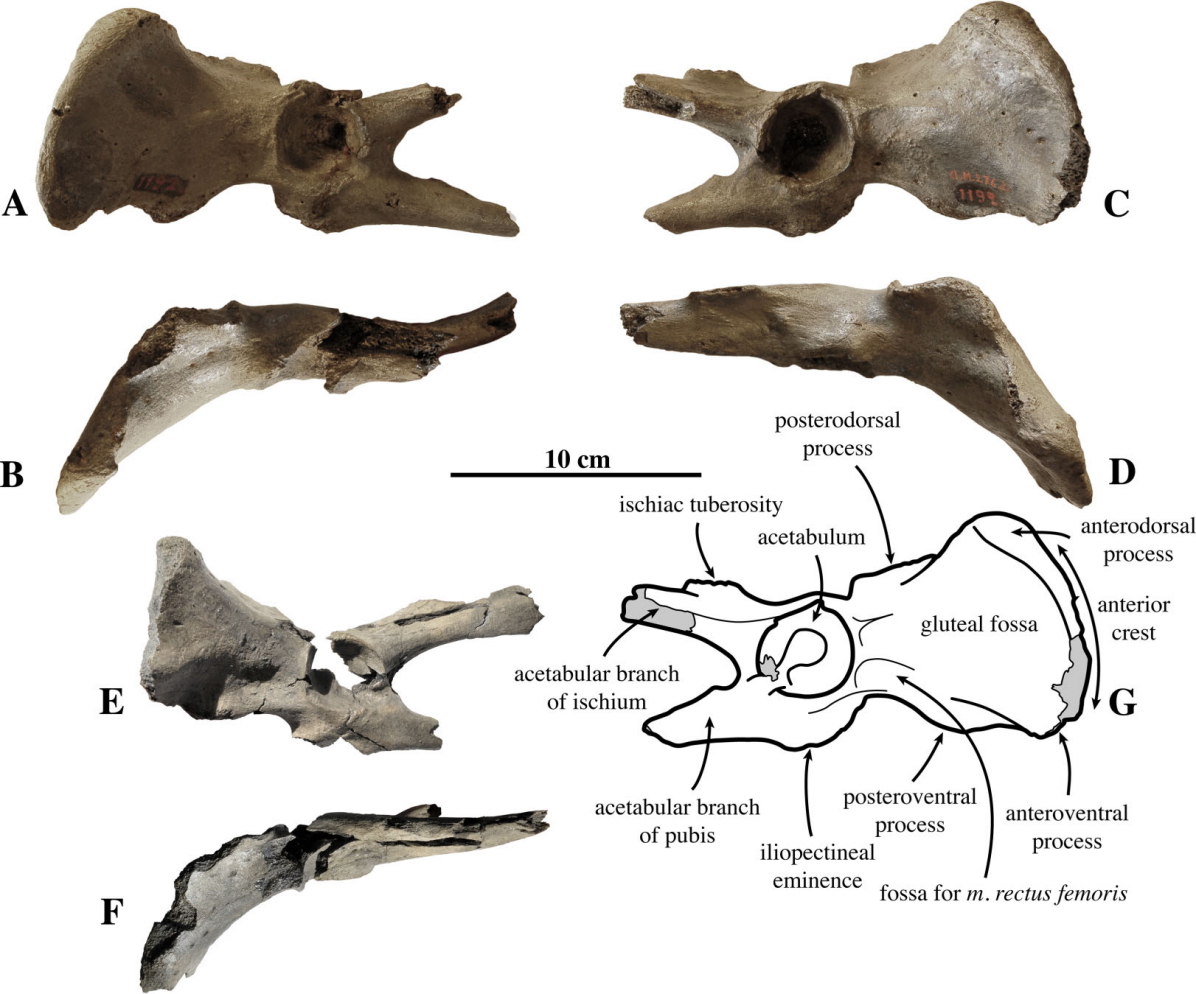
Innominate of *Prophoca rousseaui*. IRSNB 1192-M276b left innominate of *Prophoca rousseaui* in lateral (A) anddorsal (B) view; IRSNB 1192-M276d right innominate from the same specimen of *Prophoca rousseaui* in lateral (C) and dorsal (D) view, with corresponding drawing of the lateral view (G); IRSNB M2234 left innominate of *Prophoca rousseaui* in lateral (E) and dorsal (F) view. The differences between IRSNB 1192-M276d and IRSNB M2234 have been attributed to sexual dimorphism.

**Innominate (Fig. 5)**: Three innominates are assigned to *Prophoca rousseaui*. Van Beneden (1877) assigned two associated innominates to *Prophoca rousseaui* (IRSNB 1192-M276b). These innominates have been found together with the sacrum IRSNB 1192-M276c and articulate perfectly with it. Hence, the sacrum and both innominates should be considered as belonging to the same individual. The third, isolated innominate IRSNB M2234 has been described by Louwye et al. (2010), but was identified as Phocidae aff. *Prophoca* based on minor morphological differences with the two aforementioned innominates; i.e., having a more slender anterior crest of the ilium and a mediolaterally more flattened anterior portion of the pubis and ischium.

In this paper, we tentatively assign this third, isolated innominate to *Prophoca rousseaui*. As Louwye et al. (2010) anticipated, we consider it very likely that the size and shape differences between this smaller innominate and the larger innominates IRSNB 1192-M276b may be attributed to sexual dimorphism. Sexual dimorphism and size differentiation is prominent among Phocidae (King, 1964). King (1964) also noted specific differences between male and female pinniped innominates, but these apply to the shape and size of the ischiac spine and the posterior portion of the ischium and pubis, which are neither preserved in IRSNB 1192-M276b nor in IRSNB M2234. Nevertheless, we consider the overall similarities between these innominates to outweigh the differences and tentatively consider IRSNB M2234 from Posthofbrug to represent *Prophoca rousseaui*. If so, the smaller, from Posthofbrug, would most likely represent a female and the larger a male, based on comparison with sexually dimorphic extant pinniped taxa (King, 1964). For all three innominates, only the ilium and the most anterior parts of the ischium and pubis are preserved.

In articulation with the sacral wing (at the auricular surface), the lateral surface of the innominate tends to face slightly dorsally. The same trait has been observed in the extant monachine *M. monachus*. The ilium is strongly elongate compared to most other Phocidae (see below). In general, Phocidae have a strongly reduced ilium compared to other carnivorans, which is presumably related to their aquatic locomotion, heavily relying on pelvic oscillations (e.g., Berta & Wyss, 1994; Bininda-Emonds & Russell, 1996). de Muizon (1981) considered the relatively less reduced ilium of the monachines *M. monachus* and *P. pacifica* to be plesiomorphic. The innominates of *Prophoca rousseaui* are incompletely preserved, precluding precise quantification of its shape. However, it appears that the ilium of *Prophoca rousseaui* is relatively elongated in relationship to the rest of the innominate, as compared to other Phocidae except *M. monachus* and *P. pacifica*. The ilium of *Prophoca rousseaui* has a markedly triangular shape (Van Beneden, 1877), having a pronounced iliac crest anteriorly and being dorsoventrally pinched posteriorly, as in phocines, contrasting with the roughly rectangular ilium of monachines (except the leopard seal, *H. leptonyx*, and the Ross seal, *O. rossii*) (de Muizon, 1981). The small lateral eversion of the ilium and the very shallow gluteal fossa in *Prophoca rousseaui* are shared with Monachinae and the basal phocines *E. barbatus* and *K. benegasorum*, and to a lesser extent with *C. cristata*. The anterior iliac crest is slightly laterally convex in *Prophoca rousseaui*, which has also been observed in the extant Phocinae, the extant monachines *L. weddellii*, *L. carcinophaga*, and *M. monachus*, and the extinct monachines *H. capensis* and *P. pacifica*, but not in the extant monachines *H. leptonyx, O. rossii* and the extinct monachine *A. longirostris*.

The posteroventral process of the ilium is well developed, as in other Phocinae (except *C. cristata* and *K. benegasorum*), and much better developed than it is in Monachinae (except *Monachus* spp.). However, it is still much better developed in the larger IRSNB 1192-M276b than it is in the smaller IRSNB M2234 from Posthofbrug.

The iliopectineal eminence is well developed. Hence, the ventral margin of the ilium between the iliopectineal eminence and the posteroventral process of the ilium, corresponding to the area of origin for the *m. psoas major* and *m. quadratus lumborum*, is strongly concave.

On the lateral side of the ilium there is a well-developed fossa for the origin of the *m. rectus femoris*. This fossa is best developed in the smaller Posthofbrug specimen. As has been pointed out by de Muizon (1981), in Monachinae, the anteroventral and anterodorsal processes of the ilium are at the same anteroposterior level, giving a vertical aspect to the anterior margin, whereas the anteroventral process is anterior to the anterodorsal process in Phocinae, *Mirounga* Gray, 1827, *Monotherium delognii* Van Beneden, 1877, and *Prophoca rousseaui*.

The acetabulum is very deep, as in other Phocinae and *Monachus* spp., and located high dorsally on the lateral side of the innominate.

The pubis and ischium are only very fragmentarily preserved in the three innominates described here. On the dorsal margin of the ischium, just posterior to the level of the anterior margin of the obturator window, there is a strongly developed, highly raised and anteroposteriorly elongated ischiac tuberosity.

**Figure 6:**
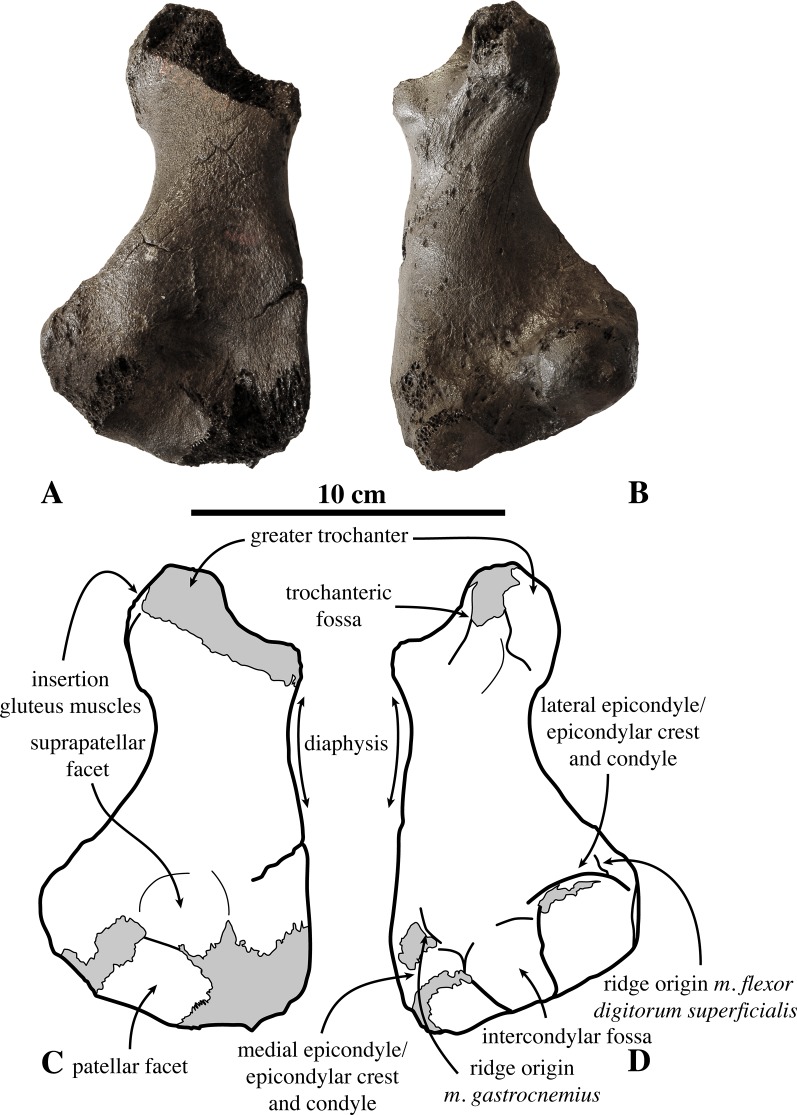
Femur of *Prophoca rousseaui*. IRSNB 1150-M277a right femur of *Prophoca rousseaui* (A, B) and corresponding drawings (C, D) in anterior (A, C) and posterior (B, D) view.

**Femur (Fig. 6)**: Within the material originally described by Van Beneden (1877), only one right femur is present (IRSNB 1150-M277a). This femur has been found in association with a partial tibia (IRSNB 1150-M277b). The capitulum is missing, as well as the anterodistal part of the medial epicondyle. A possible second femur only consists of a partial femoral capitulum. Louwye et al. (2010) tentatively referred this specimen to Phocidae aff. *Prophoca*, based on its stratigraphical occurrence and its size matching the innominates of *Prophoca rousseaui* IRSNB 1192-M276b. Based on the aforementioned arguments, we support this tentative designation from Louwye et al. (2010).

The femur attains a nearly triangular shape in anterior view, with the minimum width of the diaphysis located proximally and strongly broadening distally. A roughly similar triangular shape can be observed in extant Lobodontini. The stout greater trochanter is strongly rounded in anterior view. The proximal part of the greater trochanter being abraded, if complete a lip would most likely have overhung the trochanteric fossa, similar to other extinct and extant phocines. The anterolateral surface of the greater trochanter is heavily marked with muscle scars for the gluteus muscles. The trochanteric fossa is deep and slit-like. A deep trochanteric fossa is characteristic for Phocinae (King, 1956; de Muizon, 1981). Among monachines, only the extant *L. carcinophaga* and the extinct *C. obscura* show a similarly deep trochanteric fossa (de Muizon, 1981; Koretsky & Ray, 2008; Leonard Dewaele, 2016, personal observation).

Medially, the epicondylar crest is low, only weakly projecting, as in other Phocinae; but it is transversely thick and straight along its entire length. The lateral epicondyle is equally indistinct as the epicondylar crest, a feature shared with Monachinae (de Muizon, 1981). Distally, the transition between both the epicondylar crest and the lateral epicondyle and the diaphysis is marked by two low, proximodistally oriented ridges (one medial and one lateral). Such ridges are not found in extant phocids, but are presumed to correspond to the origin for powerful *m. gastrocnemius* and *m. flexor digitorum superficialis*.

The femur of *Prophoca rousseaui* has a much deeper and more clearly outlined suprapatellar fossa than extant Phocidae, similar to many other, extinct Phocinae, including *Phoca vitulinoides* (Leonard Dewaele, 2016, personal observation). This suprapatellar fossa is slightly smaller in size than the patellar facet.

The lateral margins of the patellar facet are not preserved, but the facet appears slightly higher than wide. This corresponds with the condition in Phocinae and the extinct *A. longirostris*, and points toward an increased mobility of the knee joint (de Muizon, 1981). Monachinae other than *A. longirostris* have a patellar facet that is wider than high, relating to a decreased mobility of the knee joint.

The intercondylar fossa is wide and deep. The medial condyle is smaller and less strongly curving than the lateral condyle. Although the medial condyle is not entirely preserved in IRSNB 1150-M277a, it is much smaller compared to the lateral condyle. Generally, the size difference between the lateral and medial epicondyles tends to be larger in Phocinae than in Monachinae (de Muizon, 1981).

**Figure 7:**
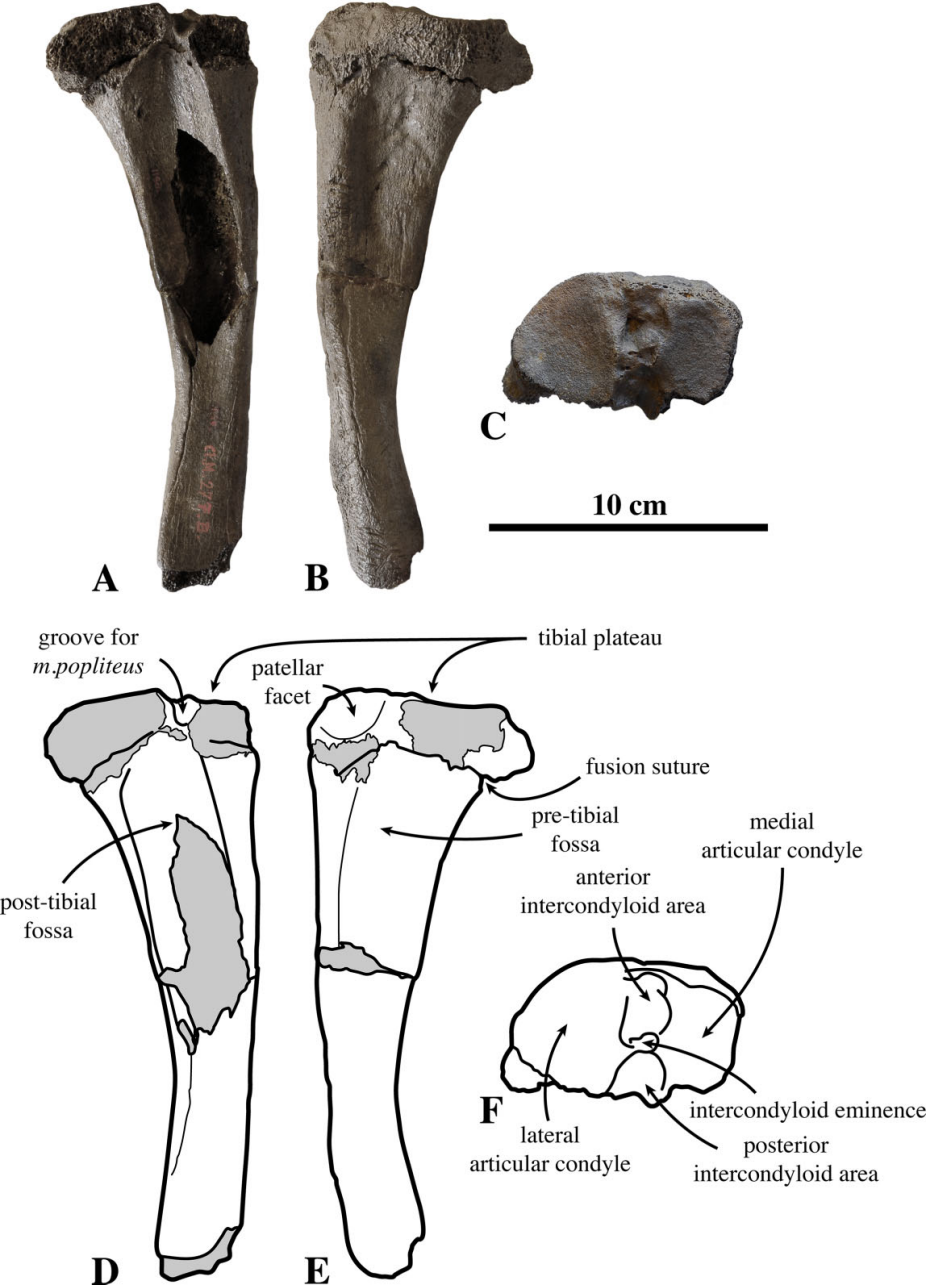
Tibia of *Prophoca rousseaui*. IRSNB 1150-M277b right tibia of *Prophoca rousseaui* (A–C) and corresponding drawings (D–F) in anterior (A, D), posterior (B, E) and proximal (C, F) view.

**Tibia (Fig. 7)**: The proximal portion of the tibia IRSNB 1150-M277b (associated to the femur IRSNB 1150-M277a) is assigned to *Prophoca rousseaui*. Van Beneden (1877) originally assigned a second left tibia (IRSNB-VERT-3250-15) to *Prophoca rousseaui* as well. However, for IRSNB-VERT-3250-15 only the proximal part of the tibia is preserved and only the little diagnostic diaphysis is visible. The tibial plateau is embedded in consolidated matrix. Hence, no morphological observations can be invoked to attribute the specimen to *Prophoca rousseaui* unambiguously, and IRSNB-VERT-3250-15 is only tentatively assigned to *Prophoca rousseaui*, on the basis of the overall similarity in size and morphology with the diaphysis of IRSNB 1150-M277b.

In IRSNB 1150-M277b, the suture between the diaphysis and the proximal epiphysis is visible, suggesting a skeletally almost adult specimen, based on observations in extant Phocinae (Storå, 2000). The tempo of fusion of the diaphyses and epiphyses of different bones varies within each taxon. Although the tempo of fusion of the diaphysis and epiphyses of different bones varies within each taxon, in phocine seals, the fusion of the diaphysis and proximal epiphysis of the tibia is one of the last steps to skeletal maturity (Storå, 2000).

The tibia only slightly curves laterally. This corresponds with Monachinae, in which the tibia generally curves less than it does in Phocinae (de Muizon, 1981). On the tibial plateau, anterior and posterior intercondyloid areas separate the articular condyles for the femur from each other. These intercondyloid areas are separated by the intercondyloid eminence. Both articular condyles are concave, yet the concavity of the medial condyle is faint. This difference may be due to the size difference between the two condyles, the lateral being considerably larger than the medial. The intercondyloid eminence is prominent and raised, similar to Phocinae and many extinct Monachinae such as *H. capensis*, *Monotherium aberratum* Van Beneden, 1877, and *P. pacifica*, (de Muizon, 1981; Leonard Dewaele, 2016, personal observation). The anterior intercondyloid area is well outlined in *Prophoca rousseaui* and the popliteal groove in the posterior intercondyloid area is deep. A well-outlined anterior intercondyloid area is a phocine character that has also been described for the monachine *H. capensis*, *Monotherium aberratum*, and *P. Pacifica* (and also for *Prophoca Rousseaui*) by de Muizon (1981).

Although poorly preserved, the patellar facet on the proximo-anterior margin of the tibia is shallow. In other Monachinae, this facet varies from low to high anterior to the main body of the tibia. The patellar facet is roughly semicircular in anterior view.

The anterior and posterior tibial fossae, for *m. tibialis cranialis* and *m. tibialis caudalis* respectively, are both moderately excavated.

#### Axial skeleton

**Figure 8:**
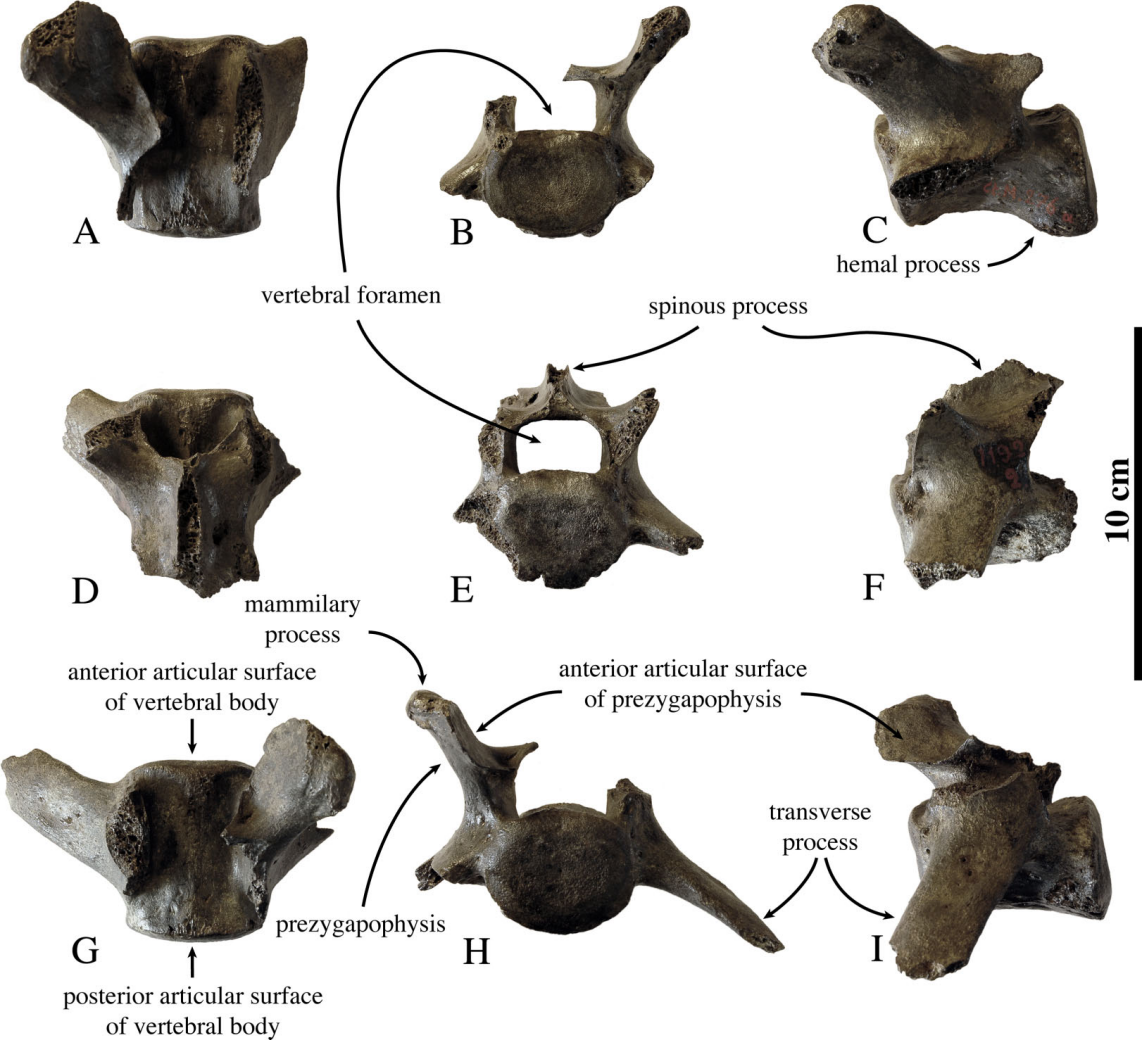
Lumbar vertebrae of *Prophoca rousseaui*. IRSNB 1192-M276a lumbar vertebrae of *Prophoca rousseaui* of uncertain positioning (LV1-LV4?) (A–F) and the fifth lumbar vertebra (G–I) in dorsal (A, D, G), anterior (B, E, H) and left lateral (C, F, I) view.

**Lumbar vertebrae (Fig. 8)**; Currently, three lumbar vertebrae IRSNB 1192-M276a have been assigned to *Prophoca rousseaui*. These vertebrae were found articulated with a sacrum and two innominata (IRSNB 1192-M276b-d). None of the three lumbar vertebrae is preserved (sub-) completely, but combined they give a good representation of the lumbar vertebrae of *Prophoca rousseaui*.

Based on the slightly reduced length of one vertebral body (54.6 mm) compared to the other vertebra with its body completely preserved (62.1 mm), the shorter one can tentatively be identified as the fifth, and last, lumbar vertebra (L5). Moreover, the shorter lumbar vertebra of *Prophoca rousseaui* lacks the two hemal processes, caudal on the ventral surface of the vertebral body, which is a general feature in Phocidae. The hemal processes are clearly present in the other completely preserved vertebral body. The two other vertebrae are more difficult to locate precisely. Not only does the mediocre state of preservation of the lumbar vertebrae inhibit to be conclusive on the subject, overall little variation between L1 and L4 in specimens of extant seals renders it difficult to extrapolate from them and pinpoint the exact position of individual lumbar vertebrae in extinct Phocidae.

The lumbar vertebrae of *Prophoca rousseaui* are anteroposteriorly elongated. The anterior and posterior articular surfaces are oval in L5 and reniform in the other two vertebrae.

The transverse processes are not preserved completely in any of the three vertebrae, but they appear to be robust, thick, and projecting anterolaterally. Although incomplete, the transverse processes in L5 of *Prophoca rousseaui* are proportionally relatively long, intermediate to the generally relatively short lumbar transverse processes of extant Monachinae and the relatively long lumbar transverse processes of extant Phocinae (Leonard Dewaele, 2016, personal observation).

The vertebral foramen is rectangular in shape and wider than it is high in all three specimens. We observed a similar state in the phocine harp seal *Pagophilus groenlandicus*, and ringed seal *Pusa hispida*, and in the monachine Mediterranean monk seal, *M. monachus*. In other extant Phocidae the lumbar vertebral foramina are semicircular or faintly dorsally pointed.

The lumbar prezygapophysis in *Prophoca rousseaui* is large and robust, compared to extant Phocidae. Also contrasting to extant Phocidae, the teardrop-shaped articular surface of the prezygapophysis is relatively large in size in *Prophoca rousseaui*. As a consequence, the blunt mammillary process is relatively small compared to the anterior articular surface in *Prophoca rousseaui*. The mammillary process is slightly offset posteriorly to the center of the anterior articular surface.

**Figure 9:**
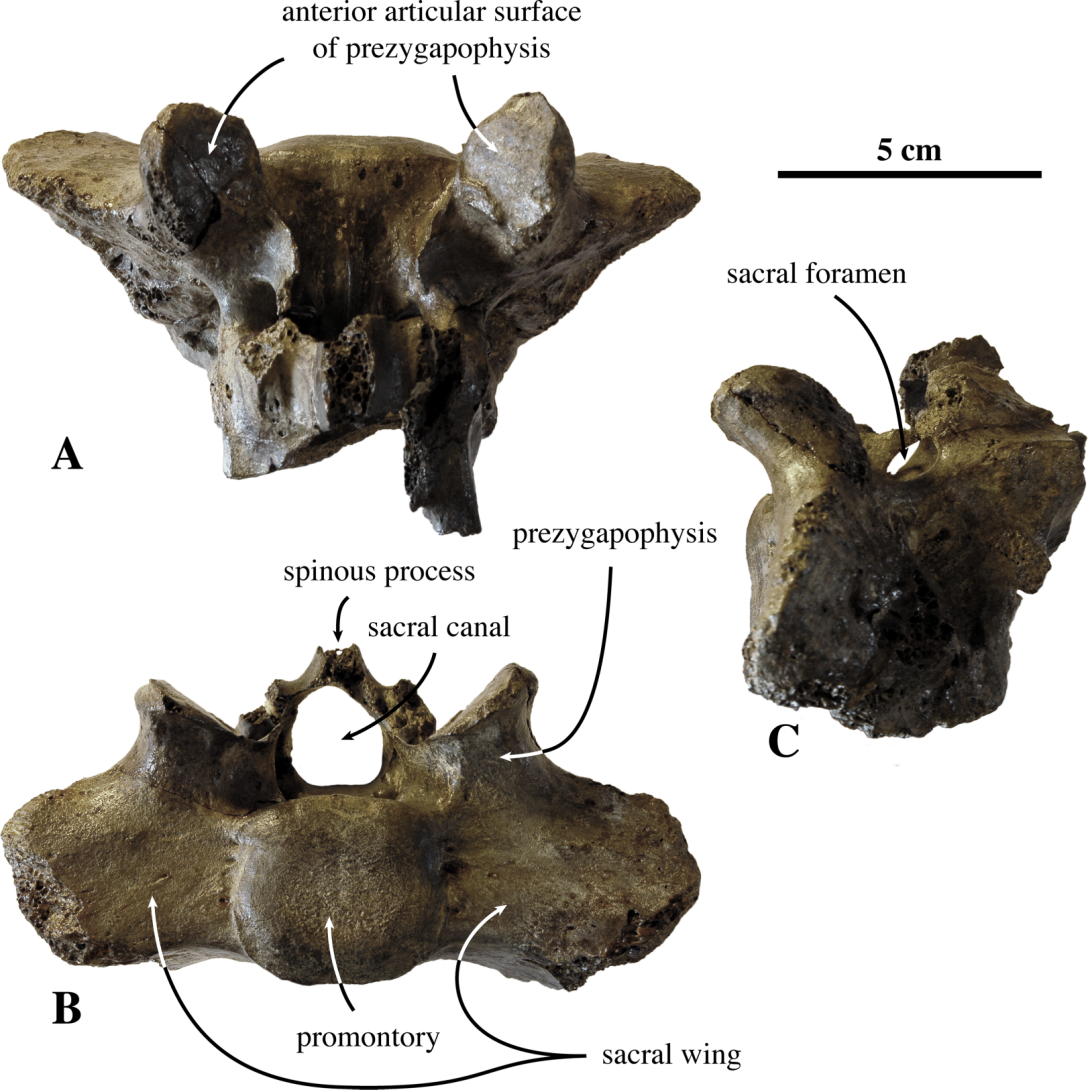
Sacrum of *Prophoca rousseaui*. IRSNB 1192-M276c sacrum of *Prophoca rousseaui* in dorsal (A), anterior (B) and left lateral (C) view.

**Sacral vertebrae (Fig. 9)**: Of the original material assigned to *Prophoca rousseaui*, one sacrum is present, with only the first sacral vertebra and the anterior part of the second sacral vertebrae preserved (IRSNB 1192-M276c). The preserved parts are poorly preserved, i.e., fragile processes and other elements are broken off, precluding a detailed description.

**Figure 10:**
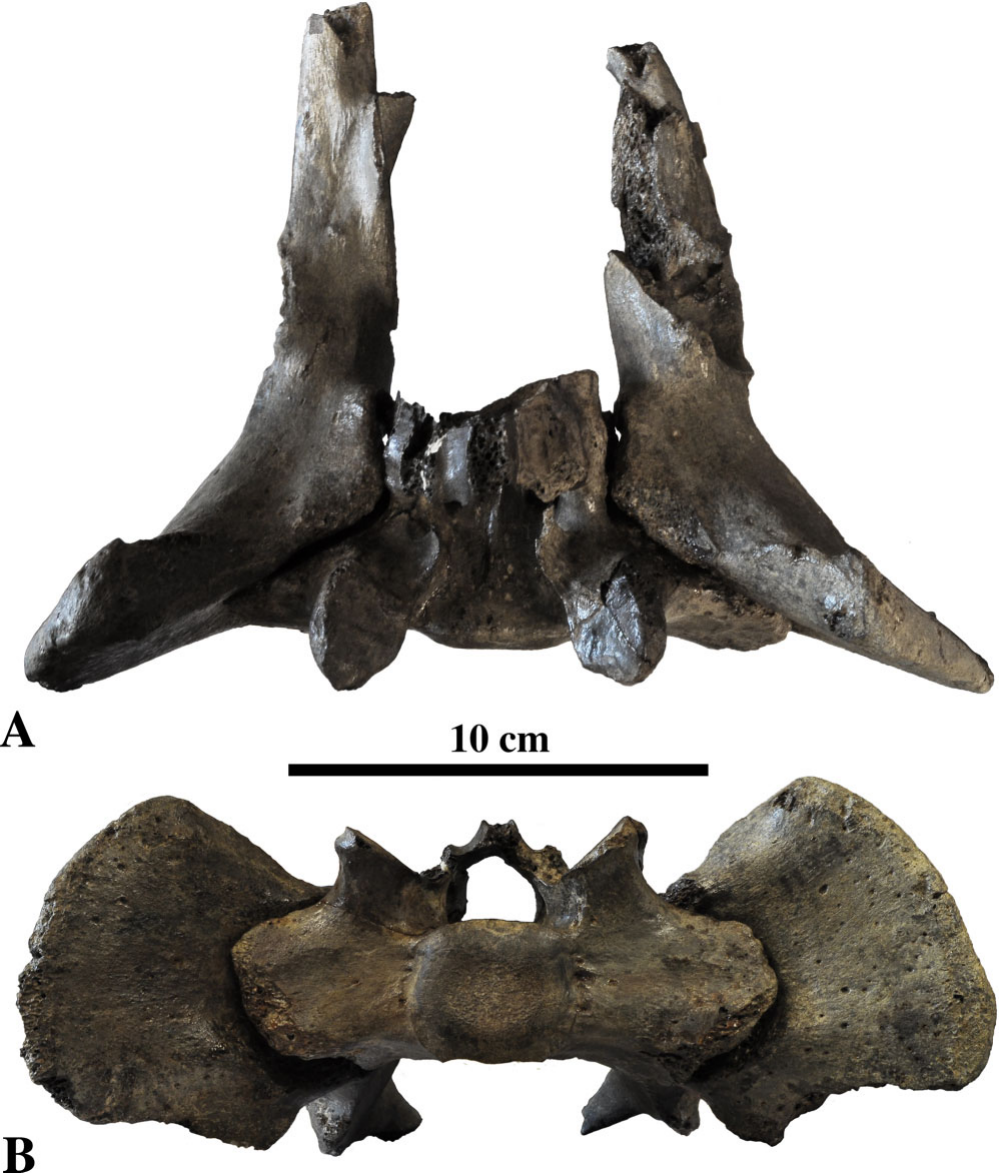
Pelvis of *Prophoca rousseaui*. Sacrum IRSNB 1192-M276c in articulation with innominates IRSNB 1192-M276b and d in dorsal (A) and anterior (B) view. The width across the sacral wings equals 3.31 times the width across the promontory. A similar high ratio has only been observed in certain extinct species of Phocidae (Supplemental Information 3).

The sacral wings (*alae*) are strongly laterally enlarged in *Prophoca rousseaui*, with a transverse width across the wings 3.31 times the lateral width across the promontory (131.5 mm/39.7 mm) (Fig. 10). In general, the lateral expansion of the sacral wings is considered as a synapomorphy distinguishing Phocidae from other Carnivora and among Phocidae these wings were considered more expanded in Monachinae than in Phocinae (de Muizon, 1981). However, the range of ratios observed in both living and fossil Phocinae and Monachinae (Supplemental Information 3) prevents clear distinction between the two subfamilies. The lowest ratio observed in our dataset of 56 specimens of 14 extant and extinct phocid species) (63.5:29.0 mm; ratio 2.19) is attributed to a specimen of the phocine harbor seal *Phoca vitulina*, while the highest ratio (147.2:41.7 mm; ratio 3.53) is attributed to a specimen of the fossil monachine *C. obscura*. The dataset shows intraspecific variability of the ratios, generally ranging between 10 and 40%, with the largest differences between highest and lowest ratios observed in *Phoca vitulina* (38.7%) and *C. obscura* (35.2%). Given the limited number of specimens included and because the sex of many specimens measured during this study remains unknown, no statistically supported differentiation between male and female could be established. Actual intraspecific variability is most likely higher than appears from the dataset (Supplemental Information 3). Notwithstanding, the sacral width ratio of 3.31 observed in the single sacrum attributed to *Prophoca rousseaui* is at the upper limit of the entire range observed (see Supplemental Information 3). Although the value of 3.31 for *Prophoca rousseaui* exceeds all observed extant phocids, but larger ratios have been observed in certain specimens of extinct phocids, i.e., *C. obscura*, *Phoca vitulinoides*, and *Phocanella pumila* Van Beneden, 1877.

Published studies on the musculature of pinnipeds are scarce (e.g., Bryden, 1971; Piérard, 1971; English, 1977), but de Muizon (1981) proposed that the *m. erector spinae* originated on the anterior surfaces of the sacral wings of *A. longirostris*. Contrastingly, Howell (1929) found that the *m. erector spinae* originated on the anterior border of the iliac crest in the ringed seal, *Pusa hispida* (*Phoca hispida*) (Schreber, 1775). However, Howell (1929) noted the strong development of the *erector spinae* muscles in *Pusa hispida* compared to the Californian sea lion, *Zalophus californianus* the latter having much smaller sacral wings; and King (1964) observed the strong development of *m. iliocostalis lumborum*, grouped within the *erector spinae* muscles, in phocids. Hence, the development of the sacral wings may tentatively be linked to the development of the *m. erector spinae* and the ability to extend and rotate the dorsum.

The lateral margins of the sacral wings are not preserved, hampering proper description. Yet, based on preserved parts, the reconstruction of the lateral outline of the wings appears convex laterally. This differs from extant Phocinae and Monachinae in which the lateral margins being straight and are divergent ventrally (Phocinae) or vertical (Monachinae) (or even ventrally convergent in *A. longirostris*). The wings are slightly offset dorsally, relative to the promontory, and project laterally, i.e., they do not project ventrolaterally as in other Phocidae.

The prezygapophysis has a thick base on the sacral wing. The anterior articular surface of the prezygapophysis is large, with a rounded triangular outline. Right and left articular facets are perpendicular to each other. The mammillary processes are virtually absent, as in many Phocidae (except the phocines *P. groenlandicus* and *Pusa* spp. and the monachine *L. carcinophaga*).

The promontory is slightly compressed dorsoventrally in anterior view. The sacral canal is subtriangular in section, though with strongly rounded corners. Similar to extant Phocinae, the pelvic sacral foramina are relatively small compared to extant Monachinae; the spinous process has a mediolaterally thick base.

*Leptophoca* True, 1906

**Type and only included species—***Leptophoca proxima* (Van Beneden, 1877), by subsequent designation (this work).
**Diagnosis—**Same as for the only included species.
***Leptophoca proxima* (Van Beneden, 1877) n. comb.**
*Prophoca proxima* Van Beneden, 1877: 802, Plate XVIII.
*Leptophoca lenis* True, 1906: 836–840, 2 plates.
*Leptophoca proxima* Ray, 1976a: 391–406.

**Lectotype:** Right humerus, IRSNB 1146-M279; illustrated and described by Van Beneden (1877, Plate XVIII, Figs. 12–14). Selected by Koretsky (2001) as lectotype of *Prophoca proxima*.

**Type Locality:** ‘Borgerhout, third section,’ Antwerp, Belgium. This section of the former fortification ring around the city of Antwerp runs from the district of Deurne to the former ‘porte de Borsbeek.’ The district of Borgerhout encompasses the northern portion of this section (approximately 51°12′47″N, 4°26′49″E Web Mercator).

**Type Horizon and Age:** ‘Anversien’ (Van Beneden, 1876, 1877). The precise location and stratigraphical position is unknown. Deméré, Berta & Adams (2003) reassigned the species to the Berchem Formation without providing any evidence. Dinoflagellate cyst biostratigraphic analysis of a sediment sample from the lectotype humerus IRSNB 1146-M279 of *Leptophoca proxima* provides a late Langhian–early Serravallian age (14.8–13.2 Ma, middle Miocene). As for *Prophoca rousseaui*, the highly diachronous nature of the Neogene strata from Belgium precludes assigning the lectotype specimen of *Leptophoca proxima* to a specific lithostratigraphic unit through biostratigraphic age determination. Also, the sediment sample from which the dinoflagellate cysts were recovered was too small to allow any comprehensive sedimentological analysis. Although a generally poor criterion, the dark color of the specimens corresponds rather to the gray–black Antwerpen Sands Member of the Berchem Formation than to the overlying, more brownish Deurne Sands from the Diest Formation. This does not contradict the results of the biostratigraphic analysis and would correspond to the stratigraphic position of *Prophoca proxima* as proposed by Deméré, Berta & Adams (2003).

**Comments:** Differing from the proposal of Ray (1976a) to reassign *Prophoca proxima* to the genus *Leptophoca*, but to retain *Leptophoca lenis* as a separate species, in the current study we synonymize *Prophoca proxima* and *Leptophoca lenis*, and propose the new combination *Leptophoca proxima*. Although the genus name *Prophoca* has age priority over *Leptophoca*, the distinct species *Prophoca rousseaui* has been named type species of the genus by Kellogg (1922) and has priority over *Prophoca proxima*. Following article 23 of the ICZN, synonyms *Prophoca proxima* and *Leptophoca lenis* are combined into *Leptophoca proxima* n. comb.

**Emended Diagnosis**: Large phocine, similar in size to large *E. barbatus*. The humerus of *Leptophoca proxima* differs from all other phocines in the following unique combination of characters: lesser tubercle of humerus small not reaching the proximal level of the humeral capitulum (also present in *Praepusa vindobonensis*, *Prophoca rousseaui* and *S. sintsovi*); intertubercular groove wide and shallow (also present in *C. cristata*, *E. barbatus* and *Prophoca rousseaui*); relatively straight posterior margin of the humeral capitulum; deltopectoral crest extending along the proximal two-third of humerus (also present in *C. maeotica*, *Phoca vitulinoides*, *Prae. vindobonensis*, *Prophoca rousseaui* and *S. sintsovi*); deltopectoral crest terminating abruptly, distally, but less abrupt than in extant Phocinae (also present in *Prophoca rousseaui*); deltopectoral crest mediolaterally thin; lateral epicondyle thin and strongly projecting posteriorly; deep and well-outlined coronoid fossa (also present in *Phoca* spp. and *Pusa* spp.)

Koretsky (2001) presented a diagnosis of the cranium and mandible of *Leptophoca proxima* (as *Leptophoca lenis*). However, this diagnosis is based on isolated skulls and skull fragments and mandibles. Without any supported association to *Leptophoca proxima*, i.e., association with the humerus, the designation of any cranial or mandibular specimen to *Leptophoca proxima* remains doubtful. Therefore, we acknowledge the diagnosis by Koretsky (2001), but neither accept nor reject it. Similarly, Koretsky (2001) tentatively assigned a significant number of isolated postcranial bones to *Leptophoca proxima*. However, given the abundance of humeri from the Calvert Formation and other formations of the Chesapeake Group (Neogene of the mid-Atlantic coastal plain, Delaware, Maryland, North Carolina, and Virginia) assigned to *Leptophoca proxima*, it can relatively safely be assumed that other phocine bones that have been found in relatively large numbers in the Chesapeake Group, such as femora and tibiae, can be related to *Leptophoca proxima* as well. Nevertheless, no femora or tibiae from the Neogene of Belgium can be assigned to *Leptophoca proxima*. Hence, because the current study focuses on material from Belgium, neither the femur nor the tibia of *Leptophoca proxima* will be treated in detail here.

**Referred specimens**: IRSNB 1146-M279, lectotype, left humerus, section no. 3 at Borgerhout, Antwerp, Belgium, Miocene, ‘Anversien’; USNM 5359, right humerus, holotype *Leptophoca lenis*, Miocene, latest Burdigalian–Langhian, Calvert Formation, Shattuck zone 10, float from zone 10 at Plum Point, Calvert County, Maryland, USA; USNM 23450, left humerus, Miocene, late Langhian–early Serravalian, Calvert Formation, Plum Point Member, Shattuck zone 12, second hill south of Parkers Creek, approximately 30 cm above beach level, Calvert County; USNM 186990, right humerus, Miocene, middle Serravalian–late Serravalian, Choptank Formation, Shattuck zones 18–20, approximately 6 km NW of Cove Point, Calvert County; USNM 284721, right humerus, Miocene, late Langhian–early Serravalian, Calvert Formation, Plum Point Member, Shattuck zone 12, Pope’s Creek at Stratford Bluffs, Westmoreland County, Virginia; USNM 412115, left humerus, Miocene, Calvert Formation (Plum Point Member) or base of Choptank Formation, Shattuck zones 13–16, Scientists Cliffs, Calvert County, Maryland, US; IRSNB 1145-M280a-b, left ulna and left radius, fort no. 3 at Borsbeek, Antwerp, Miocene, ‘Anversien’; USNM 263648, right radius, Miocene, Calvert Formation (Plum Point Member) or base of Choptank Formation, Shattuck zones 11–16, north of Scientists Cliffs, Calvert County. The geological ages for specimens of North America listed above are deduced from associating the known geographic locations to stratigraphic charts and logs of locations from Kidwell et al. (2015).

#### Description

**Figure 11:**
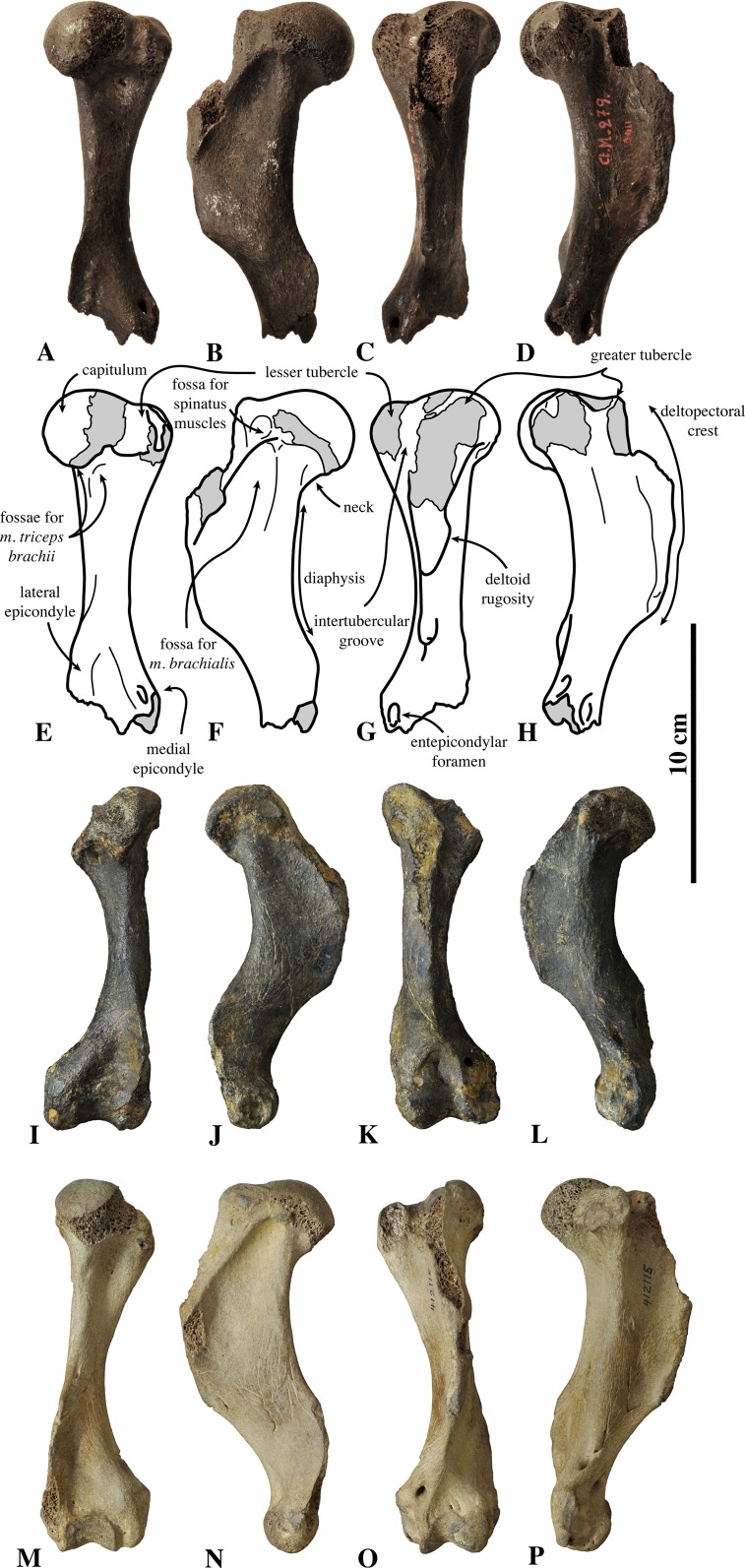
Humerus of *Leptophoca proxima*. IRSNB 1146-M279 left lectotype humerus (A–H), USNM 5359 right humerus (I–L) and USNM 412115 left humerus (M–P) of *Leptophoca proxima* in posterior (A, E, I, M), lateral (B, F, J, N), anterior (C, G, K, O) and medial (D, H, L, M) view.

**Humerus (Fig. 11)**: The lectotype left humerus of *Leptophoca proxima*, IRSNB 1146-M279, is incomplete, missing the proximal portion of the deltoid crest and the distal epiphysis. It is larger than the original holotype of *Leptophoca lenis* (USNM 5359), as noted by True (1906) (Supplemental Information 1).

The capitulum is located only slightly proximal to the greater tubercle. But both the capitulum and the greater tubercle reach much more proximal than the lesser tubercle. The round, knob-like lesser tubercle is small, especially compared to extant Phocidae. Many other extinct Phocinae also have a lesser tubercle smaller than in extant Phocinae, but they are still more prominent and reaching higher proximal than in *Leptophoca proxima* (see Koretsky, 2001; Leonard Dewaele, 2016, personal observation). The posterior surface of the lesser tubercle bears clear scars for the attachment of the *m. subscapularis*. Koretsky (2001), similarly considers that the lesser tubercle of *Leptophoca lenis* is located distal to the capitulum and the greater tubercle, but also that both the greater and lesser tubercle are located distal to the capitulum in *Prophoca proxima*. However, we tend to disagree with the latter, based on the incompleteness of the single humerus formerly attributed to *Prophoca proxima*, which has a little damaged lesser tubercle but a strongly damaged greater tubercle. In IRSNB 1146-M279, the proximal part of the greater tubercle is indeed missing. If complete, its greater tubercle would approximately reach the same level as the capitulum and reach much more proximal than the lesser tubercle, as is the case in specimens attributed to *Leptophoca lenis*.

The intertubercular groove is very poorly preserved in the lectotype, but it is narrow and appears to be deep in better-preserved specimens (USNM 5359 and USNM 412115). However, the latter could not be ascertained due to the general poor preservation of the greater tubercle.

The deltopectoral crest is widest proximally and becomes very thin, blade-like, and distally. This has previously been observed for specimens attributed to *Leptophoca lenis* (True, 1906; Koretsky, 2001) and to *Prophoca proxima* (True, 1906). The deltopectoral crest extends presumably for approximately two-thirds of the length of the bone. According to True (1906), the deltopectoral crest extends beyond half-length of the bone in both *Leptophoca lenis* and *Prophoca proxima*, while Koretsky (2001) makes the distinction between *Leptophoca lenis* and *Prophoca proxima*, stating that the deltopectoral crest extends for more than two-thirds of the total length of the humerus in *Prophoca proxima* and less than two-thirds in *Leptophoca lenis*. Given the incompleteness of the humeri attributed to both species, we do not follow Koretsky’s separation of both species on the basis of this character. In extant Phocinae, the deltopectoral crest is generally restricted to the proximal half of the humerus and never attains a prominently blade-like shape as it does in *Leptophoca proxima*. However, Monachinae and certain fossil Phocinae, such as *K. benegasorum*, *M. pontica* and *S. sintsovi* (Cozzuol, 2001; Koretsky, 2001), have deltopectoral crests longer than half the length of the humerus. The medial side of the deltopectoral crest is straight; on the lateral side of the crest the deltoid tuberosity extends along the proximal half of the crest, as observed by Koretsky (2001) in specimens referred to *Leptophoca lenis* and *Prophoca proxima*. Proximal on the lateral surface of the deltopectoral crest, there is a small but clearly visible round and shallow fossa for the spinatus muscles.

In posterior view, the diaphysis of *Leptophoca proxima* is much thinner than in other Phocidae. True (1906) already noted this character in the holotype specimen of *Leptophoca lenis* (and named the species accordingly: *leptòs* means “thin” or “slender” in Greek) and in the humerus of *Prophoca proxima*. Overall, the diaphysis of *Leptophoca proxima* is straight. True (1906) noted the straighter diaphysis (shaft) in *Prophoca proxima* compared to *Leptophoca lenis*. This is indeed true when comparing the lectotype humerus of *Prophoca proxima* (IRSNB 1146-M279) with the holotype humerus of *Leptophoca lenis* (USNM 5359) and other, more recently found specimens (e.g., USNM 186990 and USNM 284721). However, other recently found humeri (e.g., USNM 23450 and USNM 412115) also have a straighter diaphysis than the holotype of *Leptophoca lenis*. Ray (1976a) pointed out that a relatively straight humerus is a character state intermediate between terrestrial carnivorans and early Pinnipedimorpha (Berta, Ray & Wyss, 1989) on the one hand, and later-branching Phocidae on the other hand. Pinnipedimorpha are a monophyletic group including *Enaliarctos* Mitchell & Tedford, 1973 and the Pinnipediformes Berta, 1994, the latter including *Pteronarctos* Barnes, 1989, the three modern pinniped families: Odobenidae Allen, 1880, Otariidae and Phocidae, and the two extinct pinniped families Desmatophocidae Hay, 1930 and Semantoridae Orlov, 1933. In other Phocinae, we only noted a relatively straight humeral diaphysis in *C. cristata*. On the diaphysis, there are two distinct fossae for muscle origins. On the posterior surface, just distal to the capitulum and the lesser tubercle, there is a distinct fossa for the *m. triceps brachii caput mediale*. The surface for the *m. triceps brachii caput laterale* just distal and lateral to the capitulum is clearly marked as well. The fossa proximal on the lateral surface of the diaphysis and the deltopectoral crest serves as the origin of *m. brachialis*. Especially the cranial portion of this fossa is very deep, compared to other fossil and living Phocidae.

The lateral epicondyle is well developed but thin and strongly projecting posteriorly. The lateral epicondyle of *Leptophoca proxima* is thinner than in other Phocinae. Proximally, the lateral epicondyle reaches the same level as the distal part of the deltopectoral crest, as has also previously been observed for specimens previously attributed to *Prophoca proxima* and *Leptophoca lenis* (Koretsky, 2001). The medial epicondyle is not preserved on the lectotype of *Leptophoca proxima*, but it is preserved in some North American specimens of that species, where it is not particularly strongly developed (e.g., USNM 5359, USNM 186990, USNM 284721, and USNM 412115). We follow Koretsky’s description (for *Leptophoca lenis*) that the medial epicondyle is anteroposteriorly compressed and not extending proximal to the coronoid fossa. The entepicondylar foramen is rather small (*contra* Koretsky, 2001; for *Prophoca proxima* and *Leptophoca lenis*) and oval, with a wide bridge over it.

Neither the olecranon fossa nor the coronoid fossa is preserved in the lectotype, but it is in USNM 5359, USNM 186990, USNM 284721, and USNM 412115 from North America. The olecranon fossa of *Leptophoca proxima* is wide and shallow, and the coronoid fossa is clearly outlined, subtriangular and deep, as already noted by Koretsky (2001) (for *Leptophoca lenis*).

Overall, the humerus of *Leptophoca proxima* shows similarities to the Histriophocini tribe and particularly to the living ribbon seal *H. fasciata*. In both *Leptophoca proxima* and the Histriophocini, the origin for the *m. brachialis* forms a well-developed fossa. *Leptophoca proxima* and *H. fasciata* both have a relatively straight humeral diaphysis.

**Figure 12:**
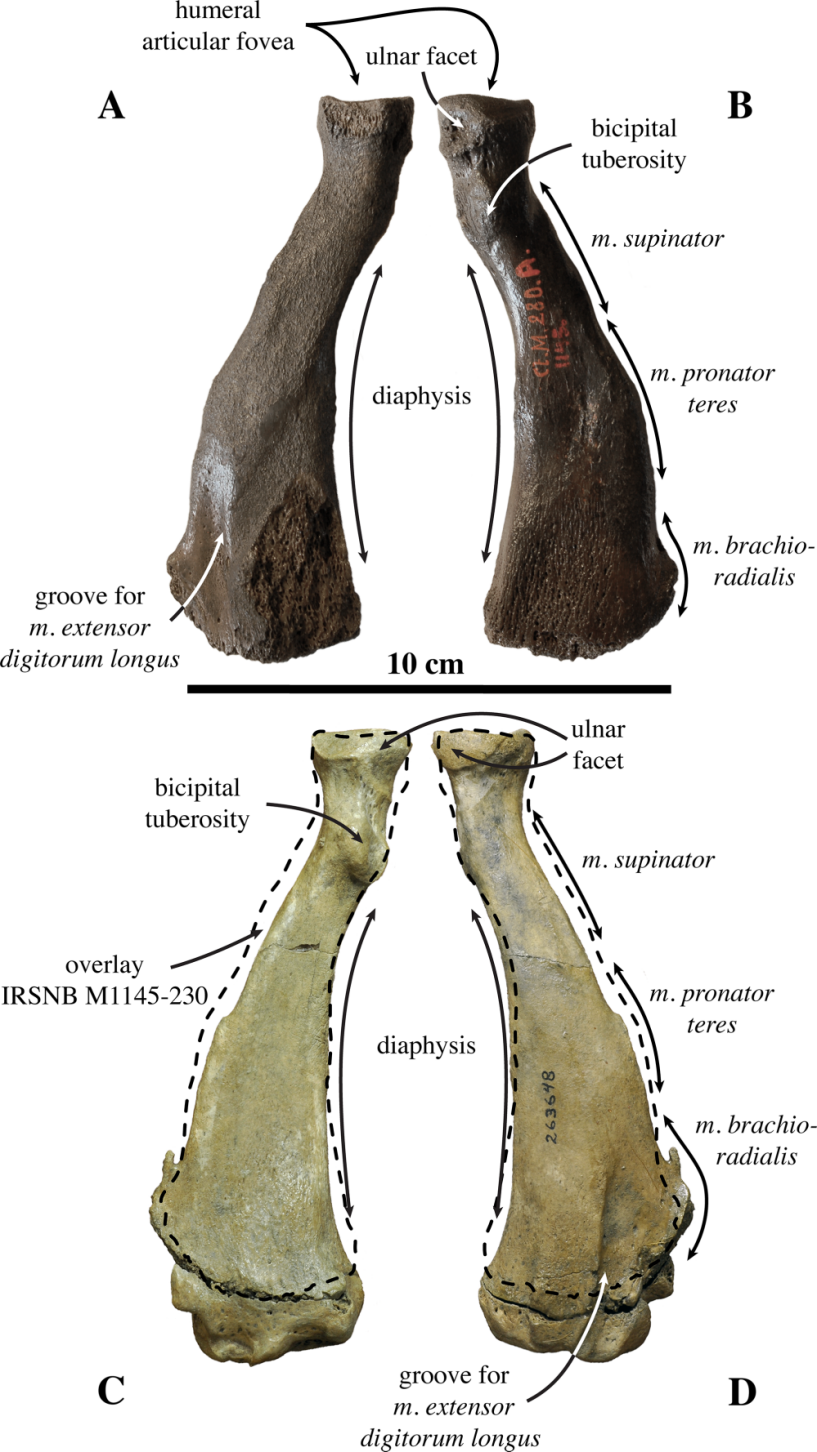
Radius of *Leptophoca proxima*. IRSNB 1145-M280b left radius (A, B) and USNM 263648 right radius (C, D) of *Leptophoca proxima* in lateral (A, D) and medial (B, C) view.

**Radius (Fig. 12)**: Only one left radius from the collection at the IRSNB has formerly been assigned to *Prophoca proxima* (IRSNB 1145-M280a), and, hence, currently *Leptophoca proxima*. This radius had been found in association with the partial ulna IRSNB 1145-M280b. Lacking its distal epiphysis, the radius is considered as a late subadult (see Storå, 2000). Without the distal epiphysis fused, the total length of the radius is 114.7 mm. Similarly, specimen USNM 263648 previously referred to *Leptophoca lenis* and here identified as *Leptophoca proxima* also represents a juvenile to subadult specimen. Both specimens are of the same size.

The radius is slender and strongly curving in the parasagittal plane, similar to other Phocinae (except *E. barbatus*) and unlike Monachinae. The radius USNM 263648 is slightly more slender than IRSNB 1145-M280a.

The ulnar facet of the humeral articular circumference is lens-shaped, arching around the posterior and medial margins of the articular fovea. A small triangular facet for articulation with the humeral trochlea is located anterior to the aforementioned ulnar facet on the medial side of the radius. Circumferentially, the humeral articular circumference is not as large compared to the proximal part of the diaphysis, than it tends to be in extant Phocidae.

The bicipital tuberosity is large and rounded, as Koretsky (2001) already noted, but slightly more flattened in the Belgian specimen. The bicipital tuberosity is located posteromedially on the diaphysis. At the level of the bicipital tuberosity, the diaphysis forms an angle. This corresponds with other Phocinae and contrasts to Monachinae, which generally have a more smoothly curving diaphysis at the level of the bicipital tuberosity.

de Muizon (1981) noted well developed insertion surfaces for *m. supinator* and *m. brachioradialis* and a weakly developed insertion surface for *m. pronator teres* on the diaphysis of the radius in all extant Phocidae (except Lobodontini showing the reverse condition), *P. pacifica*, and Otariidae. In *Leptophoca proxima* we observed an intermediate state: the insertion surface for *m. supinator* is moderately well developed, while on the other hand, the insertion surfaces for *m. pronator teres* and *m. brachioradialis* are relatively little developed. However, the surface for the insertion of the *m. brachioradialis* is located more distal on the anterior margin of the radius, as compared to modern Phocinae. It also protrudes more in *Leptophoca proxima* than it does in extant Phocinae. Our observations contrast with Koretsky (2001), stating that the insertion surface for *m. pronator teres* is almost absent in *Prophoca proxima*. We agree that the insertion surface for *m. pronator teres* is indeed slightly better developed in the North American specimen USNM 263648, but we consider the insertion surface for both still better developed than in Lobodontini. The ridge guiding the tendon groove of *m. extensor digitorum communis*, distal on the lateral side of the radius is very pronounced in comparison with extant and other extinct Phocinae, as noted by Koretsky (2001) for *Leptophoca lenis*.

**Figure 13:**
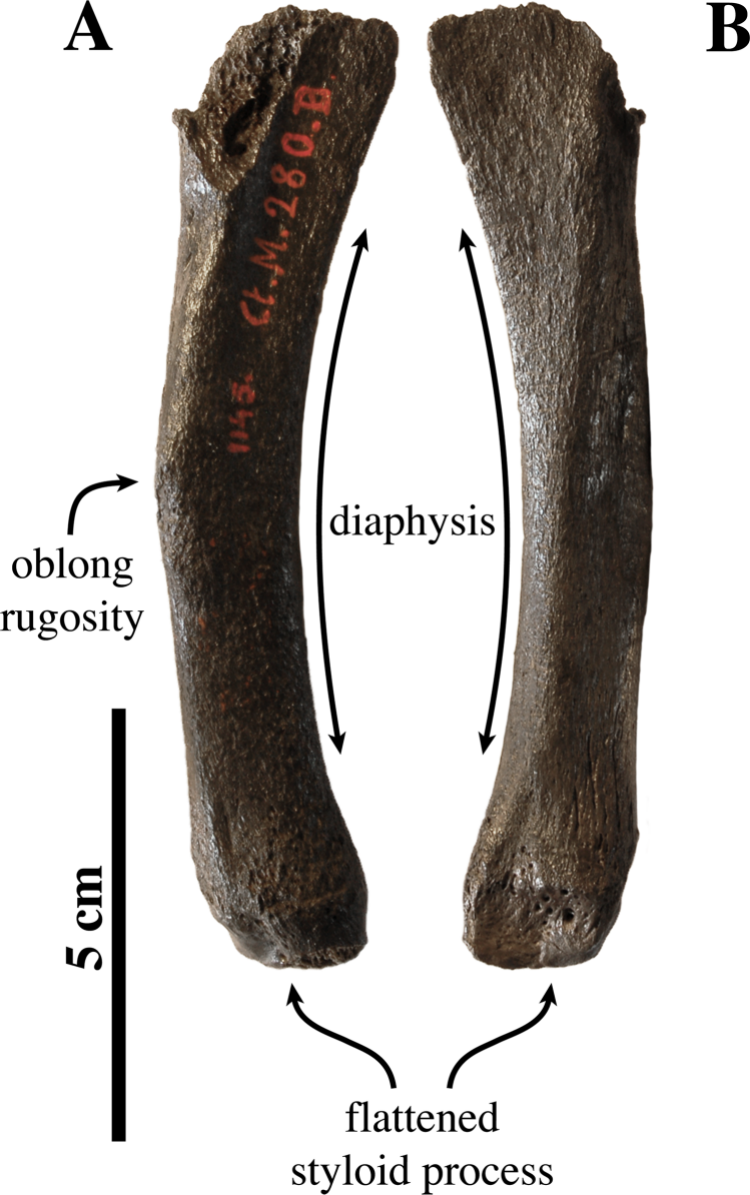
Ulna of *Leptophoca proxima*. IRSNB 1145-M280a left ulna of *Leptophoca proxima* in lateral (A) and medial (B) view.

**Ulna (Fig. 13)**: Only one ulna (IRSNB 1145-M280b) had been attributed to *Prophoca proxima* (Van Beneden, 1877); and is associated with the radius IRSNB 1145-M280a. Formerly, no ulna of *Leptophoca lenis* had been described (see Koretsky, 2001). Hence, this unique ulna has been assigned to *Leptophoca proxima* based on its association with a radius assigned to that species.

Only the diaphysis of the ulna is preserved. This portion is relatively large, pointing toward an estimated total length in the order of 15 cm when complete. The diaphysis is robust and the distal epiphysis is styloid, i.e., pointed. The oblong rugosity is faintly developed. This fragmentary bone does not differ significantly from the ulna of other Phocinae.

**Phocinae aff. *Leptophoca proxima***

Referred specimens—IRSNB M2233, right humerus, Miocene, Antwerp, Belgium; IRSNB M2232, right fibula, Miocene, Antwerp.

#### Description

**Figure 14:**
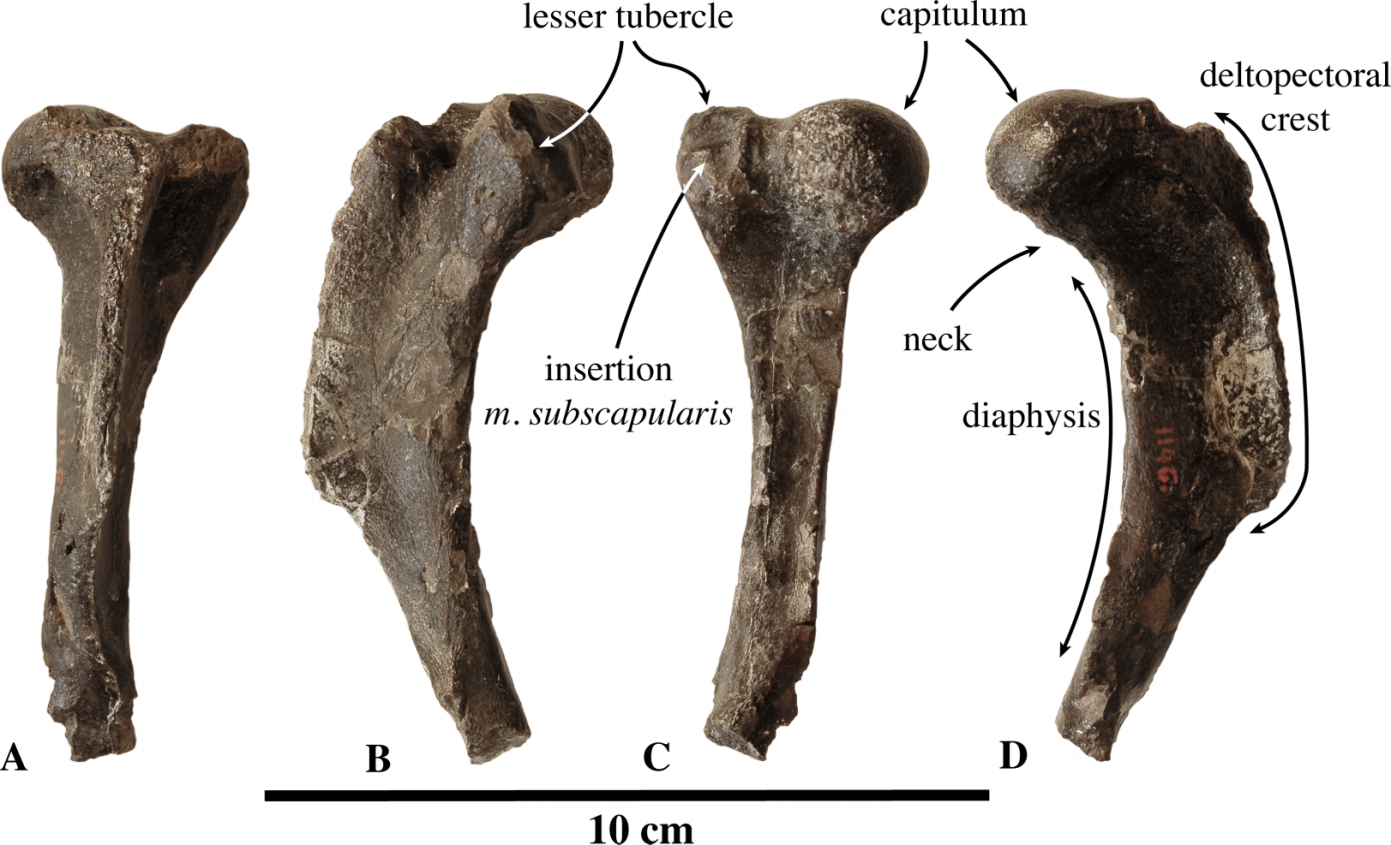
Humerus of Phocinae aff. *Leptophoca proxima*. IRSNB M2233 right humerus of Phocinae aff. *Leptophoca proxima* in anterior (A), medial (B), posterior (C) and lateral (D) view.

**Humerus (Fig. 14)**: One right humerus (IRSNB M2233), originally attributed to *Prophoca proxima* by Van Beneden (1877) is significantly smaller than the aforementioned *Leptophoca proxima* humerus IRSNB 1146-M279; it better matches the size of femora attributed to *Leptophoca amphiatlantica* (no humerus known for the latter) and the North American humeri of *Leptophoca proxima*. The humerus IRSNB M2233 misses its distal portion and is strongly abraded.

Although slightly abraded, the capitulum is roughly hemispherical and located high proximally. The capitulum is located slightly proximal to both the lesser and the greater tubercle and strongly projecting anteriorly. The round, knob-like lesser tubercle is small, but slightly more pronounced than in IRSNB 1146-M279 and USNM 412115, humeri identified as *Leptophoca proxima*. The posterior surface of the lesser tubercle bears clear scars for the attachment of the *m. subscapularis*. The intertubercular groove is narrow and it appears to be deep.

The deltopectoral crest is wider proximally and extends presumably for the proximal half of the length of the bone. The medial side of the deltopectoral crest is straight and the deltoid tuberosity extends on the lateral side of the crest along its proximal portion, before tapering abruptly distally. Along the distal two-thirds, the deltopectoral crest is blade-like and mediolaterally thin.

As in *Leptophoca proxima*, the diaphysis is thin compared to the proximal epiphysis: less than one third at its thinnest, in posterior view. However, the diaphysis of IRSNB M2233 is significantly thinner than in *Leptophoca proxima* in lateral view. The posterior surface of the diaphysis is relatively straight, as in *Leptophoca proxima*. On the diaphysis, just distal to the capitulum and the lesser tubercle the fossa for *m. triceps brachii caput mediale* is moderately well developed. Proximal on the lateral surface of the diaphysis and the deltopectoral crest, the origin of the *m. brachialis* is clearly outlined and well developed.

This humerus IRSNB M2233 shows some marked affinities with *Leptophoca proxima*, but differs on some noticeable aspects. Similarities include the small lesser tubercle; the thin deltopectoral crest, profoundly extending distally; the narrow diaphysis; and the strong anterior projection of the capitulum. Marked differences are the extreme thinness of the diaphysis and the concavity of the posterior surface of the diaphysis. Due to the absence of the distal portion and the strong abrasion of IRSNB M2233, it is difficult to ascertain whether the differences between IRSNB M2233 and *Leptophoca proxima* can be attributed to intraspecific variation (including sexual variation) or not.

**Fibula:** Van Beneden (1877) identified one very fragmentary right fibula (IRSNB M2232) as *Prophoca proxima*. Of this fibula, only the distal half is moderately well preserved. In its current condition, the fibula bears very little—if any—diagnostic information.

The fibula is short and transversely thin; based on the preserved parts it is comparable in size to the fibula of members of the genus *Pusa* Scopoli, 1777. It is slightly curving sigmoidally, but more than is usual for Phocidae. The diaphysis is triangular in cross-section, having approximately straight lateral and anterior surfaces. The posterior surface is deeply concave, representing a well-developed fossa for the origin of the *m. flexor hallucis longus*.

The distal epiphysis, i.e., the lateral malleolus, is square with clearly visible tendon grooves on its posterior surface. The distal articular surface for the astragalus is horizontal and only slightly concave. In extant Phocinae, the distal articular surface of the fibula is strongly sloping laterally and more strongly concave.

The size and general shape of IRSNB M2232 corresponds to that of specimen USNM 372547, found at the east coast of North America. However, the fibula is generally considered of very limited use to differentiate phocid species (Hodgetts, 1999). Combined with the poor state of preservation of IRSNB M2232, it is very difficult to unambiguously assign this specimen to *Leptophoca*.

**Comments on *Leptophoca amphiatlantica*** Koretsky, Ray & Peters, 2012

*Leptophoca amphiatlantica* was described only recently by Koretsky, Ray & Peters (2012). The species has been erected, based on four isolated partial femora: three specimens from the Calvert and St. Marys formations (lowermost Aquitanian to middle Tortonian) from the east coast of North America; and one specimen from the Breda Formation (uppermost Burdigalian to Langhian) from the Netherlands. However, we consider the evidence in support of *Leptophoca amphiatlantica* as a distinct species relatively weak.

At the time of the formal description of *Leptophoca amphiatlantica*, the only other known species in the genus was *Leptophoca lenis*. As mentioned above, the holotype of *Leptophoca lenis* is an isolated humerus, and more recently described specimens tentatively attributed to the species—including femora—have been found isolated (Koretsky, 2001). Therefore, the identification and diagnosis of the type femora of *Leptophoca amphiatlantica* in relation to *Leptophoca lenis* is precluded due to non-overlapping type specimens.

**Figure 15:**
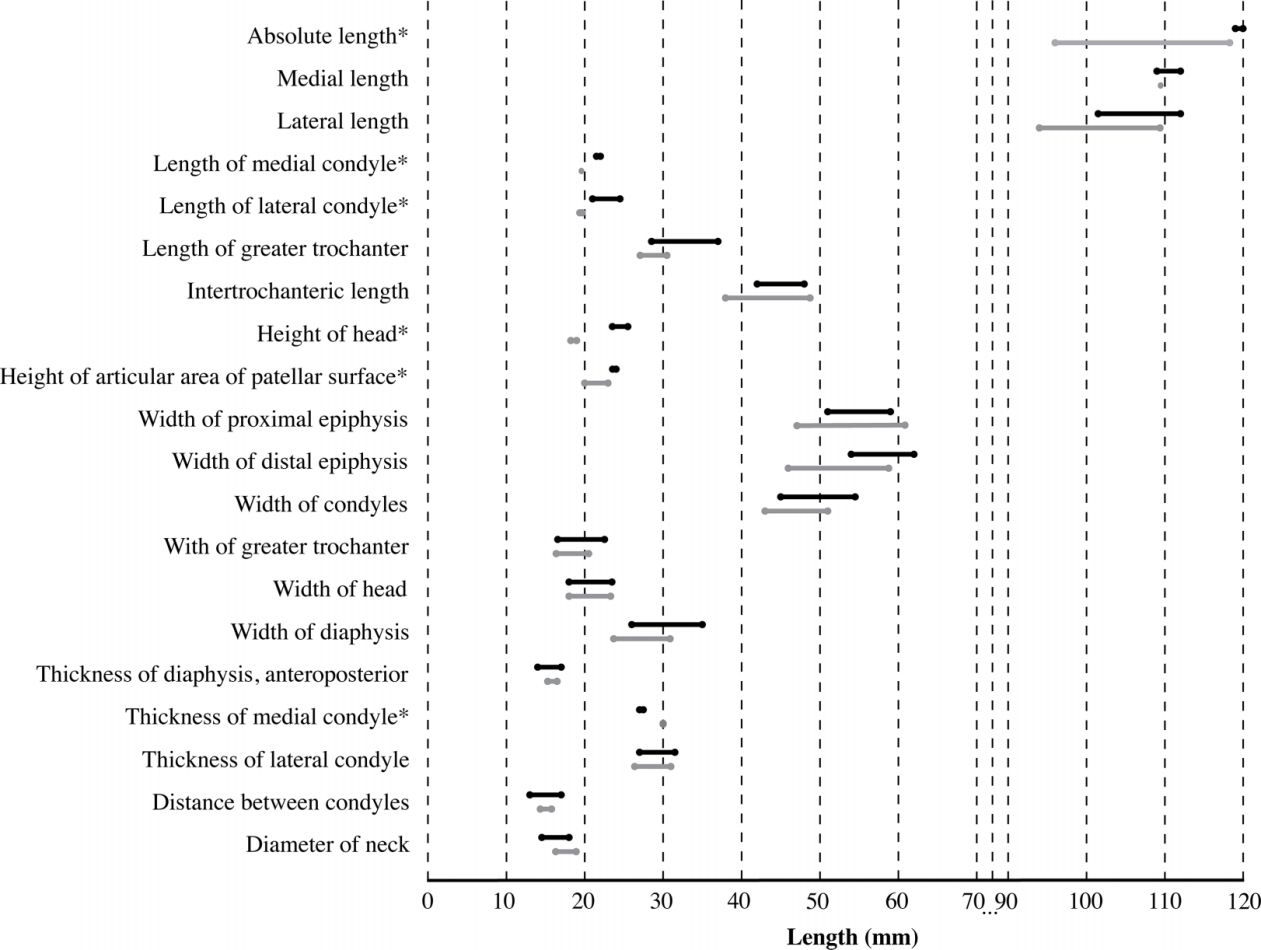
Biometric measurements of *Leptophoca proxima* and *Leptophoca amphiatlantica*. Graph comparing biometric measurements of *Leptophoca proxima* and *Leptophoca amphiatlantica*, adopted from measurements presented by Koretsky (2001; *L. proxima* as *Leptophoca lenis*) and Koretsky, Ray & Peters (2012; *Leptophoca amphiatlantica*). Measurement ranges in black (upper) for *Leptophoca proxima* and in gray (lower) for *Leptophoca amphiatlantica*. Characters with no overlap between both OTUs are indicated by an asterisk.

The described overall size discrepancy between *Leptophoca amphiatlantica* and *Leptophoca proxima* does not appear to be supported by measurements (Supplemental Information 1; Table 2; Fig. 15). When comparing measurements of both *Leptophoca* species, most measurements overlap between both species. Ranges do not overlap for only a few measurements: absolute length, length of the medial and lateral condyle, width of the medial condyle, height of the humeral head and height of the articular surface of the patellar surface. Because of the limited number of (often fragmentary) femora attributed to *Leptophoca amphiatlantica* and *Leptophoca lenis*, the statistical value of the biometric differences is very low. Indeed, given the often fragmentary nature of the specimens, not all characters could be measured for all specimens, and the numbers of measurements range from two to nine in *Leptophoca lenis* (Koretsky, 2001) and from one to four in *Leptophoca amphiatlantica* (Koretsky, Ray & Peters, 2012). For instance, although the absolute length range of *Leptophoca lenis* exceeds that of *Leptophoca amphiatlantica*, both ranges are based on only two specimens, and it can be suggested that the true intraspecific variation of *Leptophoca lenis* must have been larger than the 119.0–120.0 mm range presented (Koretsky, 2001; see also Supplemental Information 1; Table 2) and may, hence have overlapped with *Leptophoca amphiatlantica*. Also, two left femora of *Leptophoca amphiatlantica*, USNM 321926 and MAB 2129, bear clear signs of mechanical abrasion, further allowing questioning the value and validity of the presented measurements. Apart from the small number of measurements, the significance of non-overlapping measurement ranges can be questioned due to the nearness of the measurement ranges in both species. On an absolute length for the bone in the order of > 100.0 mm, the statistical significance of a condylar size difference in the order of a few millimeters, based on two specimens of *Leptophoca lenis* and one specimen of *Leptophoca amphiatlantica*, is questionable.

We support Koretsky, Ray & Peters (2012) in the observation of certain differences between the holotype of *Leptophoca amphiatlantica*, USNM 23227, and isolated femora attributed to *Leptophoca proxima*: i.e., the shape and position of the trochanteric fossa. Nonetheless, Koretsky (1987) observed sexual dimorphism at the level of the greater trochanter both in living *Pusa* spp. and in fossil *M. pontica*. Therefore, we question the validity of the differences at the level of the greater trochanter to separate *Leptophoca amphiatlantica* from *Leptophoca proxima*.

Pending the discovery of more complete/better preserved specimens for both *Leptophoca proxima* and *Leptophoca amphiatlantica*, we provisionally reassign the specimens originally attributed to *Leptophoca amphiatlantica* to *Leptophoca* cf. *Leptophoca proxima*, hereby confirming the presence of certain characters that differ from *Leptophoca proxima* but considering them too weak to define a distinct species; accordingly we consider the species name *Leptophoca amphiatlantica* a *nomen dubium*. Because the specimens of *Leptophoca amphiatlantica* cannot be unambiguously reassigned to *Leptophoca proxima*, we cannot consider *Leptophoca amphiatlantica* to be a proper junior synonym to *Leptophoca proxima*.

### Phylogenetic analysis

The aim of this phylogenetic study is to trace the phylogenetic position of *Prophoca rousseaui* and *Leptophoca proxima* in relation to other Phocidae (see also Supplemental Informations 4 and 5).

**Figure 16:**
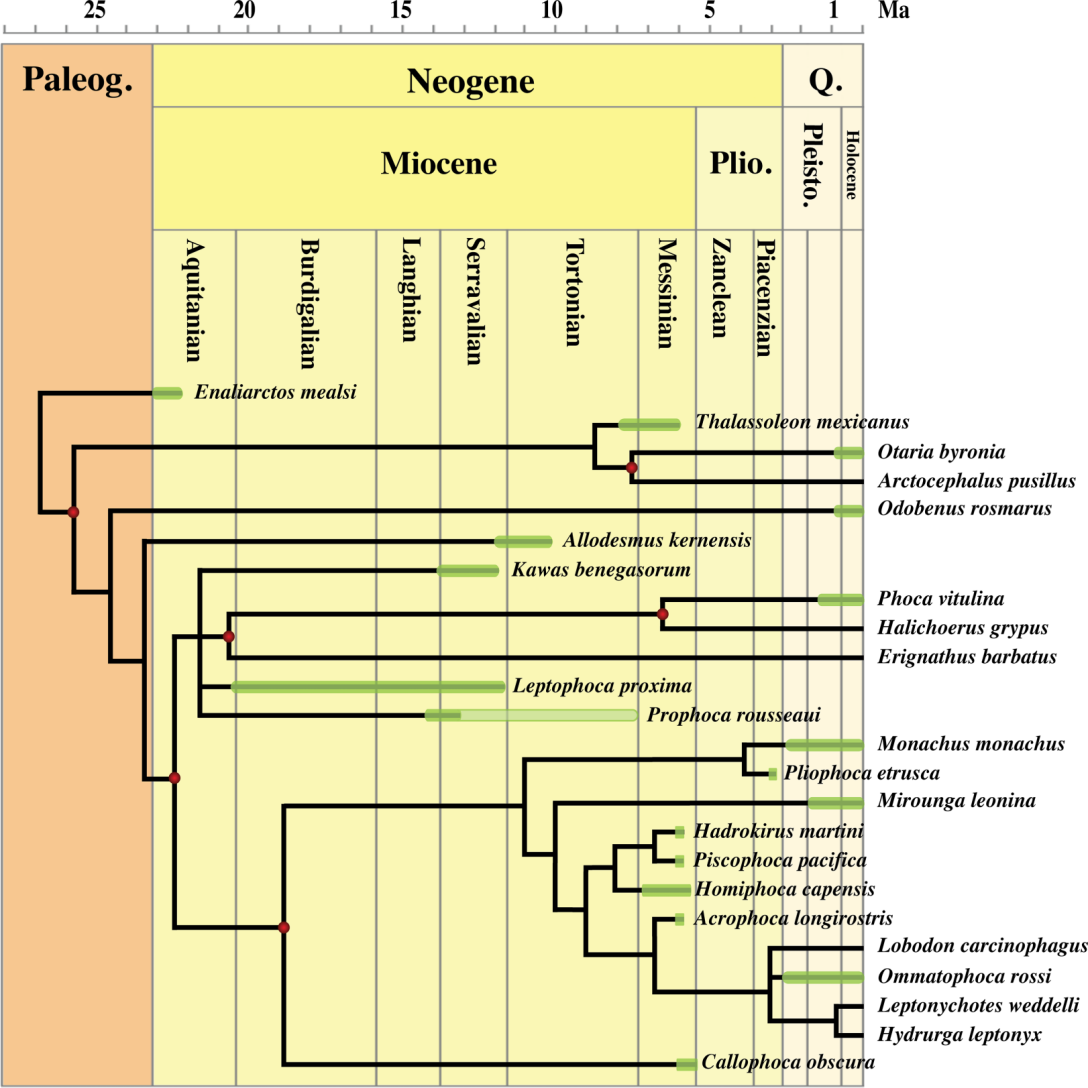
Stratigraphically calibrated strict consensus phylogenetic tree of Phocidae. Phylogenetic analysis performed with PAUP version 4.0b10 for Macintosh (Swofford, 2002). Stratigraphic range data for extinct Monachinae derived from http://www.paleobiodb.org/ and published accounts for each taxon. Stratigraphic range for *Leptophoca proxima* based on biostratigraphic data presented in this study and unpublished stratigraphic data. Stratigraphic range of *Prophoca rousseaui* possibly extendable to 7.2 Ma based on biostratigraphy. Geological time scale based on Cohen et al. (2013). Red dots represent corrected divergence dates at major nodes, published by Higdon et al. (2007). Other nodes are graphical heuristics and do not reflect time-calibrated divergence dates. Abbreviations: Paleog., Paleogene; Q., Quaternary; Pleisto., Pleistocene.

The phylogenetic analysis resulted in twenty-four most parsimonious trees with tree length 188 after 93,764 tried rearrangements. The consistency index is 0.4949, the consistency index excluding the parsimony uninformative character is 0.4898, the retention index is 0.7642, and the rescaled consistency index is 0.3782. The strict consensus tree and the 50% bootstrap tree, and the bootstrap values are presented in Figs. 16 (strict consensus) and 17 (50% bootstrap consensus) and discussed below.

**Figure 17:**
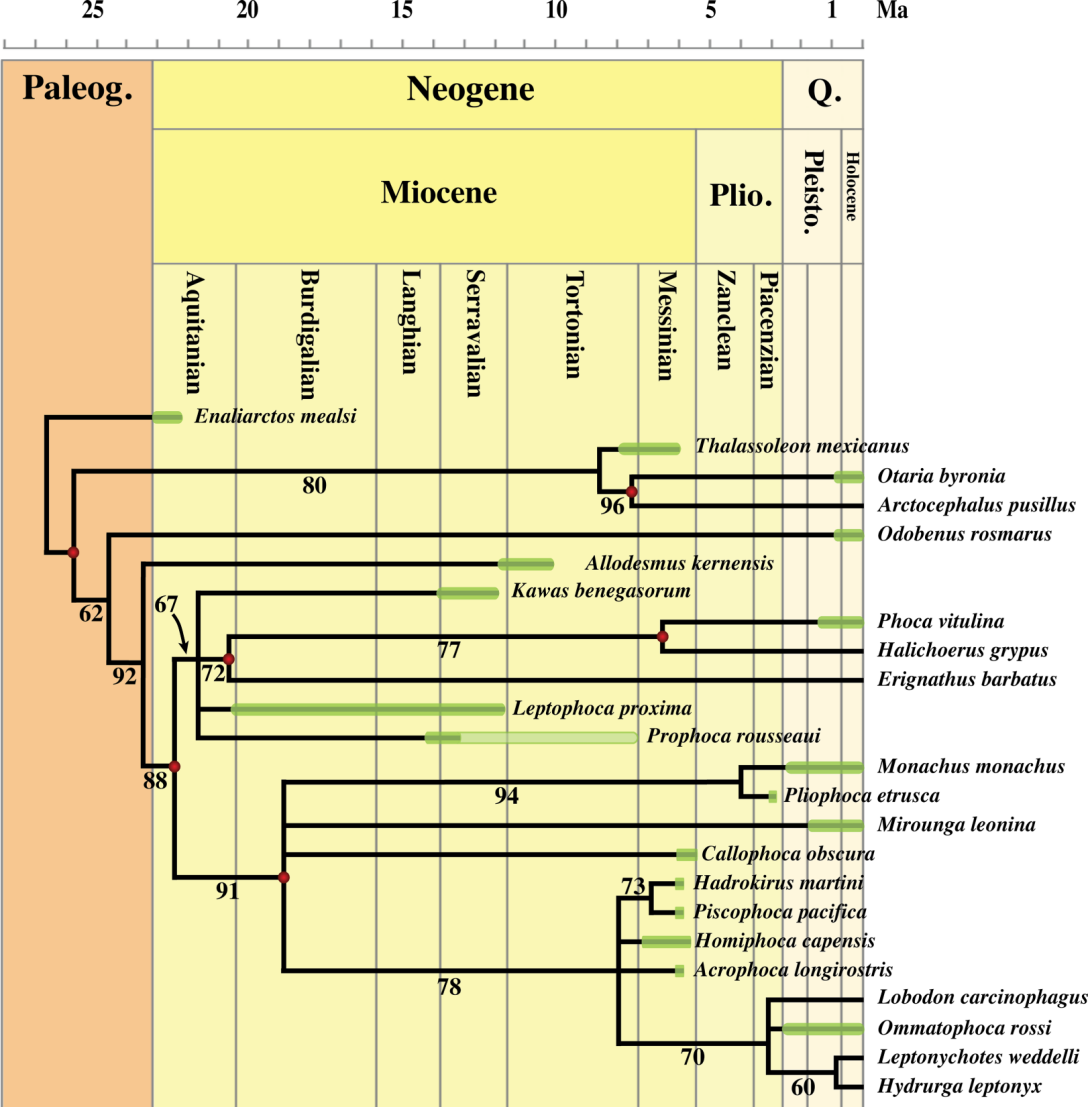
Stratigraphically calibrated bootstrap consensus phylogenetic tree of Phocidae. Phylogenetic analysis performed with PAUP version 4.0b10 for Macintosh (Swofford, 2002). Bootstrap values after 10,000 replicates. Stratigraphic range data for extinct Monachinae derived from http://www.paleobiodb.org/ and published accounts for each taxon. Stratigraphic range for *Leptophoca proxima* based on biostratigraphic data presented in this study and unpublished stratigraphic data. Stratigraphic range of *Prophoca rousseaui* possibly extendable to 7.2 Ma based on biostratigraphy. Geological time scale based on the Cohen et al. (2013). Red dots represent corrected divergence dates at major nodes, published by Higdon et al. (2007). Other nodes are graphical heuristics and do not reflect time-calibrated divergence dates. Abbreviations: Paleog., Paleogene; Q., Quaternary; Pleisto., Pleistocene.

With *Allodesmus kernensis*, *E. mealsi*, *O. rosmarus* and Otariidae as outgroups, Monachinae and Phocinae are both monophyletic and sister groups. The results of the phylogenetic analysis of the Monachinae are presented and discussed by Amson & de Muizon (2014) and will not be re-discussed here. The presented higher-level relationships among pinnipeds are also supported by other morphological phylogenetic studies (e.g., Berta & Wyss, 1994), with Odobenidae being a sister group to the Desmatophocidae + Phocidae and with Desmatophocidae being a sister group to the Phocidae. In the current analysis, *K. benegasorum*, *Leptophoca proxima*, and *Prophoca rousseaui* group together as stem Phocinae, branching off prior to the lineage of *Erignathus*. In the strict consensus tree, *K. benegasorum* and *Prophoca rousseaui* form a polytomy with the *Leptophoca–Erignathus–Halichoerus–Phoca* clade (Fig. 16). In the 50% bootstrap consensus tree, *K. benegasorum, Leptophoca proxima*, and *Prophoca rousseaui* form a polytomy with the *Erignathus–Halichoerus–Phoca* clade (Fig. 17). With only very few characters scored for *K. benegasorum* (21/89; 24%), *Prophoca rousseaui* (16/89; 18%), and *Leptophoca proxima* (22/89; 25%), this polytomy could become better resolved pending the discovery of more complete specimens for each of these three species. However, bootstrap values support an early branching of all three species off the Phocinae lineage.

Phocinae are supported by fifteen synapomorphies, of which seven are unambiguous. The synapomorphies are: premaxilla–maxilla suture lateral to nasal cavity (character 2, state ‘1’ to ‘0’); least interorbital width in anterior portion of interorbital bridge (character 14, state ‘0’ to ‘1,’ unambiguous but also characteristic for Desmatophocidae and *Atopotarus courseni* Downs, 1956); major axes of the glenoid fossae slightly convergent posteriorly (character 16, state ‘0’ to ‘1,’ unambiguous); posterior opening of the carotid canal being not visible in ventral view (character 20, state ‘0’ to ‘1,’ unambiguous); posterior angle of the tympanic withdrawn and losing contact with exoccipital (character 23, state ‘2’ to ‘1’); mastoid being visible in dorsal view (character 26, state ‘0’ to ‘1’); transverse processes of the atlas oblique in lateral view (character 48, state ‘1’ to ‘0’); tubercle and lamina of transverse process of cervical vertebrae 3–6 being clearly isolated from each other (character 49, state ‘0’ to ‘1,’ unambiguous); supraspinous fossa of scapula smaller than infraspinous fossa (character 52, state ‘1’ to ‘0,’ unambiguous); supinator ridge of the humerus being well-developed (character 56, state ‘1’ to ‘0’); deep groove for *extensor digitorum communis* tendon on humerus (character 59, state ‘0’ to ‘1’); palmar process on cuneiform (character 62, state ‘1’ to ‘0’); short metacarpal I (character 74, state ‘1’ to ‘0,’ unambiguous); large manual claws (character 66, state ‘1’ to ‘0,’ unambiguous); and a strongly developed post-tibial fossa (character 81, state ‘0’ to ‘1’).

The *Erignathus–Halichoerus–Phoca* clade differs from the early branching *K. benegasorum*, *Leptophoca proxima*, and *Prophoca rousseaui* by four synapomorphies: deltopectoral crest limited to proximal half of the humerus (character 53, state ‘0’ to ‘1’); a large lesser tubercle on the humerus (character 55, state ‘0’ to ‘1’); a deep groove for *extensor digitorum communis* tendon on radius (character 59, state ‘0’ to ‘1’); and a long and narrow femur (character 79, state ‘1’ to ‘0’). However, implementing future discoveries of more complete specimens of these species will confirm or reject the proposed synapomorphies.

Although the current analysis is the first to determine the phylogenetic position of *Prophoca rousseaui* among other phocids, *Leptophoca proxima* has been treated in a few other published analyses. A first analysis by Koretsky (2001) included both *Leptophoca lenis* and *Prophoca proxima*, and both are scored identical for five out of six characters. Only the shape of the lesser tubercle (Koretsky, 2001; character 44) is scored differently. However, we consider the character states from Koretsky (2001) too subjective in the absence of a clear definition of the states. In the resulting phylogenetic tree, *Leptophoca lenis* branches off before *Prophoca proxima* (Koretsky, 2001; Fig. 51). It should be noted that other results of Koretsky’s (2001) phylogenetic tree strongly depart from morphological, molecular and total evidence trees published by other researchers (e.g., Berta & Wyss, 1994; Bininda-Emonds & Russell, 1996; Higdon et al., 2007; Fulton & Strobeck, 2010; Amson & de Muizon, 2014; Berta et al., 2015).

A more recent phylogenetic analysis focusing on Monachinae in general, and *P. etrusca* specifically, considers *Leptophoca lenis* as a stem monachine (with *Cystophora* and *Erignathus* representing Phocinae) (Berta et al., 2015). However, the authors explicitly state that “our placement of *Leptophoca* as a stem monachine is tentative and will require further study because it differs markedly from previous studies, in which this taxon is consistently allied with phocines (Ray, 1976a; Koretsky, 2001)” (Berta et al., 2015: 20–21). In the latter analysis, the placement of *Leptophoca lenis* among Monachinae has a bootstrap value of 61 (morphological) to 62 (total evidence) (Berta et al., 2015). Comparable to the value of 67 from the current analysis grouping *K. benegasorum*, *Leptophoca proxima*, and *Prophoca rousseaui* among Phocinae (Fig. 17). When excluding *Allodesmus kernensis* from the analysis, bootstrap values for the inclusion of *K. benegasorum*, *Leptophoca proxima*, and *Prophoca rousseaui* increases significantly, to a value of 90. When excluding *Prophoca rousseaui* and *K. benegasorum* from the current analysis, *Leptophoca proxima* is considered an early branching phocine forming a polytomy with *E. barbatus* and the *Halichoerus–Phoca* clade, a relationship strongly supported by a bootstrap value of 92. Similarly, we tested the phylogenetic position of *Prophoca rousseaui*, excluding *Leptophoca proxima* and *K. benegasorum* from the analysis. The very incomplete scoring of *Prophoca rousseaui* resulted in the apparent position of *Prophoca rousseaui* as a stem phocid, which is unlikely, considering the number of osteological features clearly relating the species to crown phocids (this study).

The monachines *A. libyca*, *A. changorum*, and *P. argentinus* have been scored as well, but were omitted from the final analysis (see Supplemental Information 5). In our own preliminary phylogenetic analysis *A. libyca* can be scored for only 2 characters (2%) and its apparent resolution as an otariid does not match the geographic and temporal distribution of extinct and living otariids (see, e.g., Deméré, Berta & Adams, 2003; Churchill, Boessenecker & Clementz, 2014). *P. argentinus* returned as a stem monachine in the strict consensus tree, a result that fits with the early to middle Miocene age assigned to the species (19–14 Ma) (de Muizon & Bond, 1982). However, the taxon was excluded from the final analysis because the 50% bootstrap consensus tree resulted in a large polytomy among Monachinae. *A. changorum* could be scored for 13 characters (14%); its phylogenetic position within Monachinae is poorly resolved and, therefore, it has been excluded from the final phylogenetic analysis pending the examination of additional material. *Monotherium* has not been included in the analysis either: including over five species described on the basis of different non-overlapping parts of the skeleton (compare, Guiscardi, 1871; Van Beneden, 1876, 1877; Ray, 1976b), this genus should be reexamined prior to inclusion in a phylogenetic analysis.

## Discussion

### Functional anatomy related to locomotion in *Leptophoca* and *Prophoca*

Although the exact relationship between *Leptophoca proxima* and *Prophoca rousseaui* remains questionable due to the poorly known second species (see above), both are early-branching phocines. Being dated to the late Langhian to early Serravallian (this work), *Leptophoca proxima* and *Prophoca rousseaui* are also the oldest known phocines (see further below).

The humerus of both *Leptophoca proxima* and *Prophoca rousseaui* retains primitive features at the level of the lesser tubercle and the deltopectoral crest. The lesser tubercle is strongly reduced in both taxa, compared to extant phocids. A similar state can be observed in the outgroups, the extant otariids and *Odobenus*, and terrestrial carnivorans, the extinct lobodontins *A. longirostris* and *P. pacifica*, and many other fossil phocids from the Paratethys (Koretsky, 2001) and the southern margin of the North Sea Basin (e.g., Koretsky, Peters & Rahmat, 2015). Consequently, the presence of only a small lesser tubercle is probably ancestral to all phocids and an increase of size may have evolved independently in both subfamilies. de Muizon (1981) explains the locomotion implications of a reduced lesser tubercle elaborately, but it primarily indicates a more frequent and powerful use of the forelimb for propulsion when swimming, compared to the very limited use of the forelimbs for propulsion in extant phocids.

Considering the deltopectoral crest of extant phocines and extant monachines, its shape generally strongly differs between both subfamilies (e.g., King, 1966; Hendey & Repenning, 1972; de Muizon, 1981). In extant phocines, the deltopectoral crest is limited to the proximal half of the humerus, abruptly terminating distally. In lateral view, it appears roughly rectangular in outline. The proximal deltoid surface is smooth and clearly outlined. In extant monachines, the deltopectoral crest is not limited to the proximal half of the humerus (except *L. weddellii* and *L. carcinophaga*); it forms a prominent anterior projection proximally, gradually tapering toward the diaphysis distally. Also, the proximal deltoid surface is not always clearly distinguishable. Extinct phocid taxa show a variety of deltopectoral crest shapes, intermediate between extant phocines and monachines. The humeri of the early phocines *Leptophoca proxima* and *Prophoca rousseaui* appear intermediate between the humerus of otariids and *Odobenus* and the humerus of extant monachines and phocines (Fig. 3). The deltopectoral crest is not limited to the proximal half of the humerus (shared with Monachinae and Otariidae + *Odobenus*, character 53), but tends to terminate distally abruptly (shared with Phocinae, character 54). However, the ‘abrupt’ distal termination of the deltopectoral crest in *Leptophoca proxima* and *Prophoca rousseaui* is not as abrupt as it is in extant phocines, but rather expresses an intermediate condition. Furthermore, the anterior projection of the deltopectoral crest is not as pronounced as in extant phocines, attaining a condition similar to otariids and *Odobenus*. Hence, it can be proposed that *Leptophoca proxima* and *Prophoca rousseaui* used their fore flippers more actively for aquatic and terrestrial locomotion than extant Phocidae.

Another character (59) shared by *Leptophoca proxima* and *Prophoca rousseaui* is the strong development of a groove for the *m. extensor digitorum communis* tendon on the lateral surface of the radius. The same character state has been observed in the otariids and extinct lobodontins *A. longirostris* and *P. pacifica*. For the latter two species, de Muizon (1981) argues that a deep groove for the tendon of *m. extensor digitorum communis* points toward enhanced flexion of the manus. This condition may indicate an increased use of the fore flipper during aquatic locomotion (braking, propulsion, and steering) or terrestrial locomotion.

Slight eversion and the poor development of a gluteal fossa on the lateral surface of the ilium are other characters shared by early branching phocids (see, de Muizon, 1981 for *A. longirostris* and *P. pacifica*). Moreover, the relatively long ilium of *Prophoca rousseaui* can also be considered a basal character (Berta & Ray, 1990). In early pinnipedimorphs (outgroup *E. mealsi*) and other pinnipeds (outgroups Otariidae and *Odobenus*), the ilium is not or very slightly everted and the gluteal fossa on the lateral surface of the ilium is not developed. Similarly, the ilium is much longer in these taxa than it is in extant phocids. Thus, eversion and shortening of the ilium are apomorphies shared by all extant and extinct Phocidae. Yet, the condition is more pronounced in Phocinae than in Monachinae. Although the innominates of extinct Phocidae such as *A. longirostris*, *Leptophoca proxima*, *P. pacifica*, and *Prophoca rousseaui* are clearly phocid, they can be considered intermediate between more crownward Phocidae and Otariidae + Odobenidae. The development of a deep gluteal fossa on the ilium is a trait acquired relatively recently by Phocinae: many extinct phocines such as *Leptophoca proxima* (Koretsky, 2001; for *Leptophoca lenis*), *Phoca vitulinoides*, and *Prophoca rousseaui* (Leonard Dewaele, 2016, personal observation) have no—or only a weakly developed—gluteal fossa. In extant phocines, the early branching *E. barbatus* has only a weakly developed gluteal fossa. A weakly developed gluteal fossa does not necessarily imply weakly developed gluteus muscles (de Muizon, 1981). In general, published functional morphological and myological studies of phocid hindlimbs are rare, and because it is beyond the scope of the present study, we do not attempt to identify any locomotive implications of the morphology of the innominates in *Leptophoca proxima* and *Prophoca rousseaui* in the current study.

### Paleobiogeography of middle Miocene monachines and phocines

#### Middle Miocene taxa and their provenance (including Monachinae)

Historically, both the oldest unequivocal monachine and the oldest known Miocene phocine are known from the middle Miocene (Langhian) Calvert Formation of Maryland and Virginia (True, 1906; Ray, 1976a, 1976b; Deméré, Berta & Adams, 2003). Presumably older specimens and species have been described (Koretsky & Sanders, 2002; Diedrich, 2011), but their age assignments are contested further in the current study. *Leptophoca proxima* (formerly as *Prophoca proxima* and *Leptophoca lenis*) has commonly been regarded as the oldest known record for the family Phocidae (e.g., Deméré, Berta & Adams, 2003; Koretsky, Ray & Peters, 2012). The holotype of *Leptophoca lenis* (USNM 5359) had been retraced to “zone 10” of the Plum Point Marl Member of the Calvert Formation, which had been dated to the earliest Langhian (True, 1906; de Verteuil & Norris, 1996). Koretsky (2001) reconsidered the origin of USNM 5359 between Chesapeake Beach (zone 5) and Plum Point (zone 10) in the Calvert Formation, adjusting the oldest known occurrence of *Leptophoca* to the middle Burdigalian (approximately 18 Ma). Other specimens of *Leptophoca proxima* from the West Atlantic, presented by Koretsky (2001) can all be retraced to an age corresponding to the Langhian and Serravallian of the Plum Point Member of the Calvert Formation and the Serravalian and early Tortonian Choptank Formation. However, as stated above, the assignment of other isolated specimens to *Leptophoca proxima* should be considered with care. The Belgian lectotype of *Leptophoca proxima* (IRSNB 1146-M279) has a minimum middle Langhian–early Serravallian age (14.8–13.2 Ma) according to dinoflagellate cyst biostratigraphy. Considering *Leptophoca amphiatlantica* as *Leptophoca* cf. *Leptophoca proxima*, the occurrence of *Leptophoca proxima* in the East Atlantic can be dated to the early late Burdigalian–early Langhian Dutch Breda Formation (16.4–15.8 Ma).

The enigmatic monachine *Monotherium wymani* (Leidy, 1854) is known from only few isolated bones and for a long time its stratigraphic position and age remained doubtful. Ray (1976b) re-investigated this subject and identified the uppermost zones of the Calvert Formation at Richmond, Virginia, as the origin of the *Monotherium wymani* material. These zones have been dated to the late Langhian–early Serravallian (Kidwell et al., 2015).

Ray (1976a) also identified one specimen from the Calvert Formation as belonging to *Prophoca rousseaui*, but he did not describe or illustrate it. We could not retrace it at the USNM, nor find any data on a more precise age designation. Hence, we will not consider it in the discussion. Belgian material of *Prophoca rousseaui* had been (re)assigned to the late Burdigalian–Langhian Antwerpen Sands Member of the Berchem Formation (Deméré, Berta & Adams, 2003). Two Belgian specimens of *Prophoca rousseaui* have an age between 14.2 and 13.2 Ma (late Langhian–early Serravallian) and 14.2–7.2 Ma (late Langhian–Tortonian/Messinian boundary). The isolated innominate IRSNB M2234 from the base of the Antwerpen Sands Member of the Berchem Formation at the Posthofbrug, rich in vertebrate remains (Louwye et al., 2010), is here assigned to *Prophoca rousseaui*. Louwye et al. (2010) dated the base of the Antwerpen Sands Member of the Berchem Formation at the Posthofbrug to the latest Burdigalian.

The current body of evidence indeed indicates that *Leptophoca proxima* is the oldest true seal currently known, but we have to admit that the currently limited fossil record of *Monotherium wymani* and *Prophoca rousseaui* may in fact not give a good representation of their stratigraphical distribution. With *Leptophoca proxima* and *Prophoca rousseaui* forming a distinctive group of early branching phocines, exactly distinguishing the related ages of each taxon is of subordinate importance.

Other geologically old, i.e., Burdigalian, Langhian and/or Serravalian, Phocidae are *A. libyca*, *Miophoca vetusta* Zapfe, 1937, *Monotherium gaudini*, and other Phocidae from Libya and the Paratethys (Koretsky, 2001; Deméré, Berta & Adams, 2003; Koretsky, Ray & Peters, 2012; Koretsky & Domning, 2014; Berta et al., 2015) and *K. benegasorum*, and *P. argentinus* from Argentina (Ameghino, 1893; de Muizon & Bond, 1982; Cozzuol, 2001). *A. libyca* may presumably replace *Monotherium wymani* as the oldest known monachine seal. Recovered from the Garat Jahanam Member of the Marada Formation in Lybia, the holotype and only specimen, a partial mandible, may be dated between 19 and 14 Ma (Koretsky & Domning, 2014). *Monotherium gaudini* is a monachine from Italy (Guiscardi, 1871). Deméré, Berta & Adams (2003) degrade *Monotherium gaudini* to *Monotherium* sp. indet. from the Bismantova Formation. However, there is no conclusion for the age of this formation. Odin et al. (1997) dated the Bismantova Formation to the Burdigalian–early Langhian, which had been adopted by Berta et al. (2015), while Deméré, Berta & Adams (2003) consider the late Langhian–Serravalian-early Tortonian age presented by, e.g., Pini (1999) and Carena et al. (2000).

Deméré, Berta & Adams (2003) regard *Pristiphoca vetusa* (sic) (Thenius, 1950) as an early Sarmatian (Serravalian) monachine: the oldestz record of a monachine in the Paratethys. However, it should be pointed out that the species under consideration is in fact a junior synonym to *M. vetusta*, an enigmatic Badenian (middle Miocene) phocid from the Vienna Basin (Zapfe, 1937), and considered to be a “cystophorine” by Koretsky (2001). Piller, Harzhauser & Mandic (2007) considered the Badenian to be Langhian–early Serravallian in age. Nonetheless, *M. vetusta* can be considered a *nomen dubium*, or as Grigorescu (1976: 416) states it: “*M. vetusta* (…) is known only by a fragment of the lower jaw, that can’t represent a satisfactory basis for detailed discussions.” However, a detailed revision of *M. vetusta* and its age is beyond the scope of this study.

*Properiptychus argentinus* is an early monachine from the Paraná Formation of Argentina. de Muizon & Bond (1982) assigned a Friasian age to the species, which is a South American Land Mammal Age roughly spanning from 16.3 to 15.5 Ma, (late Burdigalian to the early Langhian; Flynn & Swisher, 1995). However, more recent research assigns a late Miocene age to the Paraná Formation on the basis of bivalve biostratigraphy (Pérez, Genta Iturrería & Griffin, 2010).

According to Koretsky (2001), *Praepusa* spp., *C. maeotica*, *S. sintsovi* and *M. pontica* from the Central and Eastern Paratethys are all known from Sarmatian (Langhian and Serravallian) deposits. More recent stratigraphic research by Piller, Harzhauser & Mandic (2007) supported the ages Koretsky (2001) assigned to the Sarmatian stages.

#### Remarks on allegedly Eocene and Oligocene Phocidae

The early evolution of Phocidae is poorly known and the two oldest records are a matter of debate. The presumably oldest described phocid is the species *Praephoca bendullensis* Diedrich, 2011 proposed to date from the Eocene of northern Germany (Diedrich, 2011). The type—and single—specimen (an isolated femur) had allegedly been found in the early Lutetian (early middle Eocene; 49–45 Ma) Fürstenau Formation. This geologic age predates the formerly generally accepted earliest pinnipedimorph records by at least 15–20 Ma and the author even suggests a late Paleocene/early Eocene age for the specimen, based on its reworked nature. The oldest known pinnipedimorphs are *Enaliarctos* Mitchell & Tedford, 1973 (late Oligocene Yaquina Formation of Oregon, USA; Berta, 1991), *Potamotherium* Geoffroy, 1833 (late Oligocene lake sediments of Rheinland-Pfalz, Germany; Mörs & von Koenigswald, 2000; phylogenetic affinities with pinnipeds still debated), or *Puijila* Rybczynski, Dawson & Tedford, 2009 (early Miocene Haughton Formation of Nunavut, Canada; Rybczynski, Dawson & Tedford, 2009). However, *P. bendullensis* has been proposed to represent a phocid, thus, antedating the oldest unambiguously known Phocidae by approximately 30 Ma (see below) and creating multiple Eocene and Oligocene ghost lineages for all pinnipedimorphs. It also antedates published molecular divergence dates for Arctoidea Flower, 1869 (e.g., Higdon et al., 2007 as well as the oldest arctoid fossils, e.g., *Parictis* Scott, 1893 from the late Eocene/early Oligocene of North America (e.g., Hunt, 1998).

Diedrich (2012) formally described the Fürstenau Formation in another paper, published almost simultaneously to the description of *P. bendullensis.* Currently only two partial sections of the Fürstenau Formation have been described, both in Germany, based on the correlation of a fossiliferous gravel bed (Diedrich, 2012): the lower part of the section at Dalum, and the upper part at Osteroden. The middle Eocene age proposed for the Fürstenau Formation is entirely based on the relative dating of the fossil content (terrestrial mammal teeth and shark teeth) of only one fossiliferous layer. This layer is a condensed basal gravel and the published fossil content of this fossiliferous layer may not be a good representative for the entire fossil content—and geologic age—of this layer. Indeed, the fossil content is strongly biased to sharks (approximately 95% of the material), with illustrated specimens showing signs of reworking and abrasion. A more thorough biostratigraphic investigation may potentially reveal a time averaged nature of the fossil content of this layer, possibly containing a mix of fossils from different Paleogene levels. The partial femur attributed to *P. bendullensis* had been collected during the 1980s, separately from the study presented by Diedrich (2011, 2012). Furthermore, it is strongly abraded and was most likely reworked. Its stratigraphic origin in the Fürstenau Formation is thus at best questionable, considering the worldwide lack of any other Eocene pinnipedimorph records despite extensive sampling of other marine mammal fossils from Eocene marine deposits around the world, i.e., Indo-Pakistan, North Africa, southwestern USA, and New Zealand (e.g., Thewissen, Williams & Hussain, 2001; Peters et al., 2009; Peredo & Uhen, 2016). No details are provided about the overlying Neogene layers in the Dalum section (Diedrich, 2012), and the potential origin of the specimen in another, younger gravel was not discussed by the author. Unlike Koretsky & Domning (2014: 227) who stated that “the only known specimen, the proximal part of the femur, is so damaged that it is inadequate for its identification as a pinniped,” we support the identification of this specimen as representing a phocid by, considering the anteroposterior flattening of the diaphysis, the absence of a fovea capitis and the absence of a lesser trochanter (see Diedrich, 2011). Based on the small size of the specimen, it may tentatively be related to the Miocene phocines *Batavipusa neerlandica* or *Phoca vitulinoides*, known from the Netherlands and Belgium (Koretsky & Peters, 2008; Van Beneden, 1871, 1876, 1877), but the poor state of preservation of the specimen does not permit a detailed description and comparison; the definition of a separate taxon seems thus hazardous. Therefore, we consider *Praepusa bendullensis* a *nomen dubium*.

Koretsky & Sanders (2002) described two indeterminate phocid femora from the upper Oligocene Ashley and Chandler Bridge formations of South Carolina, USA. Although the oldest formally described Phocidae from the east coast of North America date from the late early to early middle Miocene, it has been assumed that the Phocidae originated during the latest Oligocene or the earliest Miocene, based on molecular evidence (Higdon et al., 2007). Such evidence renders it possible that the specimens presented by Koretsky & Sanders (2002) are indeed from the Chandler Bridge Formation, but not from the Ashley Formation, based on age definitions of both formations (Weems et al., 2012). However, at the localities where these pinniped remains were found, just south to southwest of Summerville, SC, USA, the Ashley and Chandler Bridge formations only outcrop in ditches and scarps, and are covered by the “Ashley Phosphate Beds,” the Pleistocene Wando Formation and Penholoway Formation (Weems, Lewis & Lemon, 2014). Hence, in the absence of a firmly established stratigraphic framework for the specimens, it is most likely that these specimens, which have been found in ditches, have been washed to lower levels in the ditch from the overlying Ashley Phosphate Beds or Wando Formation and that the specimens are found ex situ. The Ashley Phosphate Beds are usually identified as the basal phosphatic layer of the Wando Formation and are rich in reworked Miocene and Pliocene fossils (Monsch, Fierstine & Weems, 2005). In the absence of a more precise geographic and stratigraphic position for both specimens, their Oligocene provenance should probably only be considered as a hypothesis to be confirmed. Keeping the fragmentary nature of the specimens in mind, a shallow trochanteric fossa has also been observed in the late Neogene phocine *Phocanella* from Belgium and the east coast of North America (Koretsky & Ray, 2008).

#### Early phocid paleobiogeographic evolution

While early-branching pinnipedimorphs, early Odobenidae, early Otariidae, and Desmatophocidae diversified in the North Pacific Ocean during the early and middle Miocene (see, e.g., Deméré, Berta & Adams, 2003; Boessenecker & Churchill, 2015), specimens of *Monotherium wymani* and *Leptophoca proxima* from the east coast of North America represent the earliest unambiguous records of Phocidae. Most scientists agree on the migration of a phocid ancestor from the northeastern Pacific to the northwestern Atlantic (see Deméré, Berta & Adams, 2003). Of the two possible pathways, through the Central American Seaway or through the Bering Strait and Arctic Ocean, the first is the prevailing option (Costa, 1993; Bininda-Emonds & Russell, 1996; Deméré, Berta & Adams, 2003). During the early and middle Miocene, the Beringian land bridge prevented the marine connection of the North Pacific Ocean to the Arctic Ocean (Costa, 1993; Bininda-Emonds & Russell, 1996; Marincovich, 2000; Deméré, Berta & Adams, 2003). However, as Deméré, Berta & Adams (2003: 63) pointed out, “the absence of basal phocid fossils in the North Pacific and Caribbean is a weakness of this hypothesis.” Other possible regions of origin for Phocidae that have been considered include the Paratethys (Orlov, 1933; McLaren, 1960; Koretsky & Barnes, 2006) and the Arctic (Rybczynski, Dawson & Tedford, 2009). Those are difficult to unite with the paleobiogeography and chronology of early and middle Miocene diversification of other pinnipedimorphs (see Deméré, Berta & Adams, 2003), given the generally accepted monophyly of pinnipeds and pinnipedimorphs both on morphological and molecular evidence (e.g., Berta & Wyss, 1994; Bininda-Emonds & Russell, 1996; Higdon et al., 2007; Fulton & Strobeck, 2010; Árnason et al., 2006). However, a number of geologically old Phocidae have been found outside the East coast of North America: *A. libyca*, *P. argentinus*, several phocids from the Paratethys, and *Leptophoca proxima* specimens from the Netherlands may rival *Leptophoca proxima* and *Monotherium wymani* as the oldest known phocids.

Although these findings put the hypothesis of the East coast of North America as the area of origin of Phocidae to the test, the widely recognized monophyletic origin of pinnipeds renders it difficult to consider the Paratethys as the origin of Phocidae in relation to its Pacific ancestors and relatives. However, early diversification of crown Phocidae presumably occurred during the late Burdigalian or early Langhian in the North Atlantic. This is supported by (1) the co-occurrence of both a monachine (*Monotherium wymani*) and a phocine (*Leptophoca proxima*) in the Langhian–lower Serravallian Calvert Formation, as well as (2) the presence of the phocines *Leptophoca proxima* and *Prophoca rousseaui* and the monachine *A. libyca* in the North Sea and in Lybia, respectively, during the same time interval. *P. argentinus* from Argentina may be a stem-monachine that ventured into the South Atlantic. Pending the discovery of other, more complete, fossil monachines from the Southern Hemisphere, *P. argentinus* and other may represent a basal monachine branching off the lineage that would ultimately diversify in the Southern Hemisphere, leading to later fossil monachines and modern members of the subfamily.

Specimens of *Leptophoca* found in Belgium and the Netherlands (this work; Koretsky, Ray & Peters, 2012), on the southern part of the North Sea Basin indicate that shortly after their population of the east coast of North America, *Leptophoca* must have migrated across the North Atlantic Ocean. Given the poor fossil record of *Prophoca rousseaui*, it is unknown as to whether *Prophoca* originated along the North American east coast and migrated to the North Sea Basin, just as *Leptophoca proxima*, or that *Prophoca* diverged from *Leptophoca* in the North Sea and migrated back across the Atlantic to North America. Based on the diversity of middle and late Miocene phocids, the North Sea Basin and the Paratethys must have been the regions where phocids diversified in the Northern Hemisphere during the middle and late Miocene (see, Koretsky, 2001; Deméré, Berta & Adams, 2003).

It should be noted that fossil marine mammal finds (including cetaceans) from deposits underlying the Berchem Formation in the Antwerp area are generally very rare. Hence, the absence of phocid specimens antedating these layers may be related to a preservational bias and not necessarily to the absence of Phocidae in the North Sea at that time. Published data on the relative abundance of pinnipeds in other fossil-rich marine strata, such as the Purisima Formation (Boessenecker, Perry & Schmitt, 2014) and the Sharktooth Hill bonebed (Pyenson et al., 2009) in California show relatively low abundances for pinnipeds in comparison to other vertebrate taxa. For the Neogene strata of Belgium, no detailed analysis has been undertaken about the relative abundances of fossil marine mammal taxa. However, a concise qualitative analysis by Louwye et al. (2010) from one horizon of the Berchem Formation at Posthofbrug also indicates that odontocete remains are much more common and diverse than phocid remains.

During the late middle Miocene, following the initial diversification in the North Atlantic and the Paratethys Phocinae ventured into the South Atlantic Ocean; roughly 5 million years younger than the monachine *P. argentinus*, *K. benegasorum* is currently the oldest and only known phocine seal from the Southern Hemisphere.

## Conclusion

The original understanding of *Prophoca proxima* (Van Beneden, 1877) was contested within a few decades after being named (see True, 1906). True (1906) noted the marked similarities between *Prophoca proxima* and *Leptophoca lenis*. However, he found the differences outweighed the similarities and left *Prophoca proxima* in limbo. Ray (1976a) renamed *Prophoca proxima* to *Leptophoca proxima*, but kept *Leptophoca lenis* as a separate species. Here, we redescribe both *Prophoca rousseaui* and *Prophoca proxima*, considering *Prophoca proxima* and *Leptophoca lenis* to represent the same species: *Leptophoca proxima* n. comb. Moreover, we tend to consider *Leptophoca amphiatlantica* (Koretsky, Ray & Peters, 2012) Phocidae aff. *Leptophoca*, neither strictly rejecting the possibility of a separate species *Leptophoca amphiatlantica* nor degrading it to a junior synonym to *Leptophoca proxima.*

Based on the phylogenetic analysis presented in this study (see earlier), *Leptophoca proxima* represents an early-branching phocine seal. Given the scarcity of the fossil record of *Prophoca rousseaui*, the species’ phylogenetic position is less unequivocal as *Leptophoca proxima*’s. However, it can be argued that *Prophoca rousseaui* also represents an early-branching phocine as well. Both species bear derived phocine characters as well as plesiomorphic pinniped and monachine characters, suggesting for this character an intermediate position between a hypothetical ancestral phocid and younger extinct and extant phocines.

Although the current fossil record supports the assumption of *Leptophoca proxima* as the oldest known phocine seal, the scarcity of the fossil record of *Prophoca rousseaui* renders it unclear as to whether *Leptophoca proxima* is really the oldest known true seal. Notwithstanding, the current fossil record suggests that Phocidae may have originated along the North American east coast and rapidly diverged as well as rapidly migrated across the Atlantic, entering the North Sea Basin and the Paratethys, where they further diverged.

## Supplemental Information

10.7717/peerj.3024/supp-1 Supplemental Information 1 Measurements.

10.7717/peerj.3024/supp-2 Supplemental Information 2 Dinoflagellate cyst biostratigraphy.

10.7717/peerj.3024/supp-3 Supplemental Information 3 Measurements sacrum.

10.7717/peerj.3024/supp-4 Supplemental Information 4 Phylogenetic characters.

10.7717/peerj.3024/supp-5 Supplemental Information 5 Phylogenetic matrix.

## Institutional Abbreviations

CMM: Calvert Marine Museum, Solomons, Maryland, USA
IRSNB: Institut Royal des Sciences Naturelles de Belgique (‘M’ representing type and figured specimens from the fossil mammal collection), Brussels, Belgium
MAB: Oertijdmuseum de Groene Poort, Boxtel, the Netherlands
MNHN: Muséum national d’Histoire naturelle, Paris, France
USNM: Department of Paleobiology, National Museum of Natural History, Smithsonian Institution, Washington, D.C., USA.

## Additional Information and Declarations

### Data Deposition

The following information was supplied regarding data availability: The raw data has been supplied as Supplementary Dataset Files.

### New Species Registration

The following information was supplied regarding the registration of a newly described species: Leptophoca proxima n. comb. (Van Beneden, 1876) urn:lsid:zoobank.org:pub:23248E40-CFED-4E41-912B-2702480A67B6.
