# Supplementary material for: On Prophoca and Leptophoca (Pinnipedia, Phocidae) from the Miocene of the North Atlantic realm: redescription, phylogenetic affinities and paleobiogeographic implications
Source: PeerJ. 2017 Feb 21;5:e3024. doi: 10.7717/peerj.3024 (PMC5322758; doi:10.7717/peerj.3024)
Supplement: Supplemental Information 1 [file peerj-05-3024-s001.docx]

**Supplemental Information 1: Measurements**

This file includes two tables, showing selected length measurements of *Leptophoca proxima* and *Prophoca rousseaui*.

All measurements were obtained with a mechanical caliper to 0.1 mm.

For reasons of consistency, measurements follow the scheme employed by Koretsky (2001) and Koretsky, Ray and Peters (2012).

Table 1: Measurements of the humerus of one specimen of *Prophoca rousseaui* and four specimens of *Leptophoca proxima*, with measurements of the Belgian lectotype of *Leptophoca proxima* in bold. Measurements of other specimens of *Leptophoca proxima* are in bold if they are larger than for the lectotype. Measurements presented in mm. ‘+’ indicating that depicted measurement is minimum length.

|  | ***Prophoca rousseaui*** | ***Leptophoca proxima*** | | | | |
| --- | --- | --- | --- | --- | --- | --- |
|  | **Belgium** | **Belgium** | **United States** | | | |
|  | IRSNB 1149-M274 | **IRSNB 1146-M279** | USNM 412115 | USNM 23450 | USNM 5359 | USNM 284721 |
| Total length |  |  | 131.7 | 113.4 | 124.5 | 126.2 |
| Length deltopectoral crest |  | **+ 86,7** | 82.8 | c. 67.2 | 76.4 | 72.6 |
| Height capitulum |  | **28.3** | c. 25.5 | c. 21.9 | c. 27.8 | c. 24.2 |
| Height trochlea |  |  | 16.1 |  | 17.0 | 16.4 |
| Width capitulum |  | **32.1** | c. 25.1 | 26.9 |  |  |
| Width deltopectoral crest |  | c. 25.2 | c. 19.7 |  | 23.3 | **25.5** |
| Width distal epiphysis |  |  | 38.0 | c. 34.6 | 42.3 | 37.5 |
| Width proximal epiphysis |  | **46,3** | 38.0 | 37.7 |  |  |
| Distal width trochlea |  |  | 24.2 |  | 29.8 | 30.2 |
| Width trochlea in anterior view |  |  | 18.3 |  | 19.8 | 18.3 |
| Transverse width diaphysis | c. 30.2 | **16.5** | 14.4 | 13.5 | 14.9 | 15.0 |
| Thickness proximal epiphysis |  |  | 58.7 |  | c. 57.0 | 54.8 |
| Thickness medial condyle |  |  | 27.0 |  | 28.1 | 25.5 |
| Thickness lateral condyle |  |  | 22.7 | c. 29.4 | 23.5 | 22.0 |
| Diameter diaphysis at deltopectoral crest | 48.8 | 36.4 | **37.3** |  | 32.1 | 30.5 |

Table 2: Measurements of the only known femur of *Prophoca rousseaui*. Measurements in mm. ‘+’ indicating that depicted measurement is minimum length.

|  | IRSNB 1150 Ct.M. 277a |
| --- | --- |
| Lateral length, parallel to long axis | 124.7 |
| Length of lateral condyle | 34.0 |
| Width distal epiphysis, perpendicular to long axis | c. 79.3 |
| Width of condyles | 70.9 |
| Height of patellar surface | 32.5 |
| Width diaphysis | 39.7 |
| Thickness diaphysis | 25.3 |
| Length greater trochanter | + 40.1 |
| Thickness greater trochanter | + 30.5 |
| Thickness lateral epiphysis | 45.3 |
| Intercondylar width | 18.3 |

Table 3: Measurements of the femora of *Leptophoca proxima* and *Leptophoca amphiatlantica* adopted from Koretsky (2001) and Koretsky, Ray & Peters (2012). Femora of *L. proxima* represent isolated specimens that have been attributed to the species only tentatively. Femora of *L. amphiatlantica* include the holotype, the paratype and other isolated and fragmentary specimens. Measurement ranges vary for both species and for different characters, depending on the possibility to measure on incomplete specimens. Measurements ranges for *L. proxima* based on two to eight specimens and for *L. amphiatlantica* based on one to four specimens. Visual comparison between measurement ranges of both species is graphically represented in Figure 15.

|  | ***Leptophoca proxima*** | ***Leptophoca amphiatlantica*** |
| --- | --- | --- |
| Absolute length | 119.0-120.0 | 96.0-118.3 |
| Medial length | 109.0-112.0 | 109.5 |
| Lateral length | 101.5-112.0 | 94.5-109.4 |
| Length of medial condyle | 21.5-22.0 | 19.7 |
| Length of lateral condyle | 21.0-24.5 | 19.4-19.8 |
| Length of greater trochanter | 28.5-37.0 | 27.1-30.5 |
| Intertrochanteric length | 42.0-48.0 | 38.0-48.8 |
| Height of caput | 23.5-25.5 | 18.3-19.0 |
| Height of articular area of patellar surface | 23.5-24.0 | 20.0-23.0 |
| Width of proximal epiphysis | 51.0-59.0 | 47.1-60.9 |
| Width of distal epiphysis | 54.0-62.0 | 46.0-58.8 |
| Width of condyles | 45.0-54.5 | 43.0-51.0 |
| Width of greater trochanter | 16.5-22.5 | 16.3-20.5 |
| Width of caput | 18.0-23.5 | 28.0-23.3 |
| Width of diaphysis | 26.0-35.0 | 23.7-30.9 |
| Thickness of diaphysis | 14.0-17.0 | 15.3-16.5 |
| Thickness of medial condyle | 27.0-27.5 | 30 |
| Thickness of lateral condyle | 27.0-31.5 | 26.4-31.0 |
| Distance between condyles | 13.0-17.5 | 14.3-15.8 |
| Diameter of neck | 14.5-18.0 | 16.3-18.9 |
