# Supplementary material for: On Prophoca and Leptophoca (Pinnipedia, Phocidae) from the Miocene of the North Atlantic realm: redescription, phylogenetic affinities and paleobiogeographic implications
Source: PeerJ. 2017 Feb 21;5:e3024. doi: 10.7717/peerj.3024 (PMC5322758; doi:10.7717/peerj.3024)
Supplement: Supplemental Information 2 [file peerj-05-3024-s002.docx]

**Supplemental Information 2: Dinoflagellate cyst biostratigraphy**

**Methods**

Three sediment samples recovered from bone cavities of specimens IRSNB 1150-M277b (*Prophoca rousseaui*, tibia), IRSNB 1146-M279 (*Leptophoca proxima*, humerus), and IRSNB 1192-M276 (*Prophoca rousseaui*, lumbar vertebrae) were palynologically analysed for organic-walled dinoflagellate cysts (dinocysts), acritarchs and fungal remains (see table 1).

The maceration of the sediments followed standard techniques (Louwye et al., 2004) involving treatments with HCl and HF for the removal of carbonates and silicates, respectively. The organic residue was sieved on a nylon screen with a mesh size of 16 μm and afterwards mounted on a glass slide with glycerol gelatine jelly. The microscopic analysis was carried out with a transmitted light microscope Zeiss AxioImager A1 with a magnification of 400x. The entire slide was scanned in non-overlapping traverses and the occurrence palynomorphs was noted. The taxonomy of the dinocysts and acritarchs follows Fensome, McRae & Williams (2008).

**Sample from *Prophoca rousseaui* IRSNB 1150-M277b**

The preservation of the marine palynomorphs is moderate to good. A total of 22 dinocysts, and three acritarch species were recorded.

The presence of *Habibacysta tectata* provides a maximum age for the sample. The lowest occurrence of *Habibacysta tectata* in the high latitudes of the Iceland Sea is dated at 14.2 Ma by Schreck et al. (2012) in a magnetostratigraphic calibrated study of dinocyst bio-events. This date was later corroborated by Quaijtaal et al. (2014) in a high-resolution study of the Miocene in lower latitudes (Porcupine Basin, off southwest Ireland). The minimum age of the sample is determined by the presence of *Hystrichosphaeropsis obscura*, a species with a highest occurrence at the upper boundary of the eponymous biozone in Denmark (Dybkjaer & Piasecki, 2010). The age of the *Hystrichosphaeropsis obscura* Zone is 8.8 Ma to 7.6 Ma. *Labyrinthodinium truncatum* has a highest occurrence near the upper bundary in the *Hystrichosphaeropsis obscura* Zone in Denmark (Dybkjaer & Piasecki, 2010), and confirms the minimum age of the sample. An age between 14.2 Ma and 7.6 Ma (late Langhian – late Tortonian) can be proposed for sample IRSNB 1150-M277b.

**Sample from *Prophoca rousseaui* IRSNB 1192-M276**

The preservation of the marine palynomorphs is moderate to good. A total of 24 dinocysts, two acritarch species and fungal remains were recorded.

The key dinocysts species in assemblage is *Unipontidinium aquaeductus*, which has a restricted occurrence in the middle Miocene of Europe (Dybkjaer & Piasecki, 2010) and the east coast of the USA (Verteuil & Norris 1996). Dybkjaer & Piasecki (2010) define in Denmark (eastern North Sea Basin) the *Unipontidinium aquaeductus* Zone as the body of strata from the lowest occurrence of the eponymous species to the lowest occurrence of *Achomosphaera andalousiensis*. The zone holds the entire stratigraphic range of *Unipontidinium aquaeductus*. According to Dybkjaer & Piasecki (2010), common species in the assemblage are *Apteodinium* spp., *Cleistosphaeridium placacanthum*, *Dapsilidinium pastielsii*, *Hystrichokolpoma rigaudiae*, *Labyrinthodinium truncatum*, *Lingulodinium machaerophorum*, *Operculodinium centrocarpum*, *Paleocystodinium golzowense*, *Spiniferites* spp. All latter species are recorded, while *Achomosphaera andalousiensis* is absent in the studied sample. The age of the *Unipontidinium aquaeductus* Zone is 14.8 Ma to 13.2 Ma (Dybkjaer & Piasecki, 2010).

The age of the sample can be further refined through the presence of *Habibacysta tectata*, a species not recorded by Dybkjaer & Piaseckii (2010), and with a lowest occurrence dated at 14.2 Ma (see above)

An age between 14.2 Ma and 13.2 Ma (late Langhian – early Serravallian) can be proposed for sample IRSNB 1192-M276.

**Sample from *Leptophoca proxima* IRSNB 1146-M279**

The preservation of the marine palynomorphs is moderate to poor and the diversity is low. A total of 14 dinocysts, three acritarch species and fungal remains were recorded.

The key dinocyst species in assemblage is *Unipontidinium aquaeductus*, which has a restricted occurrence in the middle Miocene from 14.8 Ma to 13.2 Ma (see above). The common species of the *U. aquaductus* Zone (see above) present in this sample are: *Cleistosphaeridium placacanthum, Hystrichokolpoma rigaudiae*, *Lingulodinium machaerophorum*, *Paleocystodinium golzowense* and *Spiniferites* spp. *Achomosphaera andalousiensis* is absent in the studied sample.

An age between 14.8 Ma and 13.2 Ma (middle Langhian – early Serravallian) can be proposed for sample IRSNB 1146-M279.

Table 1: recorded dinoflagellate cysts, acritarchs and fungal remains.

| **Dinoflagellate cysts** | **1150-M277b** | **1192-M276** | **1146-M279** |
| --- | --- | --- | --- |
| *Apteodinium tectatum* Piasecki 1980 |  | x |  |
| *Barssidinium graminosum* Lentin *et al*. 1994 | x |  |  |
| *Barssidinium pliocenicum* (Head 1993) | x |  |  |
| *Barssidinium* sp. ind. |  | x |  |
| *Batiacasphaera deheinzelinii* Louwye 1999 |  | x |  |
| *Batiacasphaera micropapillata* Stover 1977 | x | x |  |
| *Baticasphaera minuta* (Matsuoka 1983) | x | x | x |
| *Bitectatodinium tepikiense* Wilson 1973 |  |  | x |
| *Cleistosphaeridium placacanthum* (Deflandre & Cookson 1955) |  | x | x |
| *Cribroperidinium tenuitabulatum* (Gerlach 1961) |  |  | x |
| *Dapsilidinium pastielsii* (Davey & Williams 1966) | x | x |  |
| *Distatodinium paradoxum* (Brosius 1963) |  |  | x |
| *Habibacysta tectata* Head *et al*. 1989 | x | x |  |
| *Hystrichokolpoma rigaudiae* Deflandre and Cookson 1955 | x | x | x |
| *Hystrichosphaeropsis obscura* Habib 1972 | x |  |  |
| *Invertocysta lacrymosa* Edwards 1984 | x |  |  |
| *Labyrinthodinium truncatum* Piasecki 1980 | x | x |  |
| *Lejeunecysta challengerensis* Louwye 2008 |  | x |  |
| *Lingulodinium machaerophorum* (Deflandre & Cookson 1955) | x | x | x |
| *Melitasphaeridium choanophorum* (Deflandre & Cookson 1955) | x | x |  |
| *Operculodinium centrocarpum* (Deflandre & Cookson 1955) | x | x |  |
| *Operculodinium*? *eirikianum Head et al. 1989* | x |  |  |
| *Operculodinium israelianum* (Rossignol 1962) | x | x | x |
| *Palaeocystodinium golzowense* Alberti 1961 |  | x | x |
| *Quinquecuspis concreta* Harland 1977 | x | x | x |
| *Reticulatosphaera actinocoronata* (Benedek 1972) |  | x |  |
| Round brown cysts |  | x |  |
| *Selenopemphix brevispinosa* Head *et al*. 1989 | x | x | x |
| *Selenopemphix nephroides* Benedek 1972 | x |  |  |
| *Selenopemphix quanta* Bradford 1975 | x |  |  |
| *Spiniferites* spp. ind. | x | x | x |
| *Sumatradinium hamulatum* de Verteuil & Norrsi 1996 |  |  | x |
| *Tectatodinium pellitum* Wall 1967 | x | x |  |
| *Trinovantedinium* sp. ind. |  | x |  |
| *Tuberculodinium vancampoae* (Rossignol 1962) | x |  |  |
| *Unipontedinium aquaeductus* Piasecki 1980 |  | x | x |
| **Acritarchs** |  |  |  |
| *Cyclopsiella granosa* (Matsuoka 1983) |  | x | x |
| *Cyclopsiella? trematophora* (Cookson & Eisenack 1967) |  |  | x |
| *Paralecaniella indentata* (Deflandre & Cookson 1955) |  | x | x |
|  |  |  |  |
| **Fungal remains** |  | x | x |

**References**

Verteuil L de, Norris G. 1996. Miocene dinoflagellate cyst stratigraphy and systematics of Maryland and Virginia. *Micropaleontology* 42:1-172.

Dybkjær K, Piasecki S. 2010. Neogene dinocyst zonation for the eastern North Sea Basin, Denmark. *Review of Palaeobotany and Palynology* 161: 1-29. DOI: 10.1016/j.revpalbo.2010.02.005.

Fensome RA, McRae R, Williams GL. 2008. Dinoflaj2, Version1. *American Association of Stratigraphic Palynologists, Data Series* 1:1-937.

Louwye S, Head MJ, De Schepper S. 2004. Dinoflagellate cyst stratigraphy and palaeoecology of the Pliocene in northern Belgium, southern North Sea Basin. *Geological Magazine* 141:353-378. DOI: 10.1017/S0016756804009136.

Quaijtaal W, Donders TH, Persico D, Louwye S. 2014. Characterising the middle Miocene Mi-events in the Eastern North Atlantic realm: A first high-resolution marine palynological record from the Porcupine Basin. *Palaeogeography, Palaeoclimatology, Palaeoecology* 399: 140–159. DOI: 10.1016/j.palaeo.2014.02.017.

Schreck M, Matthiesen, J, Head MJ. 2012. A magnetostratigraphic calibration of Middle Miocene through Pliocene dinoflagellate cyst and acritarch events in the Iceland Sea (Ocean Drilling Program Hole 907A). *Review of Palaeobotany and Palynology* 187: 66–94. DOI: 10.1016/revpalbo.2012.08.006.
