# Supplementary material for: On Prophoca and Leptophoca (Pinnipedia, Phocidae) from the Miocene of the North Atlantic realm: redescription, phylogenetic affinities and paleobiogeographic implications
Source: PeerJ. 2017 Feb 21;5:e3024. doi: 10.7717/peerj.3024 (PMC5322758; doi:10.7717/peerj.3024)
Supplement: Supplemental Information 3 [file peerj-05-3024-s003.docx]

**Supplemental Information 3: Sacrum Measurements**

Table 1: Width across the sacral wings in relation to the width across the promontory of the sacrum of selected extinct (*Callophoca obscura*, *Phoca vitulinoides* and *Phocanella pumila*) and extant taxa. If applicable, sexes and age stages are indicated: ♀ = female, ♂ = male; j = juvenile, a = adult. Width measurements in mm. Lowest and highest ratios in bold.

| **Number** | **Taxon** | **Width across sacral wings**  **(1)** | **Width across promontory (2)** | **Ratio (1)/(2)** |
| --- | --- | --- | --- | --- |
| USNM 263623 | *Callophoca obscura*† | 146.6 | 43.4 | 3.38 |
| USNM 254248 | *Callophoca obscura*† | 147.2 | 41.7 | 3.53 |
| USNM 321887 | *Callophoca obscura*† | 150.0 | 45.5 | 3.30 |
| USNM 243703 | *Callophoca obscura*† | 122.4 | 36.2 | 3.38 |
| USNM 460231 | *Callophoca obscura*† | 161.4 | 55.3 | 2.92 |
| USNM 467720 | *Callophoca obscura*† | 162.2 | 56.8 | 2.86 |
| USNM 187602 | *Callophoca obscura*† | 129.2^1^ | 42.3 | 3.05 |
| USNM 263572 | *Callophoca obscura*† | 161.0 | 57.1 | 2.82 |
| USNM 460169 | *Callophoca obscura*† | 145.0 | 47.0 | 3.09 |
| USNM 244055 | *Callophoca obscura*† | 128.6 | 47.2 | 2.72 |
| USNM 329103 | *Callophoca obscura*† | 140.2 | 52.7 | 2.66 |
| USNM 467654 | *Callophoca obscura*† | 146.4 | 45.6 | 3.21 |
| USNM 360546 | *Callophoca obscura*† | 131.5 | 44.7 | 2.94 |
| USNM 421889 | *Callophoca obscura*† | 160.6 | 55.7 | 2.88 |
| USNM 302862 | *Callophoca obscura*† | 128.8 | 45.7 | 2.82 |
| USNM 250255 | *Callophoca obscura*† | 169.4 | 64.9 | 2.61 |
| USNM 392088 | *Callophoca obscura*† | 130.6 | 47.6 | 2.74 |
| USNM 364301 | *Callophoca obscura*† | 139.8 | 45.4 | 3.08 |
| USNM 360385 | *Callophoca obscura*† | 143.0 | 47.0 | 3.04 |
| IRSNB 12550 (♂) | *Halichoerus grypus* | 87.3 | 33.9 | 2.58 |
| USNM 504959 (♀, a) | *Histriophoca fasciata* | 95.2 | 32.7 | 2.91 |
| USNM 504960 (♂, a) | *Histriophoca fasciata* | 83.1 | 31.2 | 2.66 |
| USNM 571367 (♂, a) | *Histriophoca fasciata* | 86.8 | 28.3 | 3.07 |
| IRSNB 15388 (♀, a) | *Hydrurga leptonyx* | 149.4 | 60.5 | 2.47 |
| IRSNB 1164B (♂, a) | *Leptonychotes weddelli* | 132.1 | 48.0 | 2.75 |
| IRSNB 1163 (♀, a) | *Leptonychotes weddelli* | 117.2 | 44.0 | 2.66 |
| IRSNB 13307 (a) | *Leptonychotes weddelli* | 129.7 | 49.3 | 2.63 |
| IRSNB 1161 (♂, a) | *Lobodon carcinophaga* | 104.4 | 34.0 | 3.07 |
| IRSNB 1153 (a) | *Monachus monachus* | 119.7 | 42.0 | 2.85 |
| IRSNB 1164 (♂, a) | *Ommatophoca rossi* | 122.4 | 44.8 | 2.73 |
| IRSNB 15389 (♀, a) | *Ommatophoca rossi* | 119.6 | 36.7 | 3.26 |
| IRSNB 1555D (a) | *Pagophilus groenlandicus* | 107.0 | 36.7 | 2.92 |
| USNM 21535 (a) | *Pagophilus groenlandicus* | 93.2 | 30.4 | 3.07 |
| USNM 188766 (♂, a) | *Pagophilus groenlandicus* | 96.8 | 32.2 | 3.01 |
| USNM 504476 (♀, a) | *Pagophilus groenlandicus* | 94.0 | 34.3 | 2.74 |
| USNM 504207 (a) | *Pagophilus groenlandicus* | 98.2 | 32.4 | 3.03 |
| USNM 504475 (♂, a) | *Pagophilus groenlandicus* | 102.9 | 33.6 | 3.06 |
| IRSNB 7605 (♂, a) | *Phoca vitulina* | 89.0 | 29.3 | 3.04 |
| IRSNB **17592 (**♀, j) | ***Phoca vitulina*** | **63.5** | **29.0** | **2.19** |
| IRSNB 21240 (a) | *Phoca vitulina* | 82.0 | 29.5 | 2.78 |
| Gommers specimen^2^ | *Phoca vitulinoides*† | 51.9 | 17.9 | 2.90 |
| IRSNB-8243-13 | *Phoca vitulinoides*† | 54.4 | 18.6 | 2.92 |
| IRSNB-8243-07 | *Phoca vitulinoides*† | 59.5 | 19.2 | 3.10 |
| IRSNB 1092^3^ | *Phoca vitulinoides*† | 59.0 | 17.5 | 3.37 |
| IRSNB 1092^3^ | *Phoca vitulinoides*† | 57.3 | 19.8 | 2.90 |
| IRSNB 1059-M240b | *Phoca vitulinoides*† | 62.1 | 20.4 | 3.04 |
| USNM 421607 | *Phocanella pumila*† | 111.2 | 32.0 | 3.48 |
| USNM 437651 | *Phocanella pumila*† | 106.3 | 36.5 | 2.91 |
| **USNM 421539** | ***Phocanella pumila*†** | **119.7** | **31.9** | **3.75** |
| USNM 341615 (♂, a) | *Pusa caspica* | 69.6 | 23.1 | 3.01 |
| IRSNB 21170 (♀, a) | *Pusa sibirica* | 53.5 | 20.3 | 2.64 |
| IRSNB 15532 (♀, j) | *Pusa sibirica* | 47.3 | 20.0 | 2.37 |
| IRSNB 15264 (♀, a) | *Pusa sibirica* | 52.9 | 20.0 | 2.65 |

^1^ Specimen incomplete and true length significantly exceeds the measured length.

^2^ This specimen had been found in the private collection of mr. H. Gommers and is currently being described and currently not yet entered the collection of the IRSNB.

^3^ At the IRSNB, formerly unillustrated or undescribed specimens from the Van Beneden collection often share collection numbers.
