# Supplementary material for: On Prophoca and Leptophoca (Pinnipedia, Phocidae) from the Miocene of the North Atlantic realm: redescription, phylogenetic affinities and paleobiogeographic implications
Source: PeerJ. 2017 Feb 21;5:e3024. doi: 10.7717/peerj.3024 (PMC5322758; doi:10.7717/peerj.3024)
Supplement: Supplemental Information 4 [file peerj-05-3024-s004.docx]

**Supplemental Information 4: Coding of characters**

Note: References to characters adopted or modified from Amson & Muizon (2014) and Berta et al. (2015) and references therein provided.

Individual characters may also have been used in other publications not mentioned here.

**Cranial, mandibular and dental characters (unordered unless stated otherwise).**

1. Contact between premaxilla and nasal: (0) extensive; (1) reduced; (2) absent. (Berta & Wyss, 1994)
2. Premaxilla-maxilla suture: (0) lateral to nasal cavity; (1) at least partially included in nasal cavity. (Amson & Muizon, 2014, modified from Muizon, 1982; Berta et al., 2015, modified from Berta & Wyss, 1994; Wyss, 1988)
3. Facial angle: (0) snout more anteriorly than dorsally (angle 45° or more); (1) snout opening more dorsally than anteriorly (angle 45° or less).
4. Lateral border of opening of nasal cavity in lateral view: (0) rectilinear or weakly concave; (1) strongly concave. (Amson & Muizon, 2014)
5. Position of posterior end of nasals: (0) anterior to maxilla-frontal suture; (1) posterior to maxilla-frontal suture but greatly anterior to the level of the jugal-squamosal suture; (2) almost reaches the level of the jugal-squamosal suture (ordered). (Amson & Muizon, 2014, modified from Berta & Wyss, 1994)
6. Anterior end of frontals: (0) not inserted between nasals; (1) inserted medially between nasals. (Amson & Muizon, 2014, adopted from Berta & Wyss, 1994)
7. Alveolar process of maxilla: (0) facing ventrally; (1) facing anteroventrally posterior to P1. (Amson & Muizon, 2014)
8. Maxillary process of jugal (at level of the anteroventral border of the orbit) in lateral view: (0) thin and low and increasing progressively posteriorly; (1) thick and high and increasing abruptly posteriorly. (Amson & Muizon, 2014)
9. Position of anterior opening of infraorbital foramen in ventral view: (0) anterior to M1; (2) level or posterior to M1. (Amson & Muizon, 2014)
10. Anterior end of jugal in dorsal view: (0) lateral to infraorbital foramen; (1) above or medial to the lateral margin of the infraorbital foramen. (Amson & Muizon, 2014)
11. Ventral edge of the zygomatic arch, in anterior view: (0) higher than alveolar plane; (1) level with the alveolar plane (or very close to). (Amson & Muizon, 2014)
12. Mortised jugal-squamosal suture: (0) absent; (1) present. (Amson & Muizon, 2014, adopted from Berta & Wyss, 1994)
13. Supraorbital process of frontal: (0) absent; (1) present. (Amson & Muizon, 2014, modified from Berta & Wyss, 1994)
14. Least interorbital width: (0) occurs in posterior-most portion of interorbital bridge; (1) occurs in anterior half of interorbital bridge. (Amson & Muizon, 2014, adopted from Berta & Wyss, 1994)
15. Least interorbital widt/bizygomatic width ratio: (0) low (much lower than 0.3); (1) high (at least equal to 0.3). (Amson & Muizon, 2014; modified from Koretsky, 2001, Koretsky & Rahmat, 2013)
16. Major axes of glenoid fossae: (0) sub-parallel; (1) slightly convergent posteriorly.
17. Orientation of medial margins of tympanic bullae: (0) diverging posteriorly; (1) parasagittal. (Amson & Muizon, 2014)
18. Lateral end of tympanic bulla: (0) medial to level of mid-width of glenoid fossa; (1) lateral to level of mid-width of glenoid fossa. (Amson & Muizon, 2014)
19. Inflation of tympanic bulla: (0) weak; (1) strong. (Amson & Muizon, 2014; Wyss, 1988)
20. Posterior opening of the carotid canal: (0) visible in ventral view; (1) not visible in ventral view because of the strong inflation of the posterior part of the ectotympanic. (Amson & Muizon, 2014, modified from Berta & Wyss, 1994)
21. Posterior opening of the carotid canal and posterior lacerate foramen: (0) clearly separated; (1) coalescent. (Amson & Muizon, 2014, modified from Bininda-Emonds & Russell, 1996)
22. Posterior lacerate foramen: (0) small; (1) large and medial to tympanic bulla. (Amson & Muizon, 2014; King, 1966)
23. Posterior angle of the tympanic: (0) contacts the exoccipital posteriorly and is fused to it as well as to the mastoid; (1) anterior withdrawal of the tympanic which loses contact with the exoccipital; (2) posterior extension of the tympanic, which almost contacts the exoccipital but which still maintains a loose and smooth articulation with the petromastoid (ordered). (Amson & Muizon, 2014)
24. Dome-like elevation of the uncovered part of the petrosal posterior to the posterior edge of the tympanic: (0) absent; (1) present. (Amson & Muizon, 2014)
25. Heavily pachyosteosclerotic mastoid: (0) absent; (1) present. (Amson & Muizon, 2014, adopted from Berta & Wyss, 1994)
26. Mastoid visible in dorsal view: (0) no; (1) yes. (Amson & Muizon, 2014, adopted from Berta & Wyss, 1994)
27. Relation of paroccipital process to mastoid: (0) paroccipital process connected to mastoid by a high and continuous ridge; (1) paroccipital process well separated from mastoid. (Amson & Muizon, 2014, adopted from Berta & Wyss, 1994)
28. Petrosal apex: (0) 'V-shaped'; (1) rounded. (Amson & Muizon, 2014, modified from Wyss, 1988)
29. Petrosal: (0) not visible in posterior lacerate foramen; (1) visible in posterior lacerate foramen. (Berta & Wyss, 1994)
30. Relationship between facial nerve canal, fossa for vestibulocochlear nerve and roof of internal auditory meatus: (0) roof of internal auditory meatus present; (1) facial nerve and fossa for vestibulocochlear nerve separated. (Amson & Muizon, 2014, modified from Berta & Wyss, 1994)
31. Direction of occipital condyles in occipital view: (0) ventral; (1) diverging dorsally. (Amson & Muizon, 2014)
32. Orientation of pterygoids: (0) vertical; (1) laterally tilted and flaring posteriorly. (Amson & Muizon, 2014)
33. Alisphenoid canal: (0) absent; (1) present. (Amson & Muizon, 2014, adopted from Berta & Wyss, 1994)
34. Tooth rows: (0) parallel; (1) diverging posteriorly. (Amson & Muizon, 2014, modified from Berta & Wyss, 1994; modified from Koretsky, 2001, Koretsky & Rahmat, 2013)
35. Number of upper incisors: (0) six; (1) four. (Amson & Muizon, 2014, adopted from Muizon, 1982)
36. Number of lower incisors: (0) six; (1) four; (2) two. (Berta & Wyss, 1994; Koretsky, 2001; Koretsky & Rahmat, 2013)
37. Transverse groove on at least the mesial incisor: (0) present; (1) absent. (Amson & Muizon, 2014, modified from Berta & Wyss, 1994)
38. P,p2-M,m1: (0) at least one is single-rooted; (1) all are double-rooted. (Amson & Muizon, 2014, modified from Berta & Wyss, 1994)
39. M2: (0) present; (1) absent. (Amson & Muizon, 2014, adopted from Berta & Wyss, 1994)
40. Elevation of the labial cingulum below the main cusp of jugal teeth: (0) absent, (1) present. (Amson & Muizon, 2014)
41. Accessory cusps of jugal teeth: (0) very small or weakly developed (longer than high); (1) well developd (higher than long). (Amson & Muizon, 2014, modified from Muizon, 1981; modified from Koretsky, 2001, Koretsky & Rahmat, 2013)
42. Distolingual projection of cingulum of upper premolars: (0) absent; (1) present. (Amson & Muizon, 2014, modified from Muizon, 1981)
43. Anterior alveolar border of the upper incisors facing anteroventrally: (0) absent; (1) present. (Amson & Muizon, 2014)
44. Relative position of lower incisors: (0) mesial incisor posterior to lateral incisor; (1) mesial incisor level or lightly anterior to lateral incisor. (Amson & Muizon, 2014)
45. Shape of alveolus of lower canine: (0) circular to subcircular; (1) very elliptic. (Amson & Muizon, 2014)
46. Angular process of dentary: (0) large process protruding medially and posteriorly; (1) more a knob than a process and weakly protruding or not protruding medially. (Amson & Muizon, 2014)

**Postcranial characters (unordered unless stated otherwise).**

1. Atlas, transverse foramen: (0) visible in posterior view; (1) at least partially visible in dorsal view. (Amson & Muizon, 2014, modified from Wyss, 1988)
2. Atlas, direction of transverse process in lateral view: (0) oblique; (1) sub-vertical. (Amson & Muizon, 2014)
3. Cervical vertebrae 3-6, tubercle and lamina of transverse process: (0) fused or poorly isolated from each other; (1) clearly isolated from each other. (Amson & Muizon, 2014)
4. Scapula, acromion process: (0) knoblike; (1) reduced. (Amson & Muizon, 2014, adopted from Wyss, 1988)
5. Scapula, scapular spine I: (0) unreduced (i.e. the spine almost reaches the dorsal edge of the scapula; (1) reduced (i.e. the spine almost totally disappears and is reduced to its most proximal portion and the acromion). (Amson & Muizon, 2014, modified from King, 1966)
6. Scapula, supraspinous foss: (0) smaller than infraspinous fossa; (1) same size or larger than infraspinous fossa. (Berta & Wyss, 1994)
7. Humerus, deltopectoral crest: (0) long, (almost) reaching distal epiphysis; (1) short, stops approximately at mid-length of diaphysis. (Amson & Muizon, 2014, modified from Wyss, 1988; modified from Koretsky, 2001, Koretsky & Rahmat, 2013)
8. Humerus, deltopectoral crest: (0) anterior edge of humerus attenuates gradually, distally; (1) terminates abruptly distally.
9. Humerus, lesser tuberosity: (0) small (its distal end is distal to the level of the greater tuberosity); (1) well developed (its distal end is proximal to the level of the greater tuberosity). (Amson & Muizon, 2014; modified from Koretsky, 2001, Koretsky & Rahmat, 2013)
10. Humerus, supinator ridge: (0) well developed; (1) absent or poorly developed. (Amson & Muizon, 2014, adopted from Berta & Wyss, 1994)
11. Humerus, entepicondylar (=supracondylar) foramen: (0) present; (1) absent. (Amson & Muizon, 2014, adopted from King, 1966)
12. Radius, location of bicipital tuberosity: (0) on medial side; (1) on posteromedial side. (Amson & Muizon, 2014)
13. Radius, deep groove for *extensor digitorum communis* tendon: (0) absent; (1) present. (Amson & Muizon, 2014)
14. Ulna, distal end of styloid process: (0) distally pointed; (1) flattened. (Amson & Muizon, 2014)
15. Trapezium, styloid process (forming medially the scapholunar facet and laterally the facet for the carpale II): (0) long; (1) short. (Amson & Muizon, 2014)
16. Cuneiform, distally projecting ledge (palmar process) (Amson & Muizon, 2014, adopted from King, 1966): (0) absent; (1) present.
17. Metapodials, head: (0) keeled and trochleated phalangeal articulations; (1) smooth, with phalanges flat, articulations hinge-like. (Amson & Muizon, 2014, modified from Berta & Wyss, 1994)
18. Metacarpal I, length: (0) short; (1) long (much longer than half of the length of the radius). (Amson & Muizon, 2014, modified from King, 1966)
19. Manus, digit V, intermediate phalanx: (0) unreduced; (1) strongly reduced. (Amson & Muizon, 2014, adopted from King, 1966)
20. Manus claws: (0) large; (1) small. (Amson & Muizon, 2014, modified from King, 1966)
21. Innominate, ilium: (0) shallow gluteal fossa and weakly everted wing; (1) deep gluteal fossa and strongly everted wing. (Amson & Muizon, 2014)
22. Innominate, location of anterodorsal iliac spine: (0) dorsal to the anteroventral iliac spine; (1) posterodorsal to the anteroventral iliac spine. (Amson & Muizon, 2014)
23. Innominate, posteroventral iliac spine (=iliac tuberosity): (0) large and strongly protruding; (1) small or absent. (Amson & Muizon, 2014)
24. Innominate, proportions of postacetabular region (width/length): (0) long and narrow (ratio less than or equal to 0.5); (1) short and wide (ratio greater than 0.6). (Amson & Muizon, 2014, adopted from King, 1966)
25. Innominate, ischial spine: (0) unenlarged; (1) large. (Berta & Wyss, 1994)
26. Femur, trochanteric fossa: (0) relatively deep; (1) reduced or absent. (Berta et al., 2015, adopted from Berta & Wyss, 1994)
27. Femur, lesser trochanter: (0) present; (1) very reduced or absent. (Berta & Wyss, 1994)
28. Femur, collo-diaphyseal angle: (0) high, head oriented more medially than proximally; (1) low, head oriented more proximally than medially. (Amson & Muizon, 2014)
29. Femur, epicondyle crest: (0) inconspicuous or short; (1) long (reaching at least the mid-length of the diaphysis). (Amson & Muizon, 2014)
30. Femur, orientation of fossa for *m. peroneus longus*: (0) lateral; (1) anterolateral. (Amson & Muizon, 2014)
31. Femur, condyles: (0) different in size; (1) similar in size. (Berta et al., 2015, adopted from Koretsky and Grigorescu, 2002; Koretsky, 2001; Koretsky & Rahmat, 2013)
32. Femur, intercondylar region: (0) narrow and deep; (1) wide and flattened. (Berta et al., 2015, adopted from Koretsky and Grigorescu, 2002; Koretsky, 2001; Koretsky & Rahmat, 2013)
33. Femur, proportions (length/width): (0) long and narrow (ratio greater than 2.6); (1) short and wide (ratio less than 2.6). (Amson & Muizon, 2014)
34. Patella: (0) flat; (1) conical. (Berta & Wyss, 1994)
35. Tibia, post-tibial fossa: (0) weak; (1) strong. (Amson & Muizon, 2014, adopted from Berta & Wyss, 1994)
36. Tibia and fibula, proximal epiphysis: (0) unfused; (1) fused. (Berta & Wyss, 1994)
37. Astragalus, calcaneal process: (0) absent; (1) poorly developed; (2) well developed (ordered). (Amson & Muizon, 2014, modified from Berta & Wyss, 1994)
38. Sustentacular facet of the astragalus: (0) oval-shaped and narrowed at contact with cuboid facet; (1) long (at least twice longer than wide), slender and strongly bent medially; (2) short and tongue-like with no narrowing at contact with cuboid facet. (Amson & Muizon, 2014)
39. Calcaneum, articular surface for fibula: (0) absent or very reduced; (1) well developed. (Amson & Muizon, 2014)
40. Calcaneum, secondary shelf: (0) absent; (1) present. (Berta & Wyss, 1994)
41. Metatarsal I, articular surface for metatarsal II: (0) oriented laterally; (1) oriented dorsolaterally; (2) inconspicuous. (Amson & Muizon, 2014)
42. Metatarsal III, length: (0) less than 50% shorter than metatarsal I; (1) approximately 50% shorter (or more) than metatarsal I. (Amson & Muizon, 2014, modified from King, 1966)
43. Pes claws: (0) large; (1) small. (Amson & Muizon, 2014, modified from King, 1966)

**References**

Amson E, Muizon C de. 2014. A new durophagous phocid (Mammalia: Carnivora) from the late Neogene of Peru and considerations on monachine seals phylogeny. *Journal of Systematic Palaeontology* 12:523-548. DOI: 10.1080/14772019.2013.799610.

Berta A, Kienle S, Bianucci G, Sorbi S. 2015. A Reevaluation of *Pliophoca etrusca* (Pinnipedia, Phocidae) from the Pliocene of Italy: Phylogenetic and Biogeographic Implications. *Journal of Vertebrate Paleontology* 35(1):e889144. DOI: 10.1080/02724634.2014.889144.

Berta A, Wyss AR. 1994. Pinniped phylogeny. *Proceedings of the San Diego Society of Natural History* 29:33-56.

Bininda-Emonds ORP, Russell AP. 1996. A morphological perspective on the phylogenetic relationships of the extant phocid seals (Mammalia: Carnivora: Phocidae). *Bonner zoologische Monographien* 41:1-256.

King JE. 1966. Relationships of the hooded and elephant seals (genera *Cystophora* and *Mirounga*). *Journal of Zoology* 148:385-398.

Muizon C. de 1982. Phocid phylogeny and dispersal. *Annals of the South African Museum* 89:175-213.

Nojima T. 1990. A morphological consideration of the relationships of pinnipeds to other carnivorans based on the bony tentorium and bony falx. *Marine Mammal Science* 6:54-74.

Weckerly W. 1998. Sexual-size dimorphism: influence of mass and mating systems in the most dimorphic mammals. *Journal of Mammalogy* 79:33-52.

Wyss AR. 1988. On “retrogression” in the evolution of the Phocinae and phylogenetic affinities of the monk seal. *American Museum Novitates* 2924:1-38.

Koretsky IA. 2001. Morphology and systematics of Miocene Phocinae (Mammalia: Carnivora) from Paratethys and the North Atlantic regions. *Geologica Hungarica Series Palaeontologica* 54:1-109.

Koretsky IA, Grigorescu D. 2002. The Fossil Monk Seal *Pontophoca sarmatica* (Alekseev)(Mammalia: Phocidae: Monachinae) from the Miocene of Eastern Europe. In: Emry RJ, ed. *Cenozoic Mammals of Land and Sea: Tributes to the Career of Clayton E. Ray. Smithsonian Contributions to Paleobiology* 93:149-162.

Koretsky, IA, Rahmat SJ. 2013. First record of fossil Cystophorinae (Carnivora, Phocidae): middle Miocene seals from the northern Paratethys. *Rivista Italiana di Paleontologia e Stratigrafia* 119:325-350.
