# Supplementary material for: On Prophoca and Leptophoca (Pinnipedia, Phocidae) from the Miocene of the North Atlantic realm: redescription, phylogenetic affinities and paleobiogeographic implications
Source: PeerJ. 2017 Feb 21;5:e3024. doi: 10.7717/peerj.3024 (PMC5322758; doi:10.7717/peerj.3024)
Supplement: Supplemental Information 5 [file peerj-05-3024-s005.docx]

**Supplemental Information 5: phylogenetic character matrix**

Note: ‘?’ refers to missing data; ‘-‘ refers to gaps.

*Enaliarctos_mealsi* 000?101?0?000010000010000000?0001110010?11??0001?01100001100?00?1?011100000?1001000??0?0-

*Thalassoleon_mexicanus* 001011000000100000001?000010?00010010100000?00???00100011010?011??0110010000110?000000?00

*Otaria_flavescens* 00000100000010000000100000100000100100000000000000010001101000111101100100001101010001200

*Arctocephalus_pusillus* 00000100000010000000100000100000100100000000000000010001101000111101100100001101010001200

*Odobenus_rosmarus* 10000000-0?00000001010000010001011--?010000--01100010001101001111101111110001101001000200

*Allodesmus_kernensis* 111010001011000001100020002?10001101?00000100101?001010101000001?1010001100?0101001??020?

*Kawas_benegasorum* ?????????????????????????????????????????????????10?00?00?????0000110?10110??11?????????0

*Phoca_vitulina* 10101000110101010121011011211111010111100000010011001110010001000011001010000000112100000

*Halichoerus_grypus* 10101000100101010121011011211111010110100000010011001110010001000011001010000000112100000

*Erignathus_barbatus* 10111000100101010121001011211111010111100010010011001110010101000001001010000000112100000

*Monachus_monachus* 11111000110100000120001110210111011111100000010101010011100100110100111010111000012200111

*Pliophoca_etrusca* ?111100?????0?????????1110?10??1?111111?000?01??????001110?10?1????????0101110?0?122?011?

*Mirounga_leonina* -11110001101000001200020102111110112101-000-010101010011100110110100101?1111??10012200111

*Callophoca_obscura* ????????????????????????????????????????????????????0011100???????0?1??011100110012200??1

*Hadrokirus_martini* 1111101111010000112000201021??110111111101000110????????????????????????????????0?21????1

*Piscophoca_pacifica* 2111101111010000112000201021??110111?11101001111?101000110110011??00111?10???0??0121101?1

*Acrophoca_longirostris* 111110001111000011200020102111110111111101000110011100011011001???00111110111010012210111

*Homiphoca_capensis* 1111101011110010112000201021111101111111011001??????00100001??1???001?001011001?1121101??

*Leptonychotes_weddelli* 11111000001100001120002010211111011111100000111101111011110110111100111110110010012210111

*Lobodon_carcinophaga* 11102000001100101120002010211111011111111111110101111011110110111100111010110110012210111

*Hydrurga_leptonyx* 21112000001100001120002010211111011111111101111101110011110110111100111110110010012210111

*Ommatophoca_rossi* 2111-0100011001011200?2011211111011111110000011101110011110110111100111110110110012210111

*Prophoca_rousseaui* ????????????????????????????????????????????????????0(01)0??11???????010??01?00011?1????????

*Leptophoca_lenis* ?????????????????????????????????????????????????????1000111??????0100100000001?11???????

*Afrophoca_lybica* ?????????????????????????????????0???1???????????????????????????????????????????????????

*Properiptychus_argentinus* ?????????????????????????????????1???1?101??????????0001101?????????1??01011?10??????10??

*Australophoca_changorum* ????????????????????????????????????????????????????00111?1????????????010????????2111???
